# Nickel-Catalyzed Four-Component Carbonylation of 1,3-Butadiene To Access β,γ-Unsaturated Ketones

**DOI:** 10.1021/acs.orglett.4c01599

**Published:** 2024-05-29

**Authors:** Bing-Hong Teng, Zhi-Peng Bao, Yingying Zhao, Xiao-Feng Wu

**Affiliations:** † Dalian National Laboratory for Clean Energy, Dalian Institute of Chemical Physics, Chinese Academy of Sciences, Dalian 116023, Liaoning, China; ‡ School of Chemistry and Chemical Engineering, Liaoning Normal University, 850 Huanghe Road, Dalian 116029, China; § Leibniz-Institut für Katalyse e.V., Albert-Einstein-Straße 29a, 18059 Rostock, Germany

## Abstract

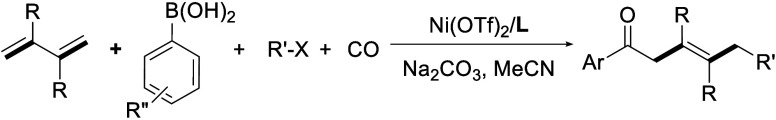

A new strategy to obtain β,γ-unsaturated ketones via the cross-coupling of 1,3-butadiene, alkyl bromides, and arylboronic acids under 1 bar of CO with nickel as the catalyst has been developed. This newly developed four-component carbonylation procedure features advantages including using a cheap catalytic system, high step economy, mild reaction conditions, and excellent 1,4-regioselectivity, thereby providing a sustainable and alternative tool for β,γ-unsaturated ketones production compared to the present tactics. To elucidate the application potential of this method, olefin synthons are derived from the representative coupling product.

β,γ-Unsaturated ketone structural motifs are found in many bioactive molecules and natural products, acting as organic synthesis intermediates and the basic backbone for building complex structures.^1^ However, the synthesis of β,γ-unsaturated ketones is still challenging because of the thermodynamically favored isomerization of C=C bonds to α,β-unsaturated ketones.^2^ Many of the reported methods are based on α-alkenylation of ketones for synthesizing β,γ-unsaturated ketones (Figure 1a),^3^ most of them requiring prefabrication of substrates, multistep reactions, or postprocessing, which limit product structural diversity. Therefore, convenient and efficient new methods for the synthesis of β,γ-unsaturated ketones are always demanded.

**Figure 1:**
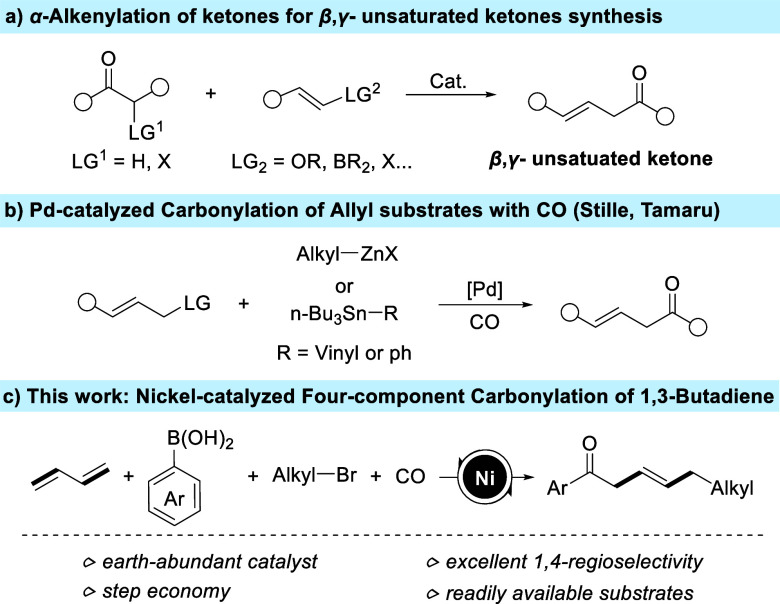
Strategies for β,γ-unsaturated ketones preparation.

Carbonylation using carbon monoxide (CO) as the C1 source is one of the most economical and convenient tools to construct carbonyl containing compounds.^4^ For example, Tamaru and Stille have achieved Pd-catalyzed carbonylation of allyl substrates with CO gas to access β,γ-unsaturated ketones (Figure 1b).^5^ However, the use of organotin reagents or organozinc coupling partners is certainly restricted to some functional groups. Moreover, these methods usually suffer from low regioselectivity for some materials. 1,3-Butadiene, the simplest conjugated diene in nature, is a low-cost and abundant carbon source produced from liquid cracking, and its unique molecular structure H_2_C=CH–CH=CH_2_ offers a wide range of possibilities for chemical transformation.^6^ Therefore, 1,3-butadiene is an attractive surrogate to access the allyl substrates.^7^

Nickel is one of the earth-abundant, nontoxic, and environmentally friendly metals among various transition metals and is the preferred material for tandem radical processes. In the catalytic carbonylation field, the palladium catalytic system has been well-established, while nickel-catalyzed carbonylation is underdeveloped. The reason for this is that the strong binding affinity between CO and nickel prevents nickel from interacting with the substrates.^8^ We sought to develop a convenient synthetic method toward β,γ-unsaturated ketones through cross-coupling to construct three C–C bonds directly. To validate the conceptual framework mentioned above, we achieved a Ni-catalyzed highly regionally selective four-component carbonylation of 1,3-butadiene, arylboronic acids, alkyl bromides, and 1 bar of CO (Figure 1c). This novel methodology features an inexpensive catalytic system, relatively mild reaction conditions, excellent 1,4-regioselectivity, commercially available materials, and high step economy.

To evaluate the practicality of the above-described avenue, we performed systematic optimization studies of this nickel-catalyzed carbonylation with 1,3-butadiene **1a**, aryboronic acid **2a**, and ethyl bromodifluoroacetate **3a** as the model substrates (Table 1). The targeted β,γ-unsaturated ketone **4aa** was obtained in 40% yield using Ni(OTf)_2_ as catalyst, 4,7-diphenyl-1,10-phen as ligand, and Na_2_CO_3_ as base, in 1,4-dioxane at 70 °C under a CO (1 bar) atmosphere for 20 h (Table 1, entry 1). Among the ligands evaluated, slightly decreased yields of the target product were obtained with **L2** or **L3**, and the other tested ligands inhibited the desired coupling reaction (Table 1, entries 2–6). Other precatalysts including Ni(hfac)_2_ and Ni(acac)_2_ showed no catalytic activity (Table 1, entries 7–8). The nonstrong bonding of the OTf^–^ anion with nickel might can favor its activation and then get ready to catalyze substrates which lead to the difference in results. Screening of solvents suggested that MeCN was the best media for the reaction, while DCE and THF reduced the yield of compound **4aa** (Table 1, entries 9–11). In addition, the proportion of reactant and reaction time were adjusted to increase the yield of **4aa** to 66% by using **1a** (0.4 mmol), Na_2_CO_3_ (2.0 equiv), and extending the reaction time to 24 h (Table 1, entries 12–14). Finally, the dosage of catalyst and ligand was increased to 10 mol %, and the isolated yield of **4aa** can be further improved to 72% (Table 1, entry 15). It is worth mentioning that N_2_ was added to prevent the escape of 1,3-butadiene gas from the reaction solution. A very low yield of **4aa** was obtained in the absence of N_2_ or under higher pressure of CO which will inhibit nickel’s catalytic activity due to its strong coordination with nickel. Additionally, a small amount of 1,3-butadiene dimerization compound could be detected which consumed some substrate and led to a decreased yield of the desired product.

**Table 1:**
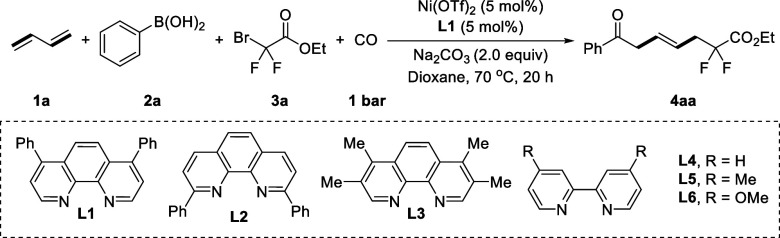
Optimization of the Reaction Conditions^a^

entry	catalyst	ligand	solvent	yield (%)^b^
1	Ni(OTf)_2_	**L1**	Dioxane	40
2	Ni(OTf)_2_	**L2**	Dioxane	20
3	Ni(OTf)_2_	**L3**	Dioxane	29
4	Ni(OTf)_2_	**L4**	Dioxane	NR
5	Ni(OTf)_2_	**L5**	Dioxane	NR
6	Ni(OTf)_2_	**L6**	Dioxane	trace
7	Ni(hfac)_2_	**L1**	Dioxane	trace
8	Ni(acac)_2_	**L1**	Dioxane	NR
9	Ni(OTf)_2_	**L1**	MeCN	45
10	Ni(OTf)_2_	**L1**	DCE	trace
11	Ni(OTf)_2_	**L1**	THF	trace
12^c^	Ni(OTf)_2_	**L1**	MeCN	48
13^d^	Ni(OTf)_2_	**L1**	MeCN	52
14^e^	Ni(OTf)_2_	**L1**	MeCN	66
15^f^	Ni(OTf)_2_	**L1**	MeCN	74 (72)

a Reaction conditions: **1a** (0.3 mmol), **2a** (0.2 mmol), **3a** (0.3 mmol), Na_2_CO_3_ (1.5 equiv), Ni(OTf)_2_ (5 mol %), **L1** (5 mol %), CO (1 bar), N_2_ (5 bar), Dioxane (1.0 mL), 70 °C, 20 h.

b Yield was determined by GC the isolated yield is given in parentheses. NR = no reaction.

c **1a** (0.4 mmol).

d Na_2_CO_3_ (2.0 equiv).

e 24 h.

f Ni(OTf)_2_ (10 mol %), **L1** (10 mol %).

Having identified conditions to achieve the targeted carbonylative transformation, we started to investigate the substrates scope of this transformation. First, the scope with respect to arylboronic acid **2**, butadiene **1a**, and ethyl bromodifluoroacetate **3a** was examined (Scheme 1). Notably, the alkyl or phenyl group at the 4-position of arylboronic acids proceeded smoothly to provide the corresponding β,γ-unsaturated ketone **4ab** and **4ac** in good yields. Arylboronic acids with an electron donor group at the *para* position were found to be competent substrates, providing **4ad**–**4ag** in moderate to excellent yields with exclusive 1,4-selectivities. It is worth noting that the thioether unit (**4ag**) was compatible with the reaction, as well. To our delight, electron-withdrawing acetyl and ester groups were well suited to the process, and the yield of **4ai** was up to 74%. Notably, the halogen atoms remained intact, and thus, the products **4aj**–**4al** offer the possibility for further cross-coupling reactions. This strategy was also suitable for base-sensitive furanboronic acid to give product **4am** in 31% yield.

**Scheme 1:**
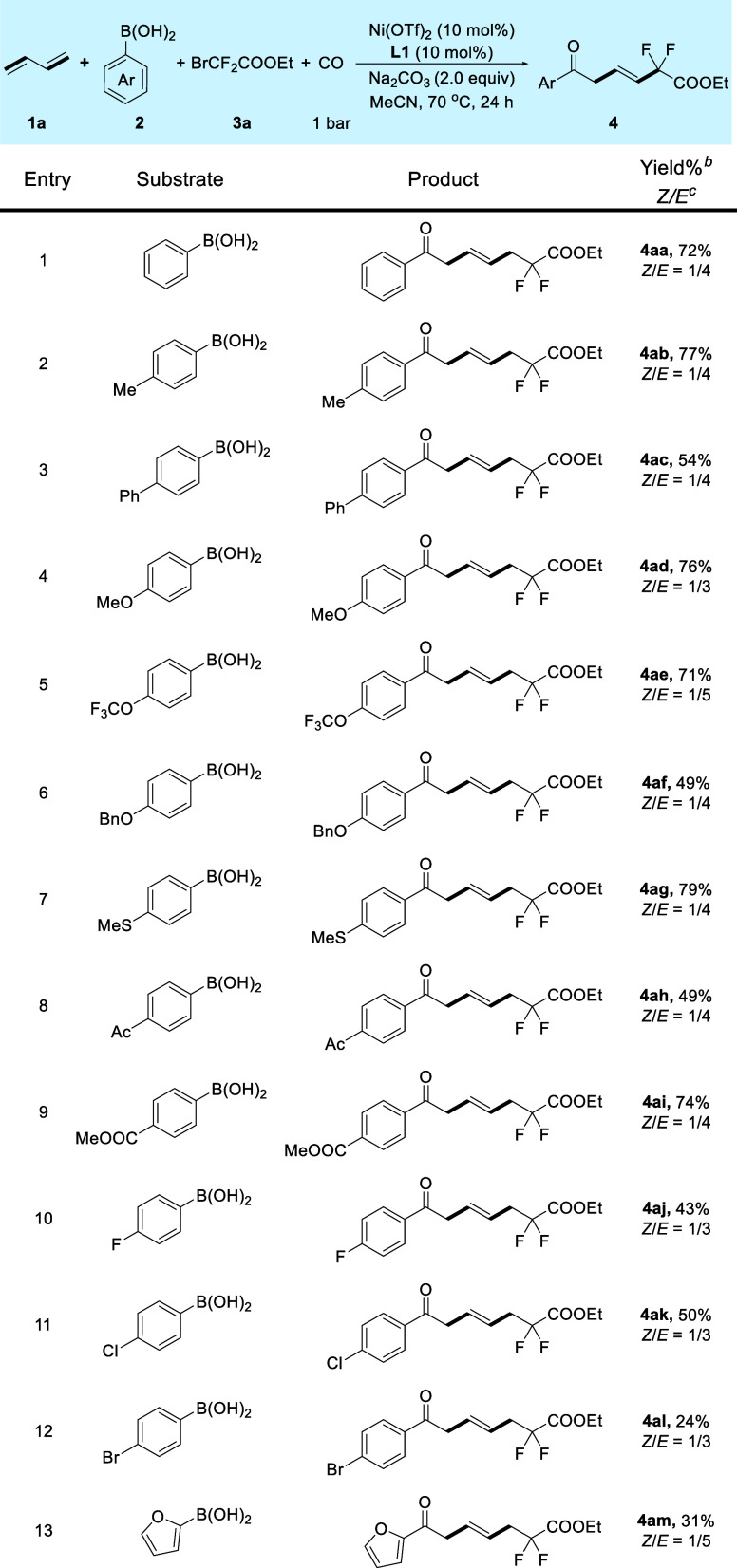
Substrate Scope of Boronic Acids Reaction conditions: **1a** (0.4 mmol), **2a** (0.2 mmol), **3a** (0.3 mmol), Na_2_CO_3_ (2.0 equiv), Ni(OTf)_2_ (10 mol %), **L1** (10 mol %), CO (1 bar), N_2_ (5 bar), MeCN (1.0 mL), 70 °C, 24 h. Isolated yields. The isomeric ratio of *Z* to *E* was determined by ^1^H NMR analysis.

The scope of the alkyl radical precursors was subsequently investigated (Scheme 2). Benzyl 2-bromo-2,2-difluoroacetate substrate yielded the corresponding product **4ba** in 79% yield. Bromofluoroacetate (**3b**), bromodifluoroacetamide (**3c**), and perfluoroalkyl iodine (**3d**) can be applied and gave the desired four-component carbonylation with 1,3-butadiene and phenylboronic acid. Surprisingly, this reaction can also be extended to nonfluorinated substrates and the corresponding products **4be** and **4bf** were obtained successfully despite relatively low yields. Notably, a cyclopropane analogue of product **4bg** was isolated in 47% yield in the reaction with ethyl 2,2-dibromo-2-fluoroacetate. Additionally, 2,2-dimethyl-substituted butadiene is also a suitable substrate for this reaction and delivered the corresponding product **4ca** in 37% yield.

**Scheme 2:**
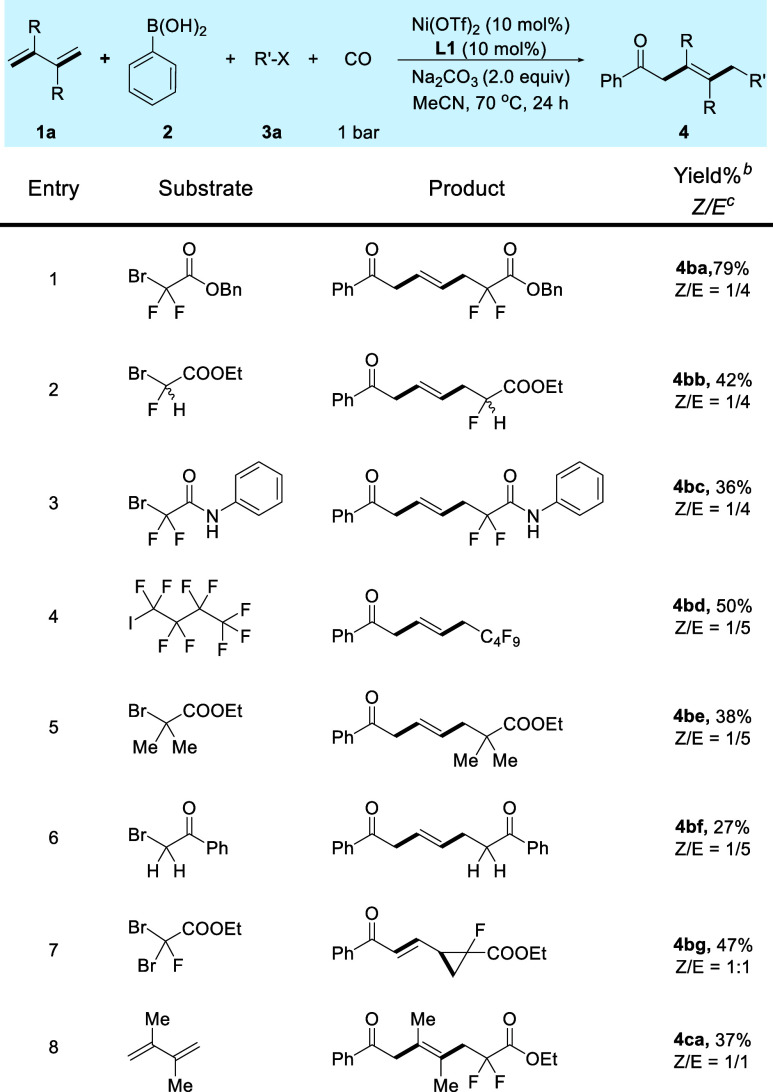
Substrate Scope of Radical Precursors and Conjugated Diene Reaction conditions: **1a** (0.4 mmol), **2a** (0.2 mmol), **3a** (0.3 mmol), Na_2_CO_3_ (2.0 equiv), Ni(OTf)_2_ (10 mol %), **L1** (10 mol %), CO (1 bar), N_2_ (5 bar), MeCN (1.0 mL), 70 °C, 24 h. Isolated yields. The isomeric ratio of *Z* to *E* was determined by ^1^H NMR analysis.

To unravel the mechanism of this nickel-catalyzed four-component reaction, we carried out radical inhibition experiments to probe the possible reaction pathway. By adding 2,2,6,6-tetramethyl-1-piperinedinyloxy (TEMPO, 3 equiv) into the model reaction, the reaction was significantly inhibited and led to no desired product being detected (Scheme 3a). This result indicates that a radical intermediate was most likely involved during the process of this transformation. To demonstrate the applicability of the disclosed protocol for late-stage, scale-up and derivation experiments were carried out. Scaled up carbonylation to 2.0 mmol with 1.0 equiv of butadiene was carried out, and the desired product **4aa** can still be formed in 66% yield. Meanwhile, the β,γ-unsaturated ketone can be transformed into other useful molecules. For example, the treatment of **4aa** with hydroxylamine hydrochloride can produce β,γ-unsaturated ketoxime **5aa**. Both the ester and carbonyl groups of **4aa** were able to be reduced by sodium borohydride and produce the reduced product **5ab** in 70% yield. Additionally, the C=C of **4aa** could be selectively reduced to produce **5ac** by hydrogenation.

**Scheme 3:**
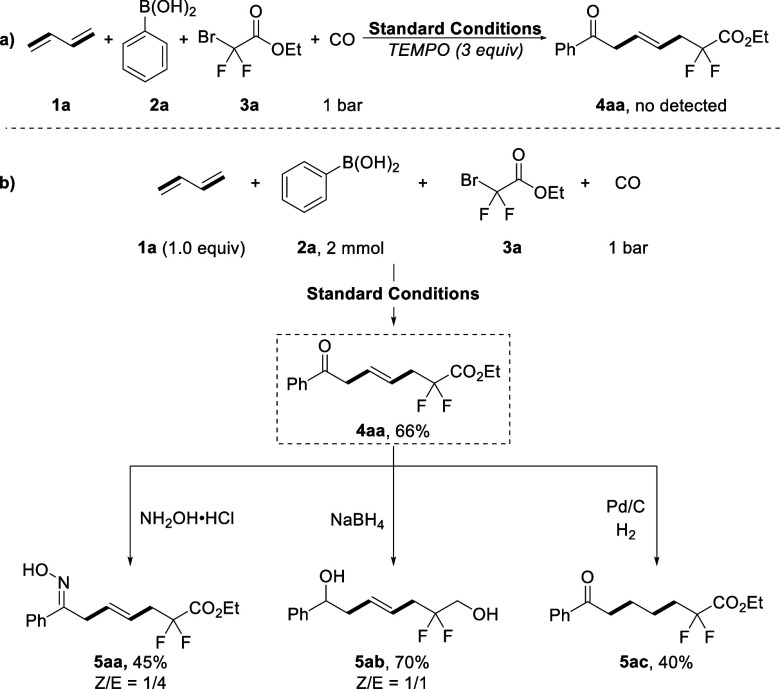
Radical Inhibition Experiments and Transformations of β,γ-Unsaturated Ketones

Based on reaction mechanism studies and previous reports,^9^ we proposed a possible reaction pathway as shown in Scheme 4. At the beginning of this cascade reaction, the Ni^II^ complex with arylboronic acid **2** undergoes transmetalation to form aryl nickel intermediate **B**. CO migrates into **B** to generate acyl nickel intermediate **C**. Single electron transfer (SET) between **C** and the radical precursor generates the Ni^III^Ln complex and radical **E**. Comproportionation between Ni^III^Ln with Ni^I^Ln forms Ni^II^Ln intermediate **C**. Next, the radical was trapped by butadiene to give the radical intermediate **F** as a secondary carbon radical, which undergoes isomerization to deliver the more reactive primary carbon radical intermediate **G** via an allylic intermediate. Subsequently, **G** reacts with complex **C** to obtain the key Ni^III^ intermediate **H**. An eventual reductive elimination of **H** gives Ni^I^Ln to close the catalytic cycle and also gives the final product **4**.

**Scheme 4:**
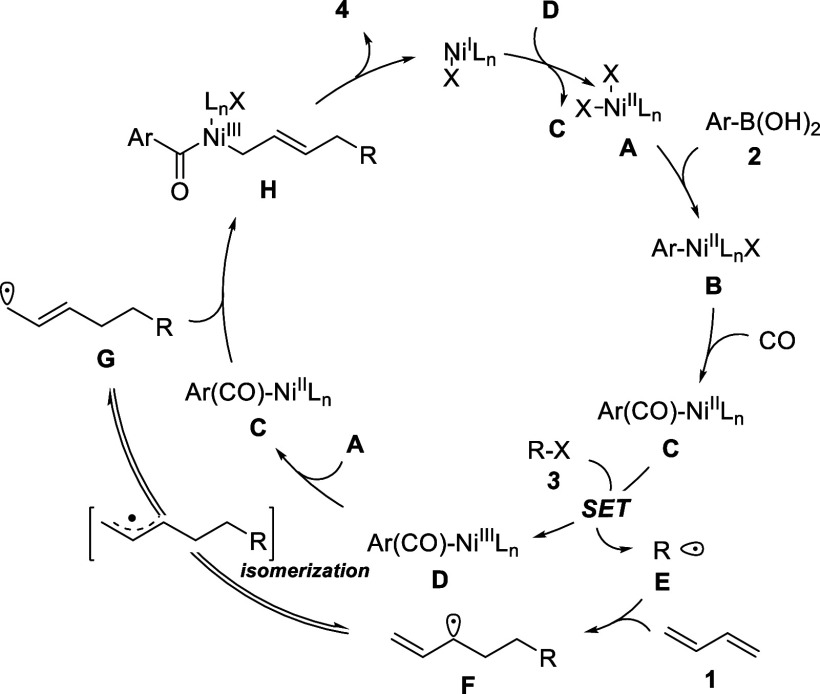
Proposed Mechanism

In conclusion, we have developed a nickel-catalyzed multicomponent coupling of 1,3-butadiene, arylboronic acids, alkyl bromides, and CO gas. This protocol features a cheap catalytic system, high step economy, mild reaction conditions, and excellent 1,4-regioselectivity. This reaction provides an efficient method for the synthesis of structurally diverse β,γ-unsaturated ketones. More importantly, the resulting products can also undergo subsequent transformations to furnish olefin synthons.

## Data Availability

The data underlying this study are available in the published article and its Supporting Information.

## Supporting Information Available

General comments, general procedure, optimization details, analytic data and NMR spectra (PDF)
