# Supplementary material for: Nickel-Catalyzed Four-Component Carbonylation of 1,3-Butadiene To Access β,γ-Unsaturated Ketones
Source: Org Lett. 2024 May 29;26(22):4779–83. doi: 10.1021/acs.orglett.4c01599 (PMC11165585; doi:10.1021/acs.orglett.4c01599)
Supplement: Supplementary file 1 — ol4c01599_si_001.pdf [file ol4c01599_si_001.pdf]

Supporting Information

Nickel-catalyzed four-component carbonylation of 1,3-butadiene to access *β*,*γ*-unsaturated ketones

Bing-Hong Teng,[a,b] Zhi-Peng Bao,[a,c] Yingying Zhao,[b] Xiao-Feng Wu[a,c,*]

[a] Dalian National Laboratory for Clean Energy, Dalian Institute of Chemical Physics, Chinese Academy of Sciences, Dalian 116023, Liaoning, China, E-mail: xwu2020@dicp.ac.cn

[b] School of Chemistry and Chemical Engineering, Liaoning Normal University, 850 Huanghe Road, Dalian 116029 China

[c] Leibniz-Institut für Katalyse e.V., Albert-Einstein-Straße 29a, 18059 Rostock, Germany

Table of Contents

1. General Information... S2

2. Optimization of Reaction Conditions S3

3. Preparation of Substrates S4

4. General Procedure for Carbonylation of 1,3-Butadiene................ S4

5. Derivatization Reactions S5

6. Mechanistic Studies S6

7. Spectroscopic Data of Products.. S7

8. NMR Spectra of the Products.. S7

9. Reference S60

1. General Information

Unless otherwise noted, all reactions were carried out under a carbon monoxide or nitrogen atmosphere. The reagents were ordered from Adamas-beta®, Energy Chemical Sigma-Aldrich, Bidepharm and used without purification. All solvents were dried by standard techniques and distilled prior to use. Column chromatography was performed on silica gel (200-300 meshes). All NMR spectra were recorded at ambient temperature using Bruker Avance III 400 MHz NMR (^{1}H, 400 MHz; ^{13}C{^{1}H}, 101 MHz, ^{19}F 376 MHz), Bruker AVANCE III HD 700MHz NMR spectrometers (^{1}H, 700 MHz; ^{13}C{^{1}H}, 100 MHz). ^{1}H NMR chemical shifts are reported relative to TMS and were referenced via residual proton resonances of the corresponding deuterated solvent ($CDCl_3$: 7.26 ppm) whereas ^{13}C{^{1}H} NMR spectra are reported relative to TMS via the carbon signals of the deuterated solvent ($CDCl_3$: 77.0 ppm). Data for ^{1}H are reported as follows: chemical shift (δ ppm), multiplicity (s = singlet, d = doublet, t = triplet, q = quartet, quint = quintet, m = multiplet, td = triple doublet, br = broad), coupling constant (Hz), and integration. All ^{13}C NMR spectra were broad-band ^{1}H decoupled. All reactions were monitored by GC-FID or NMR analysis. HRMS data was obtained with Micromass HPLC-Q-TOF mass spectrometer (ESI) or Agilent 6540 Accurate-MS spectrometer (Q-TOF).

Because of the high toxicity of carbon monoxide, all the reactions should be performed in an autoclave. The laboratory should be well-equipped with a CO detector and alarm system.

2. Optimization of Reaction Conditions

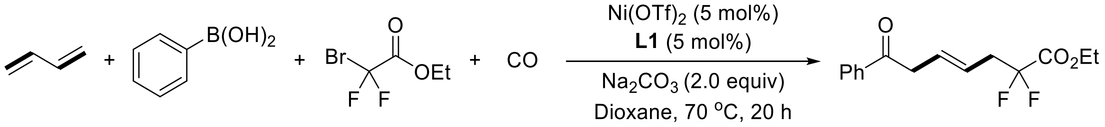

1a 2a 3a 1 bar 4aa

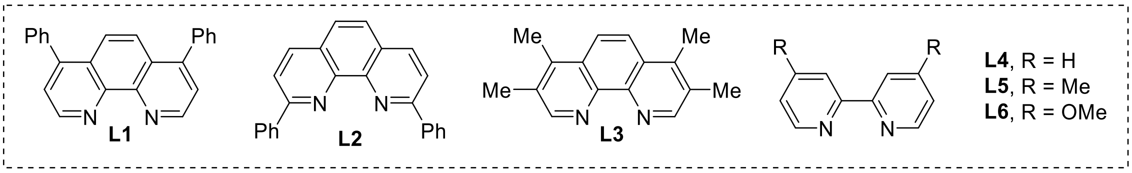

Entry	Catalyst	Ligand	Base	Solvent	Yield (%)[b]
1	$Ni(OTf)_2$	**L1**	Na_2CO_3	Dioxane	40
2	$Ni(OTf)_2$	**L2**	Na_2CO_3	Dioxane	20
3	$Ni(OTf)_2$	**L3**	Na_2CO_3	Dioxane	29
4	$Ni(OTf)_2$	**L4**	Na_2CO_3	Dioxane	NR
5	$Ni(OTf)_2$	**L5**	Na_2CO_3	Dioxane	NR
6	$Ni(OTf)_2$	**L6**	Na_2CO_3	Dioxane	trace
7	$Ni(OTf)_2$	**tpy**	Na_2CO_3	Dioxane	NR
8	$Ni(hfac)_2$	**L1**	Na_2CO_3	Dioxane	trace
9	$Ni(acac)_2$	**L1**	Na_2CO_3	Dioxane	NR
10	$Ni(dppe)Cl_2$	**L1**	Na_2CO_3	Dioxane	NR
11	$NiCl_2{\cdot}DME$	**L1**	Na_2CO_3	Dioxane	NR
12	$Ni(OTf)_2$	**L1**	$NaHCO_3$	Dioxane	10
13	$Ni(OTf)_2$	**L1**	Na_3PO_4	Dioxane	8
14	$Ni(OTf)_2$	**L1**	K_2CO_3	Dioxane	NR
15	$Ni(OTf)_2$	**L1**	NaOAc	Dioxane	trace
16	$Ni(OTf)_2$	**L1**	NEt_3	Dioxane	24
17	$Ni(OTf)_2$	**L1**	Na_2CO_3	MeCN	45
18	$Ni(OTf)_2$	**L1**	Na_2CO_3	DCE	trace
19	$Ni(OTf)_2$	**L1**	Na_2CO_3	THF	trace
20	$Ni(OTf)_2$	**L1**	Na_2CO_3	MTBE	NR
21[c]	$Ni(OTf)_2$	**L1**	Na_2CO_3	MeCN	48
22[d]	$Ni(OTf)_2$	**L1**	Na_2CO_3	MeCN	52
23[e]	$Ni(OTf)_2$	**L1**	Na_2CO_3	MeCN	66
24[f]	$Ni(OTf)_2$	**L1**	Na_2CO_3	MeCN	74(72)

[a]Reaction conditions: **1a** (0.3 mmol), **2a** (0.2 mmol), **3a** (0.3 mmol), Na_2CO_3 (0.3 mmol), $Ni(OTf)_2$ (5 mol%), **L1** (5 mol%), CO (1 bar), N_2 (5 bar), Dioxane (1.0 mL), 70 °C, 20 h. [b]Yield was determined by GC the isolated yield is given in parentheses. NR = no reaction. [c]**1a** (0.4 mmol). [d]Na_2CO_3 (2.0 equiv). [e]24 h. [f]$Ni(OTf)_2$ (10 mol%), **L1** (10 mol%).

3. Preparation of Substrates

General procedure for difluorobromoacetic acid derivative[1]:

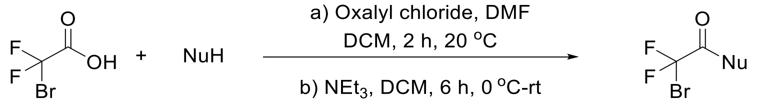

Step a: 2-bromo-2,2-difluoroacetic acid (1 equiv.) was dissolved in dry DCM in a dry round bottom flask under an atmosphere of nitrogen. Oxalyl chloride (1.1 equiv) was then added slowly into reaction system over 5 min with stirring at 20 °C. Two drops of DMF were then added, and the reaction mixture was stirred at 20 °C for further 2 h.

Step b: Then a mixture of alcohol or amine (1.1 equiv) and Et_3N (1.1 equiv) dissolved in dry DCM (15 mL) was added dropwise to the reaction mixture at 0 °C. The cooling was removed and stirring was continued for 6 hours. After the reaction was over, water was added to the reaction mixture and the crude was extracted with DCM (30 mL x 3). The combined organic extracts were then dried with anhydrous $MgSO_4$ and concentrated under vacuum. The residue was purified by flash column chromatography on silica gel to give the corresponding products.

4. General Procedure for Carbonylation of 1,3-Butadiene

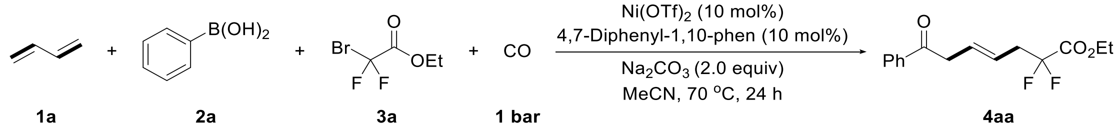

A 4 mL screw-capped vial was charged with $Ni(OTf)_2$ (10 mol%, 0.02mmol, 7.1mg), **L1** (10 mol%, 0.02 mmol, 6.6 mg), arylboronic acid (**2a**, 1.0 eq, 0.2 mmol), Na_2CO_3 (42.4 mg, 2.0 eq, 0.4 mmol) and an oven-dried stirring bar. The vial was sealed with a Teflon septum and cap and connected to the atmosphere via a needle. After adding 1,3-Butadiene (**1a**, 2.0 eq, 0.4 mmol, 2 mol/L in THF), $BrCF_2COOEt$ (**3a**, 1.5 eq, 0.3 mmol) and MeCN (1.0 mL) with a syringe under nitrogen atmosphere, the vial was moved to an alloy plate and placed in a Parr 4560 series autoclave (300 mL). At room temperature, the autoclave was purged three times with CO and pressurized with 1 bar of CO and 5 bar of N_2. The autoclave was placed on a heating plate equipped with a magnetic stirrer and an aluminum block. The reaction mixture was heated to 70 °C for 24 h. After the reaction was completed, the autoclave was cooled to room temperature with ice water and the pressure was carefully released. After cooling to room temperature, the reaction mixture was diluted with EA (~20 mL) and filtered through a celite pad. The filtrate was concentrated and the residue was purified by flash column chromatography to give **4aa**.

2 mmol scale: A 25 mL screw-cap vial was charged with $Ni(OTf)_2$ (10 mol%, 0.2mmol, 71.4mg,), **L2** (10 mol%, 0.2 mmol, 66.4 mg), arylboronic acid (**2a**, 1.0 eq, 2 mmol), Na_2CO_3 (424 mg, 2.0 eq, 4 mmol) and an oven-dried stirring bar. The vial was closed with a Teflon septum and cap and connected to the atmosphere via a needle. After adding 1,3-Butadiene (**1a**, 2.0 eq, 4 mmol, 2 mol/L in THF), $BrCF_2COOEt$ (**3a**, 1.5 eq, 3 mmol) and MeCN (10.0 mL) with a syringe under nitrogen atmosphere, the vial was moved to an alloy plate and placed in a Parr 4560 series autoclave (300 mL). At room temperature, the autoclave was purged three times with CO and pressurized with 1

bar of CO and 5 bar of N_2. The autoclave was placed on a heating plate equipped with a magnetic stirrer and an aluminum block. The reaction mixture was heated to 70 °C for 24 h. After the reaction mixture was cooled to room temperature, diluted with EA (30 mL) and filtered through a celite pad. The filtrate was concentrated and the residue was then purified by column chromatography on silica gel using petroleum and ethyl acetate (PE:EA = 30:1) to give **4aa** (373.3 mg, 66%).

5. Derivatization Reactions

a) General procedure for **5aa**[2]:

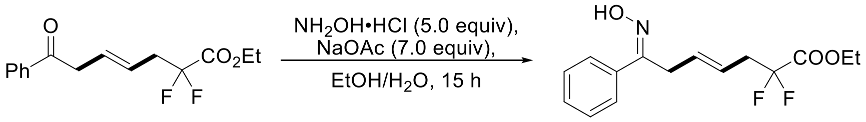

4aa **5aa**, 52%, Z/E = 1/4

A round-bottom flask was charged with a solution of hydroxylamine hydrochloride (69.5 mg, 1.0 mmol, 5.0 equiv) in ethanol (0.6 mL). Sodium acetate (190.5 mg, 1.4 mmol, 7.0 equiv) was dissolved in water (0.6 mL) and the solution was added to the flask. The **4aa** (56.4 mg, 0.2 mmol, 1.0 equiv) was dissolved in ethanol (0.6 mL) and added to the solution. The resulting suspension was stirred for 15 h at room temperature. The reaction was concentrated in vacuo and extracted with ethyl acetate (3 x 5 mL). The organic layers were dried over Na_2SO_4. The solvent was removed under vacuum and purified by silica gel chromatography (gradient of 15:1 PE: EA) to afford **5aa** as a colorless oil with 52% yield.

b) General procedure for **5ab**[3]:

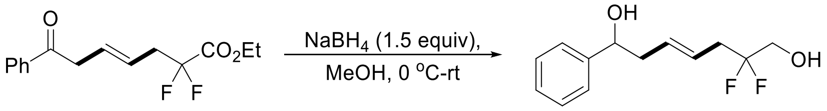

4aa **5ab**, 70% ,Z/E = 1/1

A methanolic solution (1 mL) of the **4aa** (56.4 mg, 0.2 mmol, 1.0 equiv) was cooled to 0 °C, charged with $NaBH_4$ (11.3 mg, 0.3 mmol, 1.5 equiv) portion wise, and allowed to warm to rt over 30 min. The reaction mixture was quenched with 2M HCl, concentrated in vacuo to a slurry and alkalized to pH 8 with sat. $NaHCO_3$ (2 mL). The aqueous layer was extracted with CH_2Cl_2 (3×10 mL) and the combined organic layers were dried over Na_2SO_4, filtered, and concentrated in vacuo. The resulting residue was purified by silica gel chromatography (gradient of 3:1 PE: EA) to afford **5ab** as a colorless oil with 70% yield.

c) General procedure for **5ac**[4]:

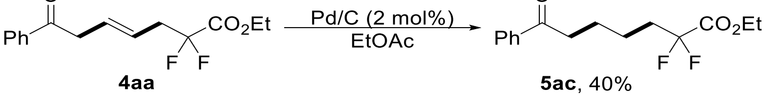

4aa **5ac**, 40%

To a 4 mL screw-capped vial were added Pd/C (8.5 mg, 0.004 mmol, 2 mol%), **4aa** (56.4 mg, 0.2 mmol, 1.0 equiv) and an oven-dried stirring bar. After adding EtOAc (2.0 mL) with a syringe under nitrogen atmosphere, the vial was moved to an alloy plate and placed in a Parr 4560 series autoclave (300 mL). The autoclave was purged three times with CO and pressurized with 5 bar of H_2. The autoclave was placed on a heating plate equipped with a magnetic stirrer and an aluminum

block. The reaction mixture was heated to 40 °C for 72 h. The resulting mixture was filtered through a short column of silica gel, eluted with ethyl acetate (5 mL x 3). The solvent was removed under vacuum and purified by silica gel chromatography (gradient of 20:1 PE: EA) to afford **5ac** as a colorless oil with 81% yield.

6. Mechanistic Studies

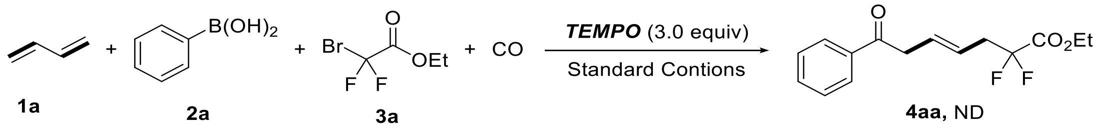

A 4 mL screw-capped vial was charged with $Ni(OTf)_2$ (10 mol%, 0.02mmol, 7.1mg,), **L2** (10 mol%, 0.02 mmol, 6.6 mg), arylboronic acid (**2a**, 1.0 eq, 0.2 mmol), Na_2CO_3 (42.4 mg, 2.0 eq, 0.4 mmol), Tempo (93.6 mg,3.0 equiv, 0.6 mmol) and an oven-dried stirring bar. The vial was closed with a Teflon septum and cap and connected to the atmosphere via a needle. After adding 1,3-butadiene (**1a**, 2.0 eq, 0.4 mmol), ethyl bromodifluoroacetate (**3a**, 1.5 eq, 0.3 mmol), and MeCN (1.0 mL) with a syringe under a nitrogen atmosphere, the vial was moved to an alloy plate and placed in a Parr 4560 series autoclave (300 mL) under a nitrogen atmosphere. At room temperature, the autoclave was purged three times with CO and pressurized with 1 bar of CO and 1 bar of N_2. The autoclave was placed on a heating plate equipped with a magnetic stirrer and an aluminum block. The reaction mixture was heated to 70 °C for 24 h. After the reaction was completed, the autoclave was cooled down with ice water to room temperature and the pressure was carefully released. After cooling to room temperature, no product **4aa** could be detected by GC-MS analysis.

7. Spectroscopic Data of Products

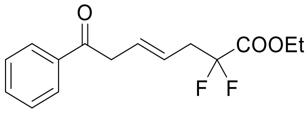

Ethyl (*E*)-2,2-difluoro-7-oxo-7-phenylhept-4-enoate (4aa):

Colorless oil, 40.6 mg, 72 % yield, Z/E = 1/4, R_f = 0.3 (PE/EA = 20/1).

^{1}H NMR (400 MHz, $CDCl_3$) δ 7.98 – 7.90 (m, 2H), 7.62 – 7.53 (m, 1H), 7.51 – 7.42 (m, 2H), 6.03 – 5.89 (m, 1H), 5.65 – 5.43 (m, 1H), 4.30 (q, *J* = 7.0 Hz, 2H), 3.76 (d, *J* = 5.3 Hz, 2H), 2.86 (td, *J* = 16.0, 7.2 Hz, 2H), 1.32 (t, *J* = 7.2 Hz, 3H).

^{13}C NMR (100 MHz, $CDCl_3$) δ 197.5, 163.8 (t, *J* = 32.5 Hz), 136.4, 133.3, 130.2, 128.7, 128.2, 122.7 (t, *J* = 5.5 Hz), 115.3 (t, *J* = 255.5 Hz), 62.9, 42.2, 38.2 (t, *J* = 24.1 Hz) 14.0.

^{19}F NMR (376 MHz, $CDCl_3$) δ -105.48.

HRMS (ESI-TOF) m/z: $[M + H]^+$ calculated for $C_{15}H_{17}F_2O_3$ 283.1140; Found 283.1136.

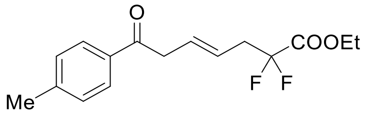

Ethyl (*E*)-2,2-difluoro-7-oxo-7-(*p*-tolyl)hept-4-enoate (4ab):

Colorless oil, 45.6 mg, 77 % yield, Z/E = 1/4, R_f = 0.3 (PE/EA = 20/1).

^{1}H NMR (400 MHz, $CDCl_3$) δ 7.87 – 7.80 (m, 2H), 7.30 – 7.23 (m, 2H), 6.04 – 5.88 (m, 1H), 5.63 – 5.48 (m, 1H), 4.29 (q, *J* = 7.1 Hz, 2H), 3.72 (d, *J* = 6.7 Hz, 2H), 2.86 (td, *J* = 16.0, 7.3 Hz, 2H), 2.41 (s, 3H), 1.32 (t, *J* = 7.1 Hz, 3H).

^{13}C NMR (100 MHz, $CDCl_3$) δ 197.1, 163.8 (t, *J* = 32.8 Hz), 144.1, 134.0, 130.4, 129.4, 128.4, 122.5 (t, *J* = 5.4 Hz), 115.3 (t, *J* = 248.8 Hz), 62.9, 42.1, 38.2 (t, *J* = 24.0 Hz), 21.7, 14.0.

^{19}F NMR (376 MHz, $CDCl_3$) δ -105.48.

HRMS (ESI-TOF) m/z: $[M + H]^+$ calculated for $C_{16}H_{19}F_2O_3$ 297.1297; Found 297.1301.

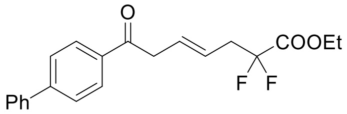

Ethyl (*E*)-7-([1,1'-biphenyl]-4-yl)-2,2-difluoro-7-oxohept-4-enoate (4ac):

While solid, 38.4 mg, 54 % yield, Z/E = 1/4, R_f = 0.25 (PE/EA = 20/1).

^{1}H NMR (400 MHz, $CDCl_3$) δ 8.03 – 7.97 (m, 2H), 7.74 – 7.67 (m, 2H), 7.66 – 7.59 (m, 2H), 7.52 – 7.45 (m, 2H), 7.45 – 7.36 (m, 1H), 6.05 – 5.93 (m, 1H), 5.64 – 5.54 (m, 1H), 4.30 (q, *J* = 7.1 Hz, 2H), 3.79 (d, *J* = 6.7 Hz, 2H), 2.88 (td, *J* = 16.1, 7.2 Hz, 2H), 1.33 (t, *J* = 7.2 Hz, 3H).

^{13}C NMR (100 MHz, $CDCl_3$) δ 197.1, 163.8 (t, *J* = 32.3 Hz), 146.0, 139.8, 135.1, 130.2, 129.0, 128.8, 128.3, 127.33, 127.28, 122.7 (t, *J* = 5.4 Hz), 115.3 (t, *J* = 251.5 Hz), 62.9, 42.2, 38.2 (t, *J* = 24.0 Hz), 14.0.

^{19}F NMR (376 MHz, $CDCl_3$) δ -105.43.

HRMS (ESI-TOF) m/z: $[M + H]^+$ calculated for $C_{21}H_{21}F_2O_3$ 359.1453; Found 359.1444.

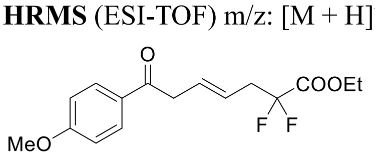

Ethyl (*E*)-2,2-difluoro-7-(4-methoxyphenyl)-7-oxohept-4-enoate (4ad):

Colorless oil, 48.4 mg, 76 % yield, Z/E = 1/3, R_f = 0.3 (PE/EA = 10/1).

^{1}H NMR (400 MHz, $CDCl_3$) δ 7.94 – 7.89 (m, 2H), 6.96 – 6.90 (m, 2H), 6.01 – 5.89 (m, 1H), 5.59 – 5.47 (m, 1H), 4.29 (q, *J* = 7.1 Hz, 2H), 3.86 (s, 3H), 3.70 (d, *J* = 6.8 Hz, 2H), 2.85 (td, *J* = 16.0, 7.2 Hz, 2H), 1.31 (t, *J* = 7.1 Hz, 3H).
^{13}C NMR (100 MHz, $CDCl_3$) δ 196.0, 163.7 (t, *J* = 16.2 Hz), 130.6, 130.5, 129.5, 122.3 (t, *J* = 5.5 Hz), 115.3 (t, *J* = 251.2 Hz), 113.8, 62.9, 55.5, 41.9, 38.2 (t, *J* = 24.0 Hz), 14.0.
^{19}F NMR (376 MHz, $CDCl_3$) δ -105.49.
HRMS (ESI-TOF) m/z: $[M + H]^+$ calculated for $C_{16}H_{19}F_2O_4$ 313.1246; Found 313.1240.

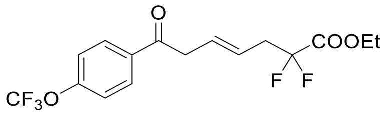

Ethyl (*E*)-2,2-difluoro-7-oxo-7-(4-(trifluoromethoxy)phenyl)hept-4-enoate (4ae):
Colorless oil, 51.7 mg, 71 % yield, Z/E = 1/5, R_f = 0.25 (PE/EA = 20/1).
^{1}H NMR (400 MHz, $CDCl_3$) δ 8.01 – 7.96 (m, 2H), 7.29 (d, *J* = 7.9 Hz, 2H), 6.03 – 5.86 (m, 1H), 5.71 – 5.45 (m, 1H), 4.30 (q, *J* = 7.2 Hz, 2H), 3.74 (d, *J* = 8.1 Hz, 2H), 2.86 (td, *J* = 16.0, 7.2 Hz, 2H), 1.32 (t, *J* = 7.1 Hz, 3H).
^{13}C NMR (100 MHz, $CDCl_3$) δ 195.9, 163.8 (t, *J* = 32.4 Hz), 152.8 (d, *J* = 1.9 Hz), 134.6, 130.3, 129.7, 123.0, 120.5, 115.2 (t, *J* = 251.4 Hz), 62.9, 42.2, 38.2 (t, *J* = 24.0 Hz), 14.0.
^{19}F NMR (376 MHz, $CDCl_3$) δ -57.62, -105.54.
HRMS (ESI-TOF) m/z: $[M + H]^+$ calculated for $C_{16}H_{16}F_5O_4$ 367.0963; Found 367.0960.

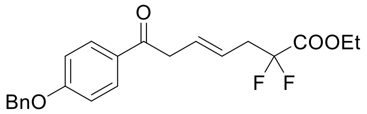

Ethyl (*E*)-7-(4-(benzyloxy)phenyl)-2,2-difluoro-7-oxohept-4-enoate (4af):
Yellow solide, 38.0 mg, 49 % yield, Z/E = 1/4, R_f = 0.25 (PE/EA = 10/1).
^{1}H NMR (400 MHz, $CDCl_3$) δ 8.09 – 7.66 (m, 2H), 7.48 – 7.31 (m, 5H), 7.01 (d, *J* = 8.9 Hz, 2H), 6.10 – 5.85 (m, 1H), 5.62 – 5.46 (m, 1H), 5.14 (s, 2H), 4.29 (q, *J* = 7.2 Hz, 2H), 3.70 (d, *J* = 6.8 Hz, 2H), 2.86 (td, *J* = 15.8, 7.3 Hz, 2H), 1.32 (t, *J* = 7.1 Hz, 3H).
^{13}C NMR (100 MHz, $CDCl_3$) δ 196.0, 163.7 (t, *J* = 32.5 Hz), 162.8, 136.1, 130.6, 129.7, 128.7, 128.3, 127.5, 122.4 (t, *J* = 5.5 Hz), 115.3 (t, *J* = 251.2 Hz), 114.7, 70.2, 62.9, 41.9, 38.2 (t, *J* = 24.0 Hz), 14.0.
^{19}F NMR (376 MHz, $CDCl_3$) δ -105.47.
HRMS (ESI-TOF) m/z: $[M + H]^+$ calculated for $C_{22}H_{23}F_2O_4$ 389.1559; Found 389.1559.

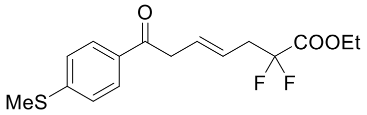

Ethyl (*E*)-2,2-difluoro-7-(4-(methylthio)phenyl)-7-oxohept-4-enoate (4ag):
Colorless oil, 51.5 mg, 79 % yield, Z/E = 1/4, R_f = 0.25 (PE/EA = 15/1).
^{1}H NMR (400 MHz, $CDCl_3$) δ 7.87 – 7.83 (m, 2H), 7.28 – 7.25 (m, 2H), 6.02 – 5.90 (m, 1H), 5.60 – 5.50 (m, 1H), 4.30 (q, *J* = 7.1 Hz, 2H), 3.71 (d, *J* = 6.7 Hz, 2H), 2.86 (td, *J* = 15.9, 7.1 Hz, 2H), 2.52 (s, 3H), 1.32 (t, *J* = 7.1 Hz, 3H).
^{13}C NMR (100 MHz, $CDCl_3$) δ 196.4, 163.8 (t, *J* = 32.7 Hz), 146.2, 132.7, 130.3, 128.6, 125.0, 122.6 (t, *J* = 5.5 Hz), 115.3 (t, *J* = 251.3 Hz), 62.9, 42.0, 38.2 (t, *J* = 24.0 Hz), 14.7, 14.0.
^{19}F NMR (376 MHz, $CDCl_3$) δ -105.47.
HRMS (ESI-TOF) m/z: $[M + H]^+$ calculated for $C_{16}H_{19}F_2O_3S$ 329.1017; Found 329.1022.

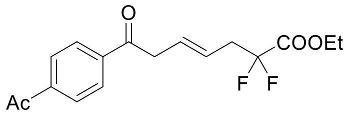

Ethyl (*E*)-7-(4-acetylphenyl)-2,2-difluoro-7-oxohept-4-enoate (4ah):

Colorless oil, 31,7 mg, 49 % yield, Z/E = 1/4, R_f = 0.25 (PE/EA = 20/1)

^{1}H NMR (400 MHz, $CDCl_3$) δ 8.06 – 8.04 (m, 1H), 8.03 – 8.01 (m, 3H), 6.01 – 5.90 (m, 1H), 5.63 – 5.52 (m, 1H), 4.30 (t, *J* = 7.2 Hz, 2H), 3.79 (d, *J* = 6.7 Hz, 2H), 2.91 – 2.81 (m, 2H), 2.64 (s, 3H), 1.33 (t, *J* = 7.2 Hz, 3H).

^{13}C NMR (100 MHz, $CDCl_3$) δ 197.4, 196.9, 163.8 (t, *J* = 32.2 Hz), 140.3, 139.5, 129.5, 128.6, 128.4, 123.1 (t, *J* = 5.5 Hz), 115.2, 62.9, 42.5, 38.2 (t, *J* = 24.0 Hz), 26.9, 14.0.

^{19}F NMR (376 MHz, $CDCl_3$) δ -104.91.

HRMS (ESI-TOF) m/z: $[M + H]^+$ calculated for $C_{17}H_{19}F_2O_4$ 325.1246; Found 325.1250.

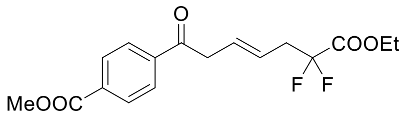

Ethyl (*E*)-7-(4-acetoxyphenyl)-2,2-difluoro-7-oxohept-4-enoate (4ai):

Colorless oil, 50.0 mg, 74 % yield, Z/E = 1/4, R_f = 0.25 (PE/EA = 10/1)

^{1}H NMR (400 MHz, $CDCl_3$) δ 8.14 – 8.11 (m, 2H), 8.01 – 7.97 (m, 2H), 6.01 – 5.90 (m, 1H), 5.62 – 5.52 (m, 1H), 4.31 (q, *J* = 7.2 Hz, 2H), 3.95 (s, 3H), 3.79 (d, *J* = 6.8 Hz, 2H), 2.88 (dt, *J* = 16.0, 7.7 Hz, 2H), 1.33 (t, *J* = 7.2 Hz, 3H).

^{13}C NMR (100 MHz, $CDCl_3$) δ 197.0, 166.2, 163.8, 139.6, 134.1, 129.9, 129.6, 128.1, 123.1 (t, *J* = 55.5 Hz), 115.2, 62.9, 52.5, 42.4, 38.2 (t, *J* = 238.4 Hz), 14.0.

^{19}F NMR (376 MHz, $CDCl_3$) -105.52.

HRMS (ESI-TOF) m/z: $[M + H]^+$ calculated for $C_{17}H_{19}F_2O_5$ 341.1195; Found 341.1204.

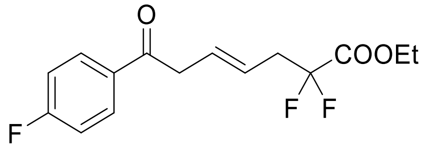

Ethyl (*E*)-2,2-difluoro-7-(4-fluorophenyl)-7-oxohept-4-enoate (4aj):

Colorless oil, 26.0 mg, 43 % yield, Z/E = 1/3, R_f = 0.3 (PE/EA = 20/1).

^{1}H NMR (400 MHz, $CDCl_3$) δ 8.00 – 7.94 (m, 2H), 7.17 – 7.11 (m, 2H), 5.99 – 5.92 (m, 1H), 5.60 – 5.53 (m, 1H), 4.30 (q, *J* = 7.2 Hz, 2H), 3.73 (d, *J* = 6.7 Hz, 2H), 2.86 (td, *J* = 16.1, 15.4, 7.3 Hz, 2H), 1.33 (t, *J* = 7.1 Hz, 3H).

^{13}C NMR (100 MHz, $CDCl_3$) δ 195.8, 165.8 (d, *J* = 255.0 Hz), 163.8 (t, *J* = 32.7 Hz), 132.8 (d, *J* = 3.0 Hz), 130.9, 130.8, 129.9, 122.8 (t, *J* = 5.5 Hz), 115.9, 115.7, 115.2 (t, *J* = 251.3 Hz), 62.9, 42.1, 38.2 (t, *J* = 24.1 Hz), 14.0.

^{19}F NMR (376 MHz, $CDCl_3$) δ -104.82, -105.52.

HRMS (ESI-TOF) m/z: $[M + H]^+$ calculated for $C_{15}H_{16}F_3O_3$ 301.1046 ; Found 301.1052.

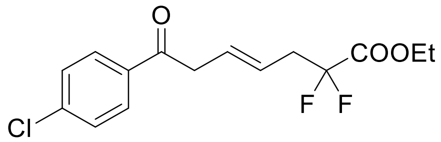

Ethyl (*E*)-7-(4-chlorophenyl)-2,2-difluoro-7-oxohept-4-enoate (4ak):

Colorless oil, 31.6 mg, 50 % yield, Z/E = 1/3, R_f = 0.3 (PE/EA = 20/1).

¹H NMR (400 MHz, $CDCl_3$) δ 7.88 – 7.86 (m, 2H), 7.45 – 7.42 (m, 2H), 5.97 – 5.90 (m, 1H), 5.58 – 5.53 (m, 1H), 4.30 (q, *J* = 7.1 Hz, 2H), 3.72 (d, *J* = 7.5 Hz, 2H), 2.85 (td, *J* = 15.6, 7.4 Hz, 2H), 1.32 (t, *J* = 7.1 Hz, 3H)

¹³C NMR (100 MHz, $CDCl_3$) δ 196.2, 163.8 (t, *J* = 32.6 Hz), 139.8, 134.7, 129.8, 129.6, 129.0, 122.9 (t, *J* = 5.5 Hz), 115.2 (t, *J* = 251.3 Hz), 62.9, 42.1, 38.2 (t, *J* = 24.1 Hz), 14.0.

¹⁹F NMR (376 MHz, $CDCl_3$) δ -105.51.

HRMS (ESI-TOF) m/z: $[M + H]^+$ calculated for $C_{15}H_{16}ClF_2O_3$ 317.0751; Found 317.0758.

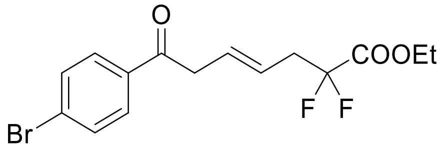

Ethyl (*E*)-7-(4-bromophenyl)-2,2-difluoro-7-oxohept-4-enoate (4al):

Colorless oil, 17.4 mg, 24 % yield, Z/E = 1/3, R_f = 0.3 (PE/EA = 20/1).

¹H NMR (400 MHz, $CDCl_3$) δ 7.82 – 7.79 (m, 2H), 7.63 – 7.60 (m, 2H), 5.99 – 5.89 (m, 1H), 5.61 – 5.51 (m, 1H), 4.31 (q, *J* = 7.1 Hz, 2H), 3.72 (d, *J* = 6.7 Hz, 2H), 2.88 (dt, *J* = 16.1, 7.9 Hz, 2H), 1.33 (t, *J* = 7.2 Hz, 3H).

¹³C NMR (100 MHz, $CDCl_3$) δ 196.4, 163.8 (t, *J* = 32.5 Hz), 135.1, 132.0, 129.7, 128.5, 123.0 (t, *J* = 5.5 Hz), 115.2 (t, *J* = 251.6 Hz), 62.9, 42.1, 38.2 (t, *J* = 24.1 Hz), 14.0.

¹⁹F NMR (376 MHz, $CDCl_3$) δ -105.50.

HRMS (ESI-TOF) m/z: $[M + H]^+$ calculated for $C_{15}H_{16}BrF_2O_3$ 361.0245; Found 361.0250.

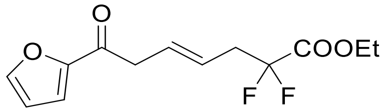

Ethyl (*E*)-7-(4-bromophenyl)-2,2-difluoro-7-oxohept-4-enoate (4am):

Colorless oil, 16.9 mg, 31% yield, Z/E = 1/5, R_f = 0.25 (PE/EA = 10/1).

¹H NMR (400 MHz, $CDCl_3$) δ 7.59 (d, *J* = 1.7 Hz, 1H), 7.20 (d, *J* = 3.6 Hz, 1H), 6.54 (dd, *J* = 3.6, 1.7 Hz, 1H), 5.97 – 5.85 (m, 1H), 5.63 – 5.51 (m, 1H), 4.30 (q, *J* = 7.2 Hz, 2H), 3.60 (d, *J* = 7.0 Hz, 2H), 2.84 (td, *J* = 16.0, 15.6, 7.2 Hz, 2H), 1.32 (t, *J* = 7.2 Hz, 3H).

¹³C NMR (100 MHz, $CDCl_3$) δ 186.4, 163.8 (t, *J* = 32.5 Hz), 152.2, 146.6, 129.4, 123.0 (t, *J* = 5.5 Hz), 117.6, 115.3, 112.4, 62.9, 42.1, 38.2 (t, *J* = 24.0 Hz), 14.0.

¹⁹F NMR (376 MHz, $CDCl_3$) δ -105.50.

HRMS (ESI-TOF) m/z: $[M + H]^+$ calculated for $C_{13}H_{15}F_2O_4$ 273.0933; Found 273.0933.

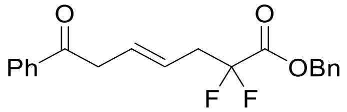

Benzyl (*E*)-2,2-difluoro-7-oxo-7-phenylhept-4-enoate (4ba):

Colorless oil, 54.4 mg, 79% yield, Z/E = 1/4, R_f = 0.3 (PE/EA = 20/1).

¹H NMR (400 MHz, $CDCl_3$) δ 7.94 – 7.91 (m, 2H), 7.60 – 7.56 (m, 1H), 7.49 – 7.45 (m, 2H), 7.39 – 7.35 (m, 5H), 5.93 – 5.83 (m, 1H), 5.56 – 5.44 (m, 1H), 5.26 (s, 2H), 3.66 (d, *J* = 6.7 Hz, 2H), 2.86 (td, *J* = 15.8, 7.2 Hz, 2H).

¹³C NMR (100 MHz, $CDCl_3$) δ 197.4, 163.7 (t, *J* = 32.8 Hz), 136.4, 134.4, 133.3, 130.4, 128.9, 128.74, 128.70, 128.67, 128.2, 122.4 (t, *J* = 5.7 Hz), 115.4 (t, *J* = 251.6 Hz), 68.3, 42.1, 38.3 (t, *J* = 23.9 Hz).

¹⁹F NMR (376 MHz, $CDCl_3$) δ -105.23.

HRMS (ESI-TOF) m/z: $[M + H]^+$ calculated for $C_{20}H_{19}F_2O_3$ 345.1297; Found 345.1307.

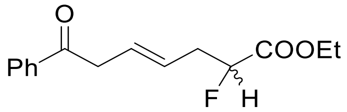

Ethyl (*E*)-2-fluoro-7-oxo-7-phenylhept-4-enoate (4bb):

Colorless oil, 22.3 mg, 42% yield, Z/E = 1/4, $R_f = 0.3$ (PE/EA = 10/1).

^{1}H NMR (400 MHz, $CDCl_3$) δ 7.96 – 7.93 (m, 2H), 7.59 – 7.55 (m, 1H), 7.49 – 7.45 (m, 2H), 5.95 – 5.85 (m, 1H), 5.68 – 5.59 (m, 1H), 5.00 – 4.87 (m, 1H), 4.23 (q, *J* = 7.2 Hz, 2H), 3.74 (d, *J* = 6.7 Hz, 2H), 2.83 – 2.56 (m, 2H), 1.28 (t, *J* = 7.1 Hz, 3H).

^{13}C NMR (100 MHz, $CDCl_3$) δ 197.9, 169.3 (d, *J* = 23.7 Hz), 136.5, 133.3, 128.7, 128.3, 127.6, 126.7 (d, *J* = 3.6 Hz), 88.3 (d, *J* = 185.7 Hz), 61.6, 42.2, 35.7 (d, *J* = 21.2 Hz), 14.2.

^{19}F NMR (376 MHz, $CDCl_3$) δ -191.68.

HRMS (ESI-TOF) m/z: $[M + H]^+$ calculated for $C_{15}H_{18}FO_3$ 265.1234; Found 265.1244.

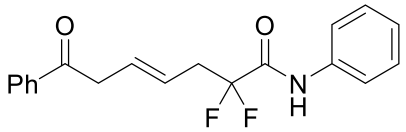

(*E*)-2,2-Difluoro-7-oxo-N,7-diphenylhept-4-enamide (4bc):

White solid, 23.7 mg, 36% yield, Z/E = 1/4, $R_f = 0.3$ (PE/EA = 10/1).

^{1}H NMR (400 MHz, $CDCl_3$) δ 7.98 (s, 1H), 7.95 – 7.90 (m, 2H), 7.57 – 7.53 (m, 3H), 7.47 – 7.41 (m, 2H), 7.38 – 7.34 (m, 2H), 7.21 – 7.17 (m, 1H), 6.06 – 5.97 (m, 1H), 5.64 – 5.57 (m, 1H), 3.76 (d, *J* = 6.9 Hz, 2H), 2.98 (td, *J* = 16.5, 7.1 Hz, 2H).

^{13}C NMR (100 MHz, $CDCl_3$) δ 197.6, 161.7 (t, *J* = 28.5 Hz), 136.4, 136.0, 133.3, 130.4, 129.2, 128.7, 128.2, 125.6, 122.9 (t, *J* = 5.3 Hz), 120.2, 117.0 (t, *J* = 254.7 Hz), 42.2, 37.3 (t, *J* = 24.2 Hz).

^{19}F NMR (376 MHz, $CDCl_3$) δ -105.31.

HRMS (ESI-TOF) m/z: $[M + H]^+$ calculated for $C_{19}H_{18}F_2NO_2$ 330.1300; Found 330.1299.

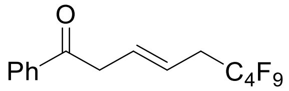

(*E*)-6,6,7,7,8,8,9,9,9-Nonafluoro-1-phenylnon-3-en-1-one (4bd):

Colorless oil, 37.5 mg, 50% yield, Z/E = 1/5, $R_f = 0.3$ (PE/EA = 50/1).

^{1}H NMR (400 MHz, $CDCl_3$) δ 7.99 – 7.95 (m, 2H), 7.60 – 7.56 (m, 1H), 7.50 – 7.45 (m, 2H), 6.10 – 6.00 (m, 1H), 5.66 – 5.56 (m, 1H), 3.81 (d, *J* = 6.8 Hz, 2H), 2.89 (td, *J* = 18.1, 17.6, 7.1 Hz, 2H).

^{13}C NMR (100 MHz, $CDCl_3$) δ 197.4, 136.4, 133.4, 131.0, 128.7, 128.2, 120.6, 42.1, 34.8 (t, *J* = 23.1 Hz).

^{19}F NMR (376 MHz, $CDCl_3$) δ -80.98 – -81.08 (m, 3F), -113.34 – -113.49 (m, 2F), -124.00 – -124.15 (m, 2F), -126.03 – -126.16 (m, 2F).

HRMS (ESI-TOF) m/z: $[M + H]^+$ calculated for $C_{15}H_{12}F_9O$ 379.0739; Found 379.0745.

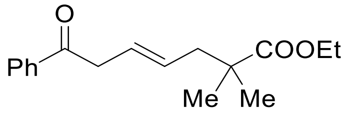

Ethyl (*E*)-2,2-dimethyl-7-oxo-7-phenylhept-4-enoate (4be):

Colorless oil, 20.6 mg, 38% yield, Z/E = 1/5, $R_f = 0.3$ (PE/EA = 20/1).

^{1}H NMR (400 MHz, $CDCl_3$) δ 7.98 – 7.93 (m, 2H), 7.58 – 7.52 (m, 1H), 7.48 – 7.43 (m, 2H), 5.78

– 5.67 (m, 1H), 5.59 – 5.48 (m, 1H), 4.08 (q, *J* = 7.1 Hz, 2H), 3.70 (d, *J* = 6.6 Hz, 2H), 2.28 (d, *J* = 6.2 Hz, 2H), 1.22 (t, *J* = 7.1 Hz, 3H), 1.14 (s, 6H).

^{13}C NMR (100 MHz, $CDCl_3$) δ 198.3, 177.5, 136.6, 133.1, 130.3, 128.6, 128.3, 125.9, 60.3, 43.5, 42.5, 42.4, 24.9, 14.3.

HRMS (ESI-TOF) m/z: $[M + Na]^+$ calculated for $C_{17}H_{22}O_3Na^+$ 297.1461; Found 297.1460.

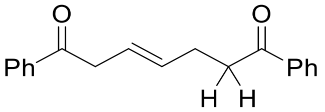

(*E*)-1,7-Diphenylhept-3-ene-1,7-dione (4bf):

Colorless oil, 15.0 mg, 27% yield, Z/E = 1/5, R_f = 0.25 (PE/EA = 10/1).

^{1}H NMR (400 MHz, $CDCl_3$) δ 7.97 – 7.94 (m, 3H), 7.57 – 7.53 (m, 2H), 7.49 – 7.41 (m, 5H), 5.81 – 5.69 (m, 2H), 3.71 (d, *J* = 7.3 Hz, 2H), 3.08 (t, *J* = 7.3 Hz, 2H), 2.52 (q, *J* = 6.6 Hz, 2H).

^{13}C NMR (100 MHz, $CDCl_3$) δ 199.5, 198.5, 136.9, 136.6, 133.2, 133.15, 133.1, 128.64, 128.61, 128.3, 128.1, 123.4, 42.4, 38.1, 27.1.

HRMS (ESI-TOF) m/z: $[M + H]^+$ calculated for $C_{19}H_{19}O_2$ 279.1380; Found 279.1385.

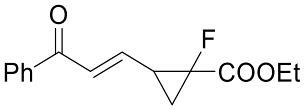

Ethyl (*E*)-1-fluoro-2-(3-oxo-3-phenylprop-1-en-1-yl)cyclopropane-1-carboxylate (4bg):

Colorless oil, 24.6 mg, 47% yield, Z/E = 1/1, R_f = 0.3 (PE/EA = 20/1).

^{1}H NMR (400 MHz, $CDCl_3$) δ 7.99 – 7.87 (m, 2H), 7.60 – 7.52 (m, 1H), 7.51 – 7.42 (m, 2H), 7.13 (d, *J* = 15.4 Hz, 1H), 6.82 – 6.71 (m, 1H), 4.30 (q, *J* = 7.1 Hz, 2H), 2.62 – 2.48 (m, 1H), 1.98 – 1.86 (m, 1H), 1.72 – 1.59 (m, 1H), 1.34 (t, *J* = 7.1 Hz, 3H).

^{13}C NMR (100 MHz, $CDCl_3$) δ 189.5, 169.0 (d, *J* = 23.2 Hz), 142.9 (d, *J* = 6.9 Hz), 137.5, 132.9, 128.7, 128.6, 127.9, 78.4 (d, *J* = 238.4 Hz), 62.3, 29.3 (d, *J* = 9.6 Hz), 21.8 (d, *J* = 10.3 Hz), 14.2.

^{19}F NMR (376 MHz, $CDCl_3$) δ -204.68.

HRMS (ESI-TOF) m/z: $[M + H]^+$ calculated for $C_{15}H_{16}FO_3$ 263.1078; Found 263.1073.

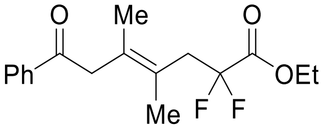

(*E*)-1,7-Diphenylhept-3-ene-1,7-dione (4ca):

Colorless oil, 22.7 mg, 37% yield, Z/E = 1/1, R_f = 0.25 (PE/EA = 20/1).

^{1}H NMR (400 MHz, $CDCl_3$) δ 7.99 – 7.94 (m, 2H), 7.60 – 7.55 (m, 1H), 7.50 – 7.44 (m, 2H), 4.34 – 4.27 (m, 2H), 3.81 (d, *J* = 9.5 Hz, 2H), 2.96 (t, *J* = 16.9 Hz, 1H), 2.85 (t, *J* = 17.1 Hz, 1H), 1.84 (s, 1H), 1.80 – 1.72 (m, 5H), 1.34 (q, *J* = 7.0 Hz, 3H).

^{13}C NMR (100 MHz, $CDCl_3$) δ 197.7, 197.5, 137.0, 133.2, 130.0, 128.7, 128.1, 122.6, 116.1, 62.9, 44.7, 39.3 (t, *J* = 23.1 Hz), 20.1, 19.9, 14.0.

HRMS (ESI-TOF) m/z: $[M + H]^+$ calculated for $C_{17}H_{21}F_2O_3$ 311.1453; Found 311.1451.

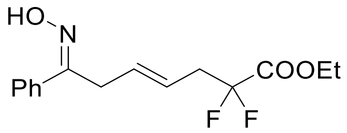

Ethyl (4*E*,7*Z*)-2,2-difluoro-7-(hydroxyimino)-7-phenylhept-4-enoate (5aa):
Colorless oil, 26.7 mg, 45% yield, Z/E = 1/4, R_f = 0.25 (PE/EA = 15/1).
^{1}H NMR (400 MHz, $CDCl_3$) δ 7.61 – 7.58 (m, 2H), 7.40 – 7.37 (m, 3H), 5.86 – 5.74 (m, 1H), 5.60 – 5.46 (m, 1H), 4.21 (q, *J* = 7.1 Hz, 2H), 3.57 (d, *J* = 6.4 Hz, 2H), 2.78 (td, *J* = 15.9, 7.2 Hz, 2H), 1.26 (d, *J* = 7.1 Hz, 3H).
^{13}C NMR (100 MHz, $CDCl_3$) δ 163.8 (t, *J* = 32.7 Hz), 156.5, 135.4, 131.4, 129.4, 128.6, 126.3, 121.3, 115.3 (t, *J* = 251.3 Hz), 62.8, 38.1 (t, *J* = 24.1 Hz), 30.1, 13.9.
^{19}F NMR (376 MHz, $CDCl_3$) δ -105.37.
HRMS (ESI-TOF) m/z: $[M + H]^+$ calculated for $C_{15}H_{18}F_2NO_3$ 298.1249; Found 298.1243.

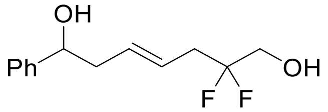

(*E*)-6,6-Difluoro-1-phenylhept-3-ene-1,7-diol (5ab):
Colorless oil, 33.9 mg, 70% yield, Z/E = 1/1, R_f = 0.25 (PE/EA = 3/1).
^{1}H NMR (400 MHz, DMSO-*d*$_6$) δ 7.36 – 7.28 (m, 4H), 7.24 – 7.18 (m, 1H), 5.65 – 5.55 (m, 1H), 5.42 (t, *J* = 6.3 Hz, 1H), 5.40 – 5.31 (m, 1H), 5.23 (d, *J* = 4.4 Hz, 1H), 4.58 – 4.52 (m, 1H), 3.48 (td, *J* = 13.6, 6.3 Hz, 2H), 2.65 – 2.51 (m, 2H), 2.42 – 2.27 (m, 2H).
^{13}C NMR (100 MHz, $CDCl_3$) δ 143.7, 132.1, 128.5, 127.7, 125.8, 124.4 (t, *J* = 5.9 Hz), 122.2, 73.5, 63.5 (t, *J* = 31.9 Hz), 42.4, 37.4 (t, *J* = 24.9 Hz).
^{19}F NMR (376 MHz, DMSO-*d*$_6$) δ -105.61.
HRMS (ESI-TOF) m/z: $[M + Na]^+$ calculated for $C_{13}H_{16}F_2O_2Na^+$ 265.1011; Found 265.1016.

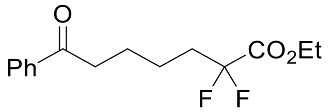

Ethyl 2,2-difluoro-7-oxo-7-phenylheptanoate (5ac):
Colorless oil, 22.6 mg, 40% yield, R_f = 0.3 (PE/EA = 20/1).
^{1}H NMR (400 MHz, $CDCl_3$) δ 7.92 – 7.83 (m, 2H), 7.52 – 7.45 (m, 1H), 7.38 (t, *J* = 7.6 Hz, 2H), 4.24 (q, *J* = 7.1 Hz, 2H), 2.92 (t, *J* = 7.2 Hz, 2H), 2.12 – 1.98 (m, 2H), 1.73 (p, *J* = 7.4 Hz, 2H), 1.55 – 1.46 (m, 2H), 1.27 (t, *J* = 7.1 Hz, 3H).
^{13}C NMR (100 MHz, $CDCl_3$) δ 199.5, 164.1 (t, *J* = 32.8 Hz), 136.9, 133.1, 128.6, 128.0, 116.2, 62.8, 38.0, 34.4 (t, *J* = 23.3 Hz), 23.5, 21.2 (t, *J* = 4.4 Hz), 14.0.
HRMS (ESI-TOF) m/z: $[M + H]^+$ calculated for $C_{15}H_{19}F_2O_3$ 285.1297; Found 285.1289.

8. NMR Spectra of the Products

NMR spectrums of **4aa**

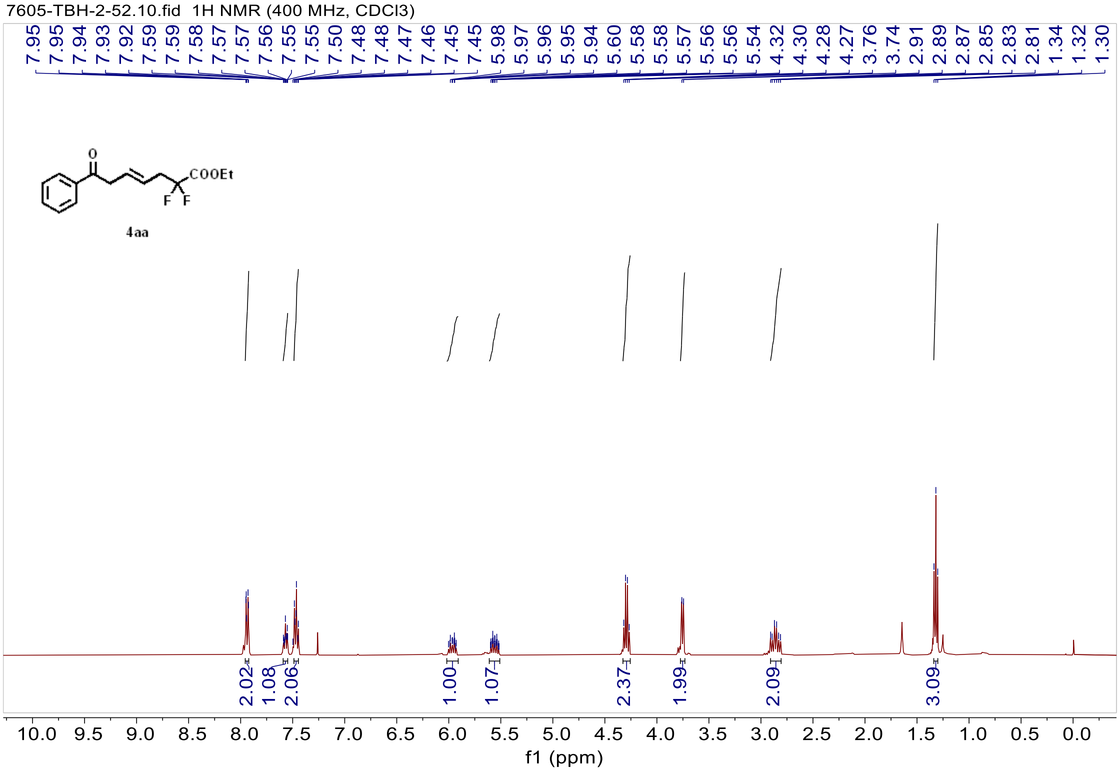

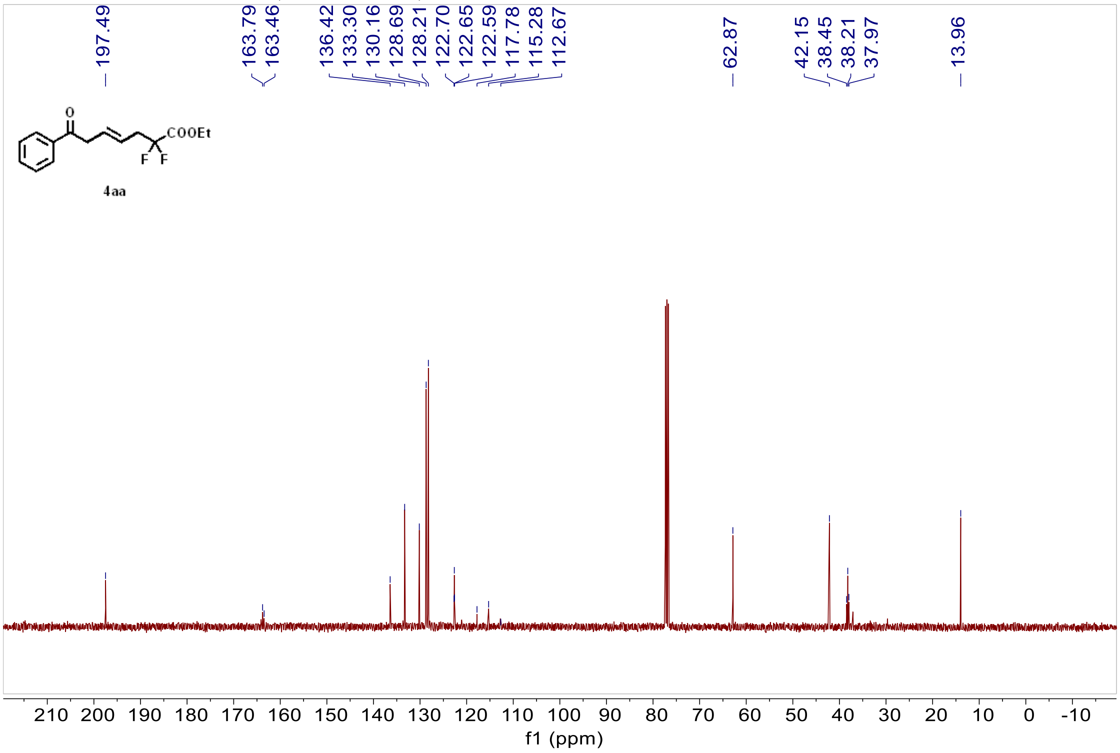

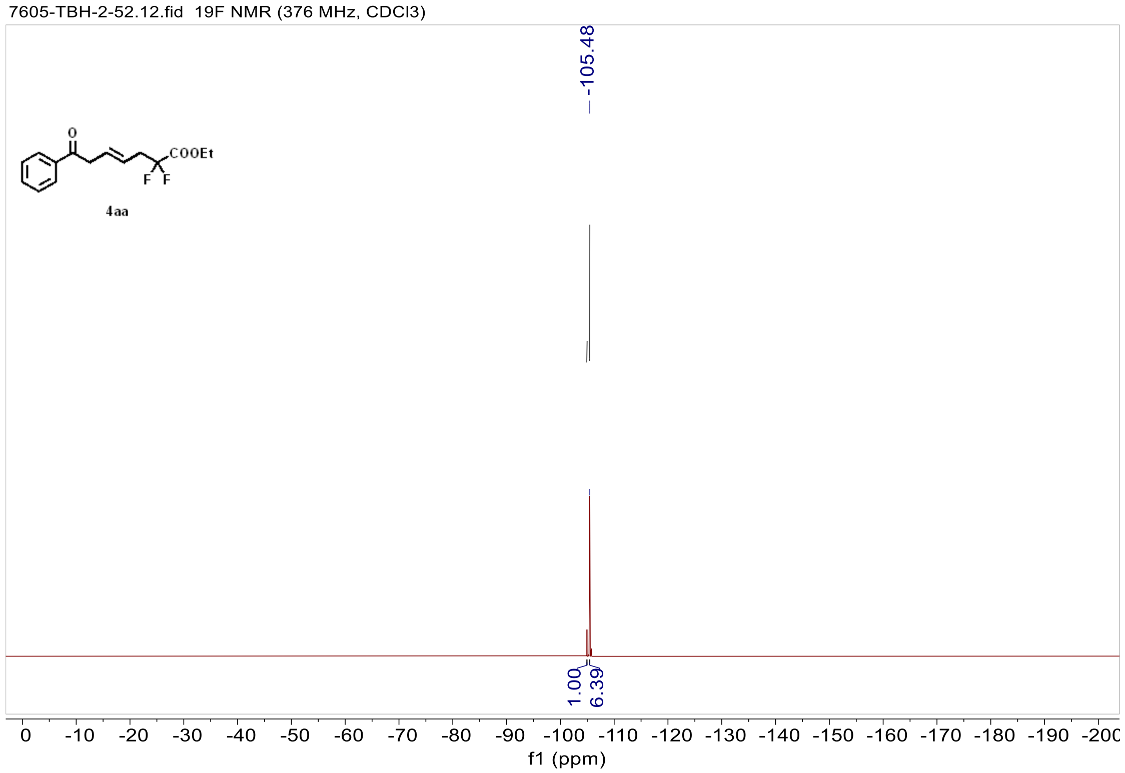
7605-TBH-2-52.12.fid 19F NMR (376 MHz, CDCl3)
-105.48
O
COOEt
F F
4aa
1.00
6.39
0
-10
-20
-30
-40
-50
-60
-70
-80
-90
-100
-110
-120
-130
-140
-150
-160
-170
-180
-190
-200
f1 (ppm)

NMR spectrums of **4ab**

9814-TBH-2-81-1.10.fid 1H NMR (400 MHz, CDCl3)

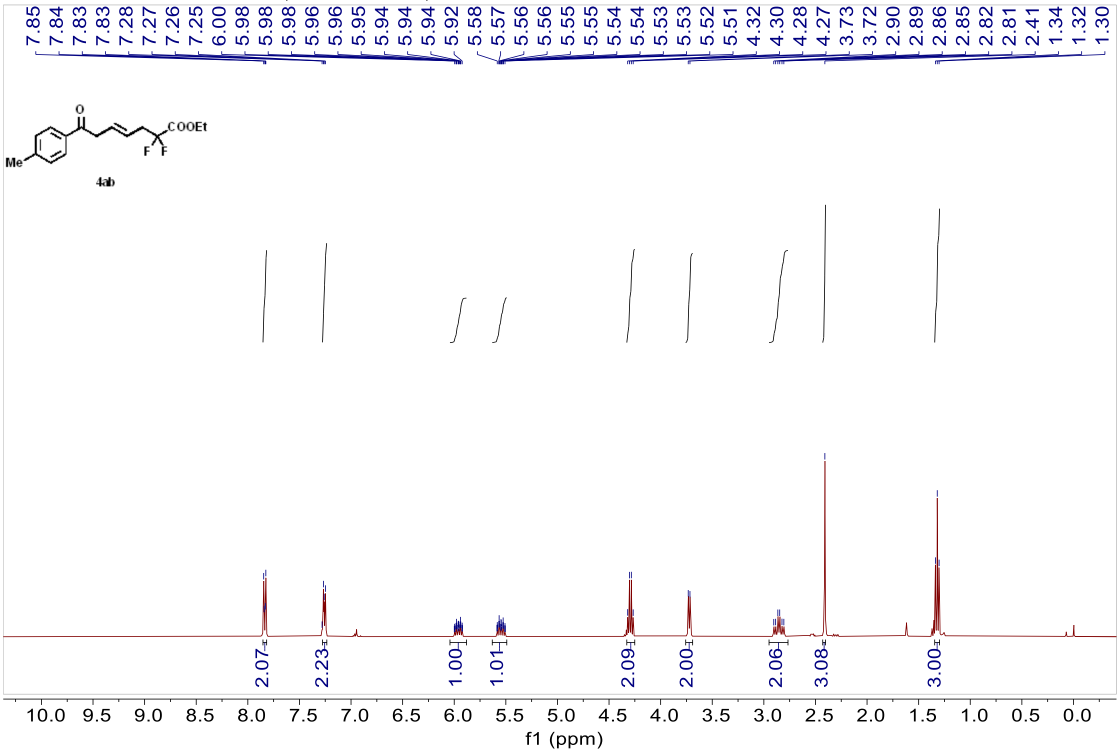

9814-TBH-2-81-1.11.fid 13C NMR (100 MHz, CDCl3)

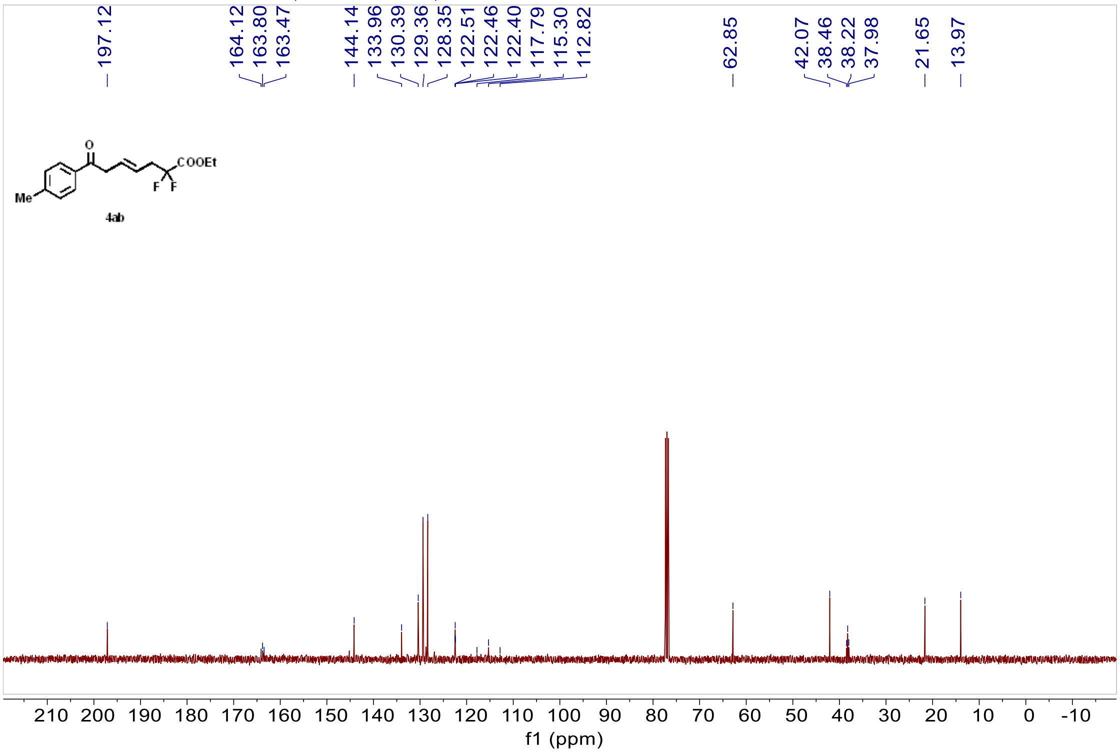

9814-TBH-2-81-1.12.fid 19F NMR (376 MHz, CDCl3)

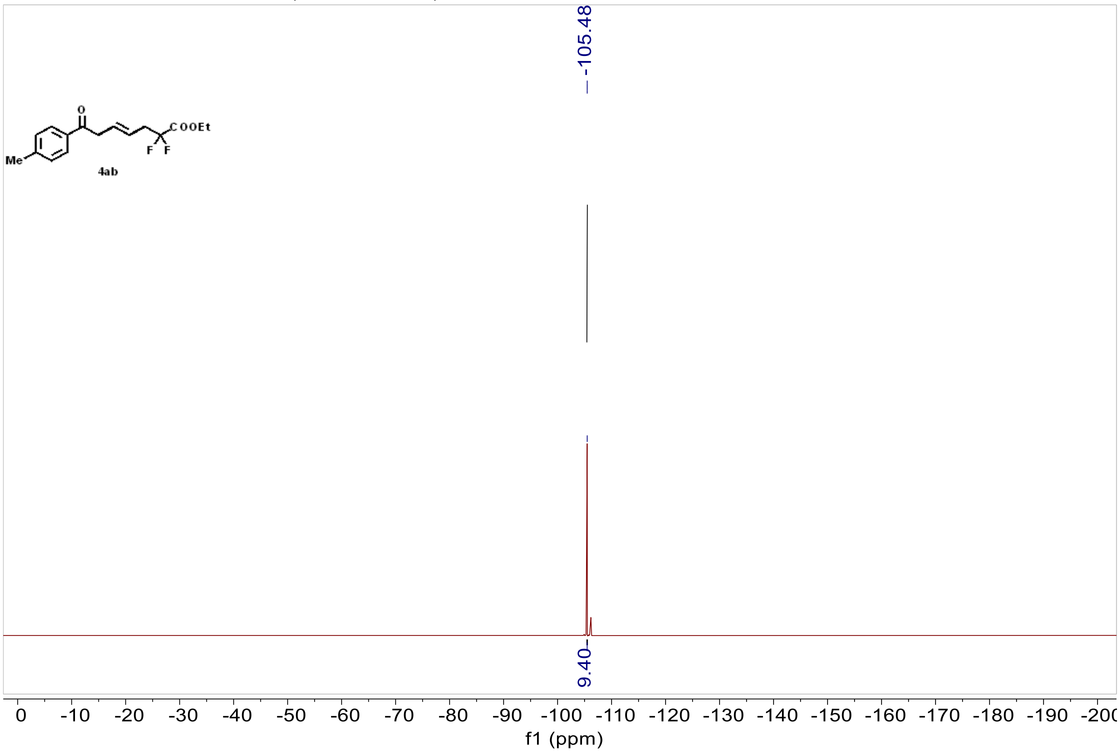

NMR spectrums of **4ac**

9907-TBH-2-81-7.10.fid 1H NMR (400 MHz, CDCl3)

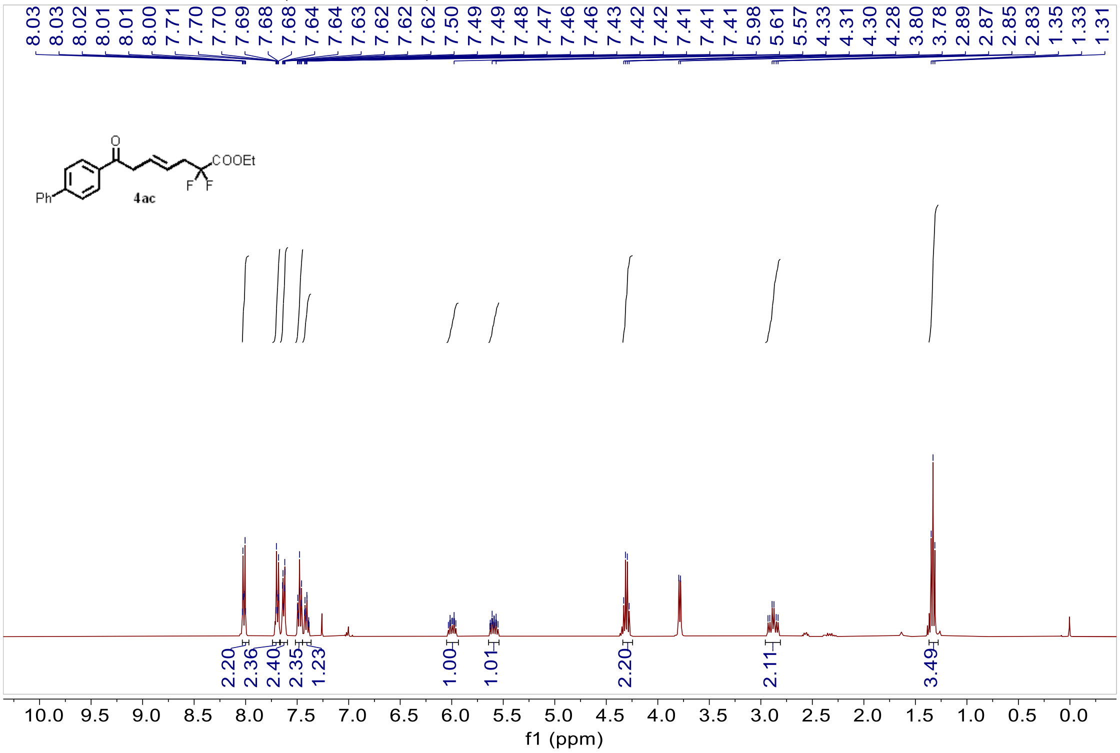

9907-TBH-2-81-7.11.fid 13C NMR (100 MHz, CDCl3)

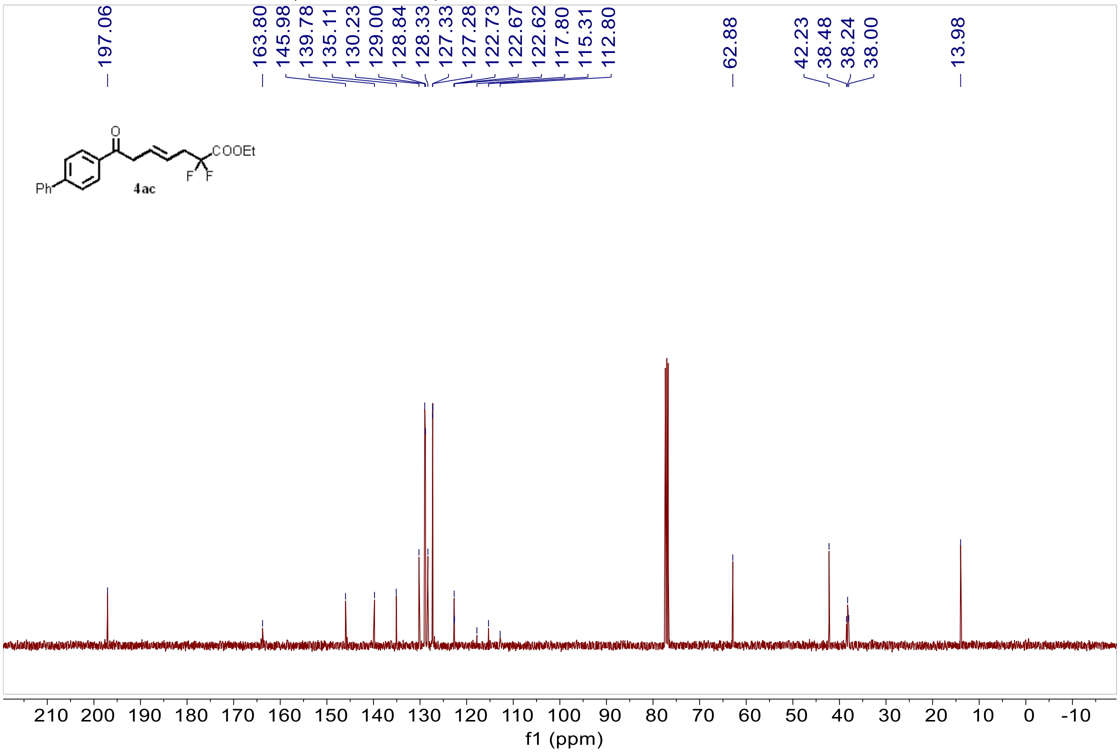

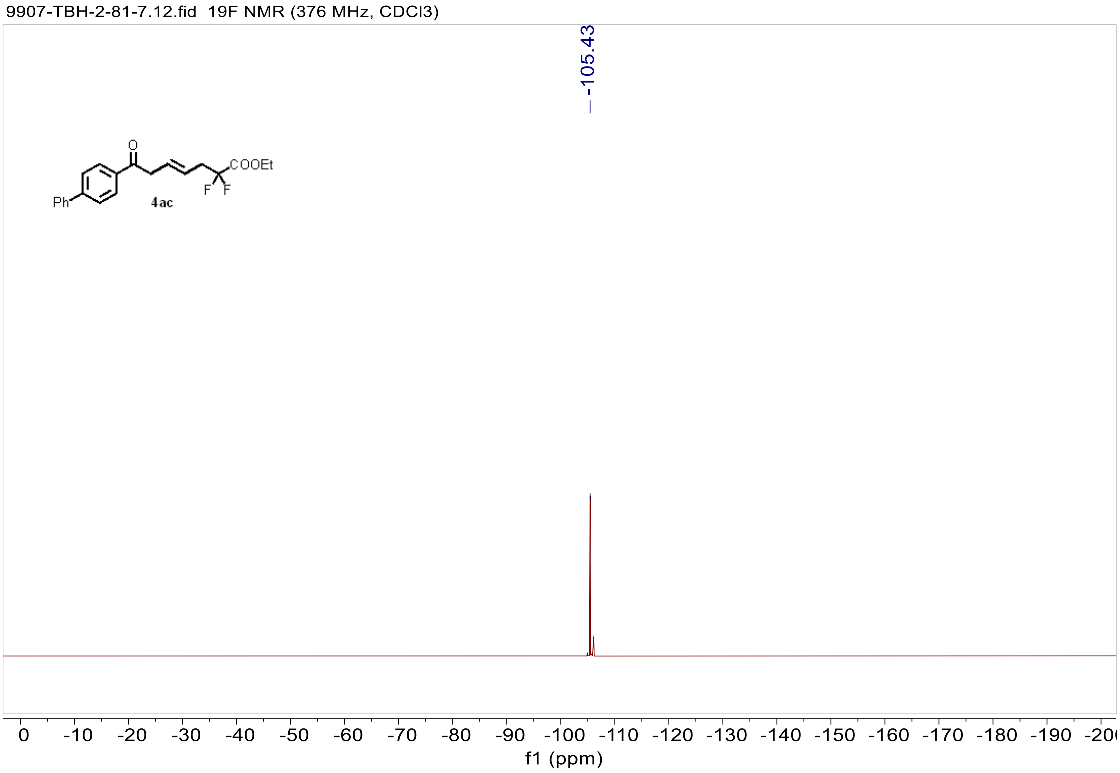

9907-TBH-2-81-7.12.fid 19F NMR (376 MHz, CDCl3)
-105.43
O
COOEt
Ph
F F
4ac
0
-10
-20
-30
-40
-50
-60
-70
-80
-90
-100
-110
-120
-130
-140
-150
-160
-170
-180
-190
f1 (ppm)

NMR spectrums of **4ad**

9645-TBH-2-81-5.10.fid 1H NMR (400 MHz, CDCl3)

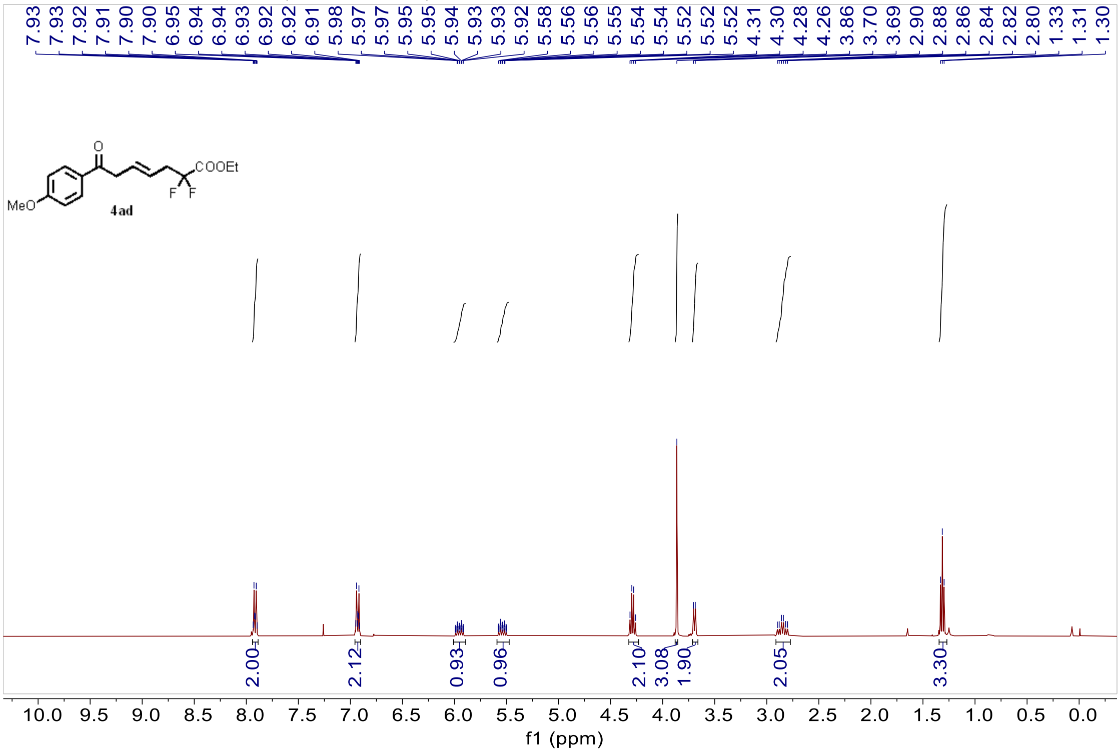

9645-TBH-2-81-5.11.fid 13C NMR (100 MHz, CDCl3)

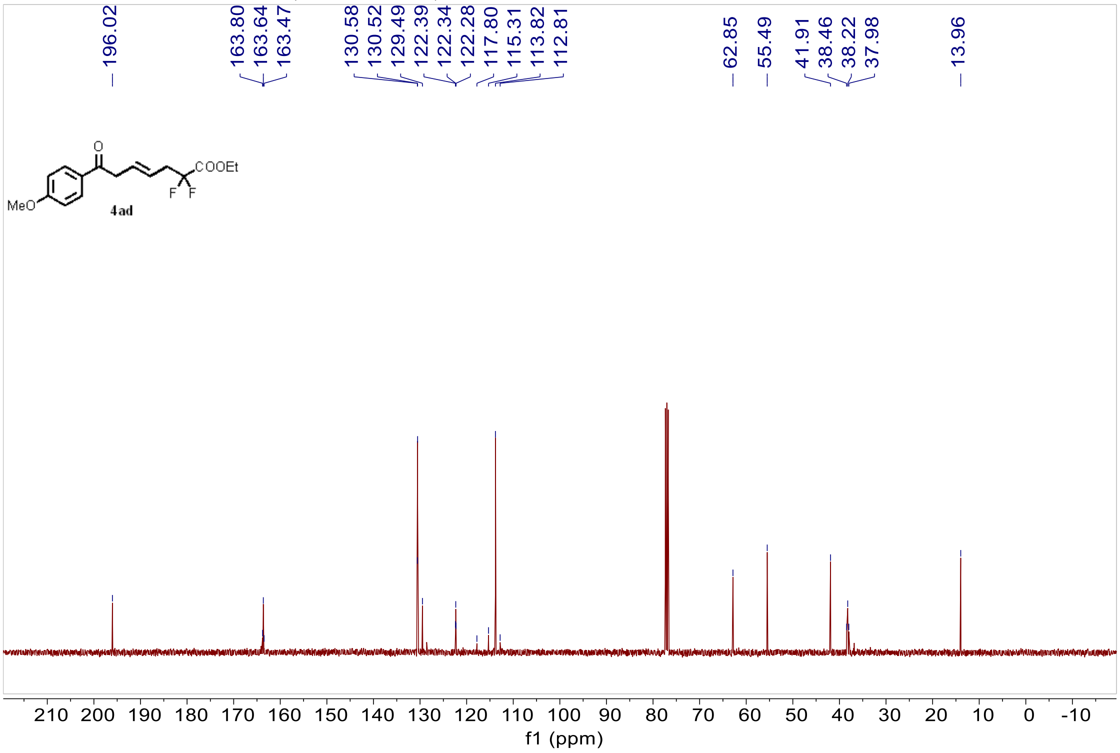

9645-TBH-2-81-5.12.fid 19F NMR (376 MHz, CDCl3)

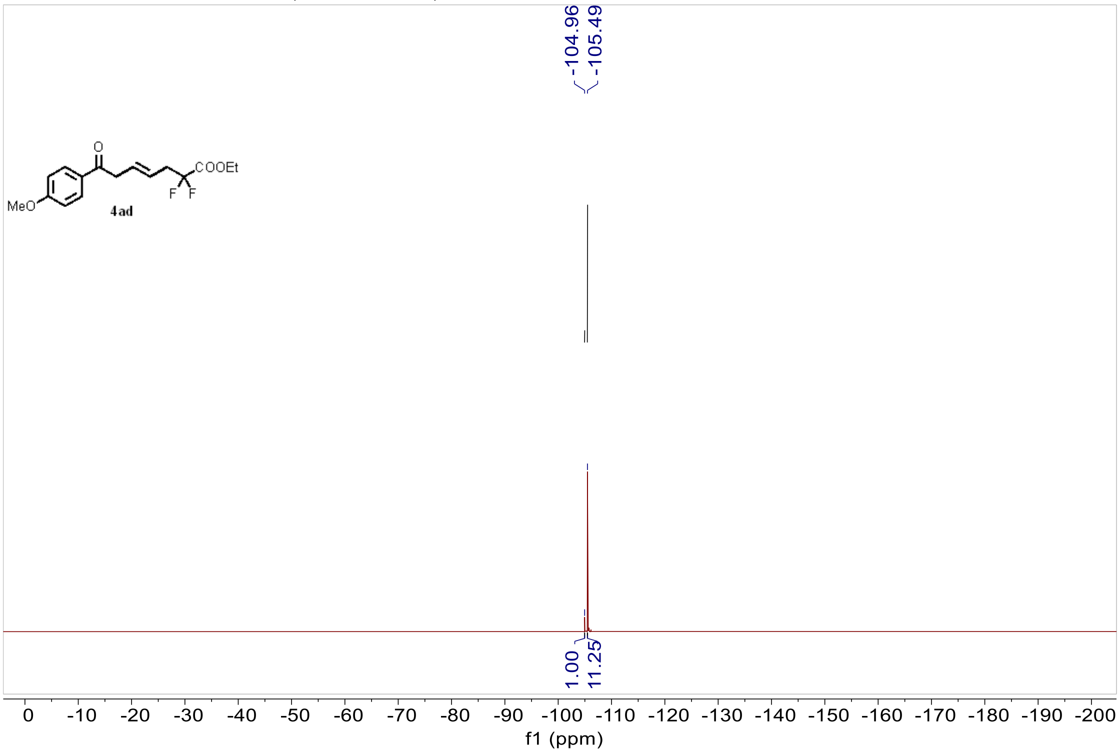

NMR spectrums of **4ae**

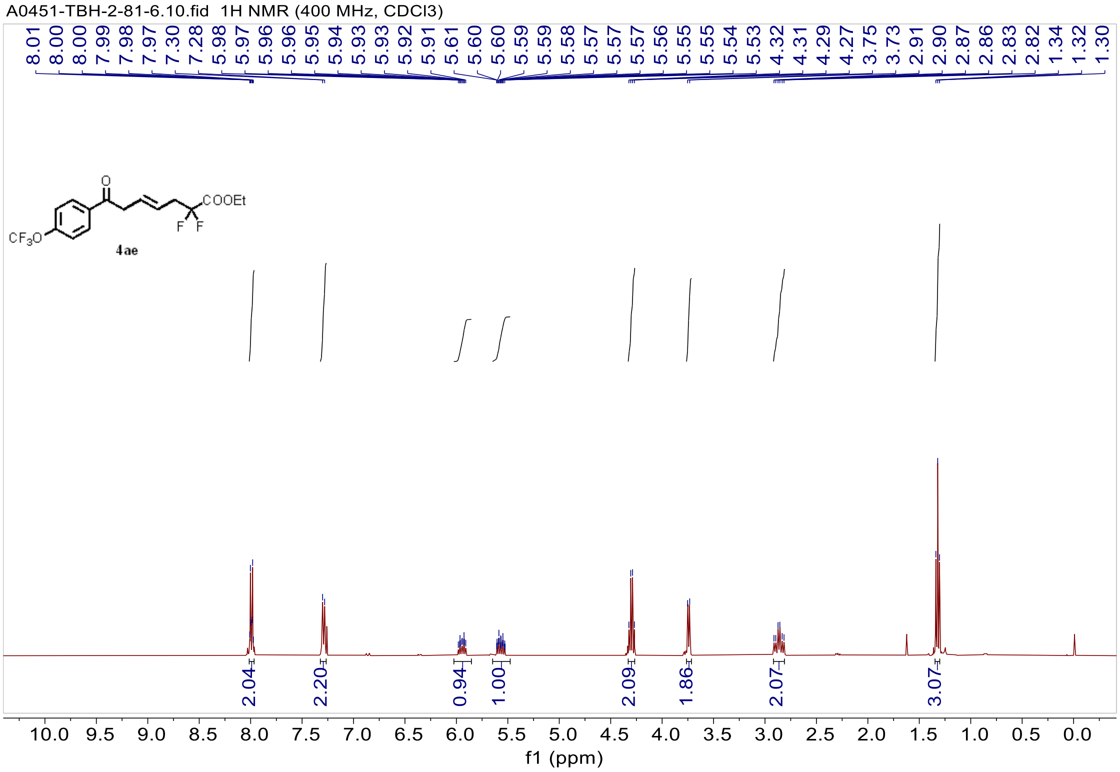

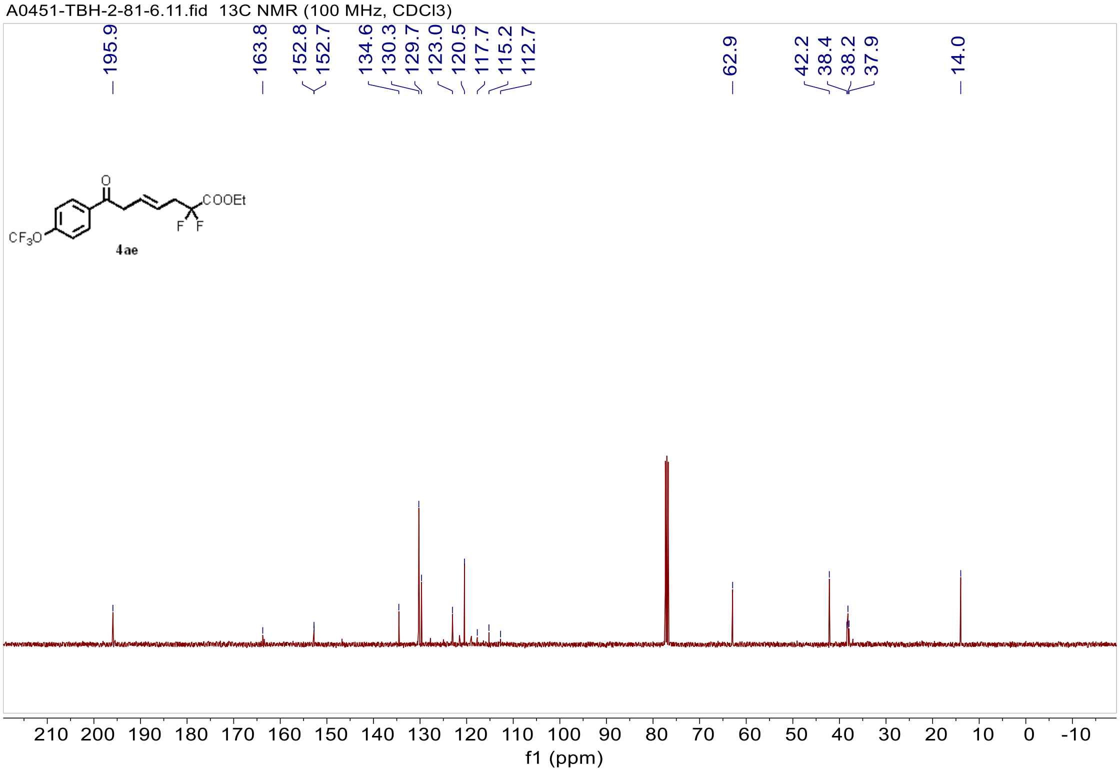

A0451-TBH-2-81-6.12.fid 19F NMR (376 MHz, CDCl3)

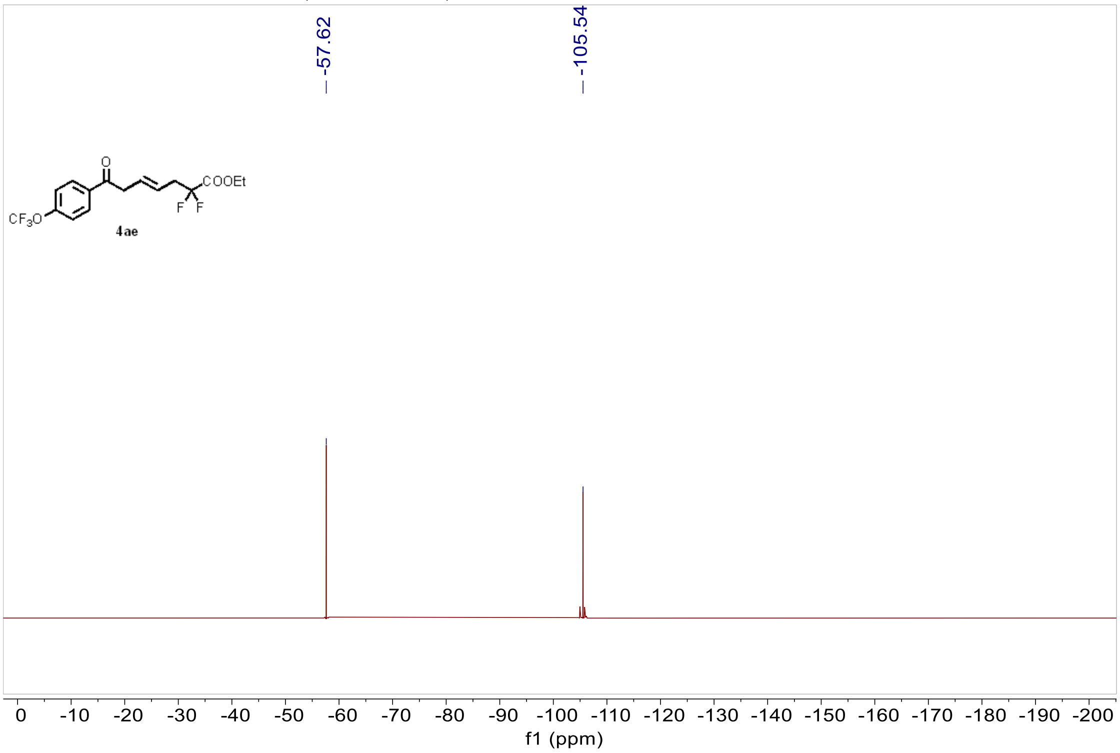

NMR spectrums of **4af**

A1914-TBH-2-81-13.10.fid 1H NMR (400 MHz, CDCl3)

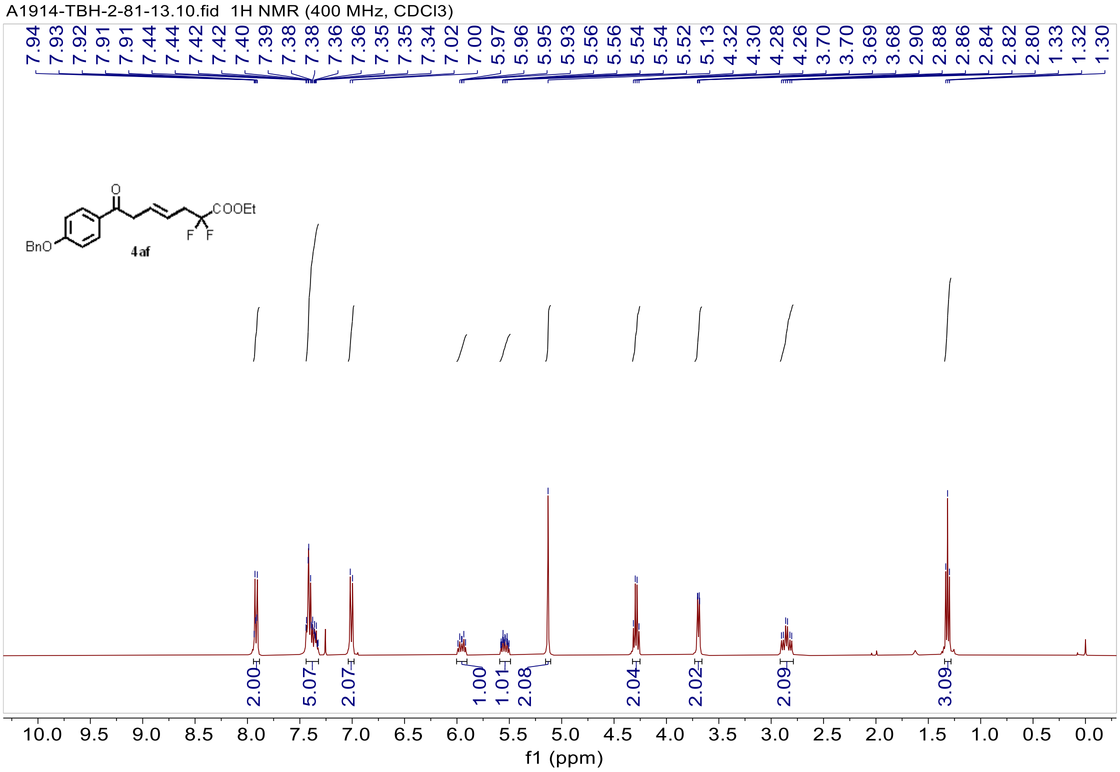

A1914-TBH-2-81-13.11.fid 13C NMR (100 MHz, CDCl3)

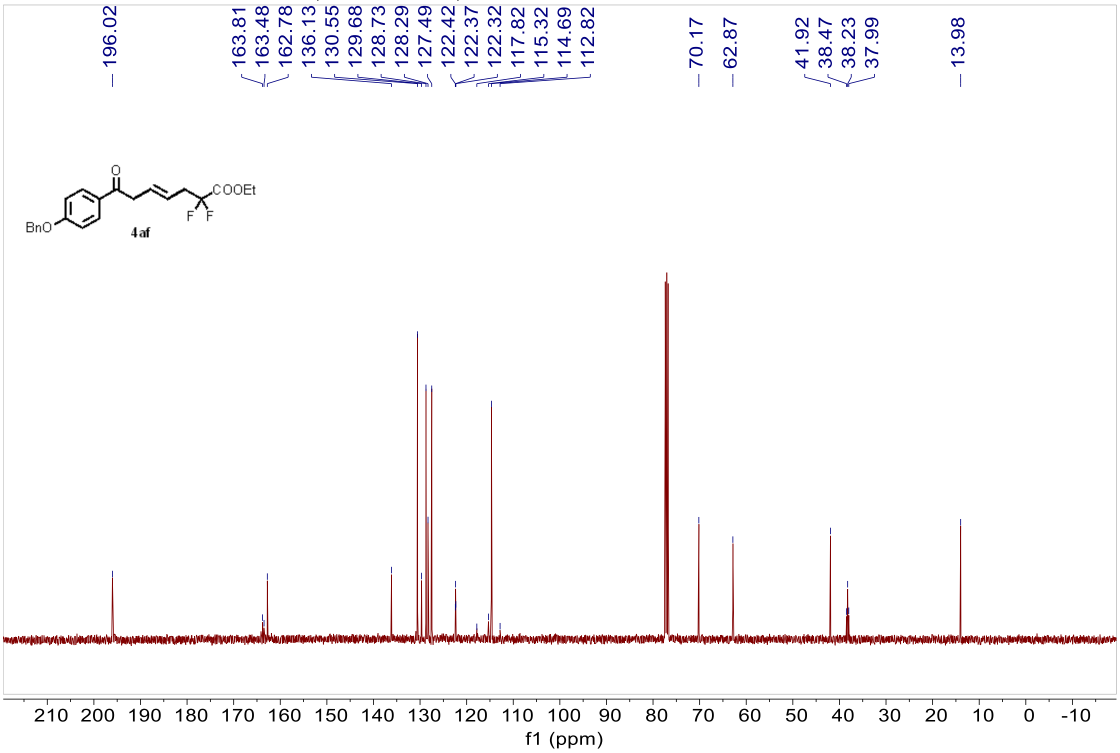

A0159-TBH-2-81-13.12.fid 19F NMR (376 MHz, CDCl3)

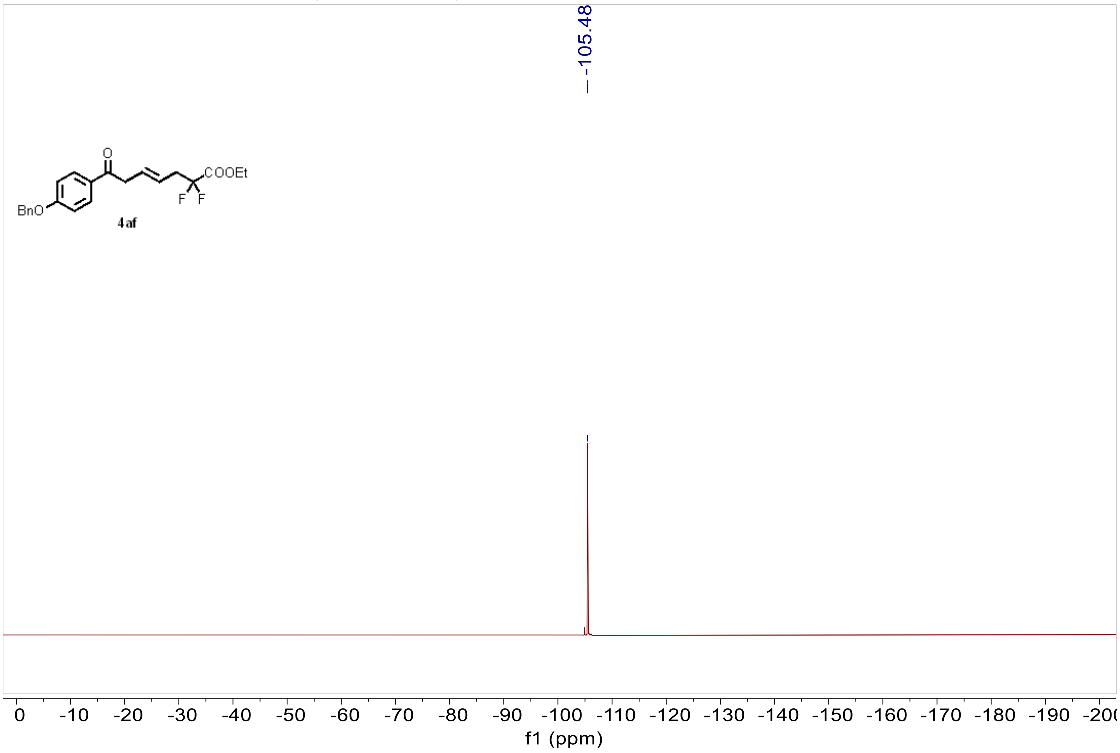

NMR spectrums of **4ag**

9997-TBH-2-81-8.10.fid 1H NMR (400 MHz, CDCl3)

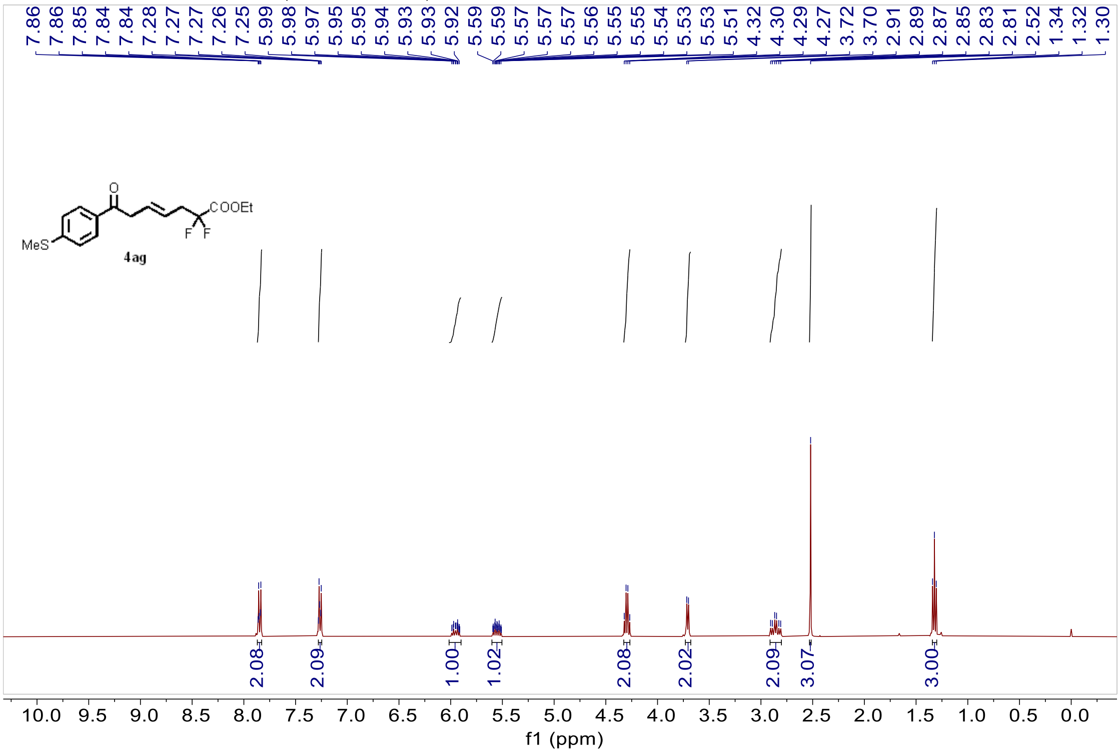

9997-TBH-2-81-8.11.fid 13C NMR (100 MHz, CDCl3)

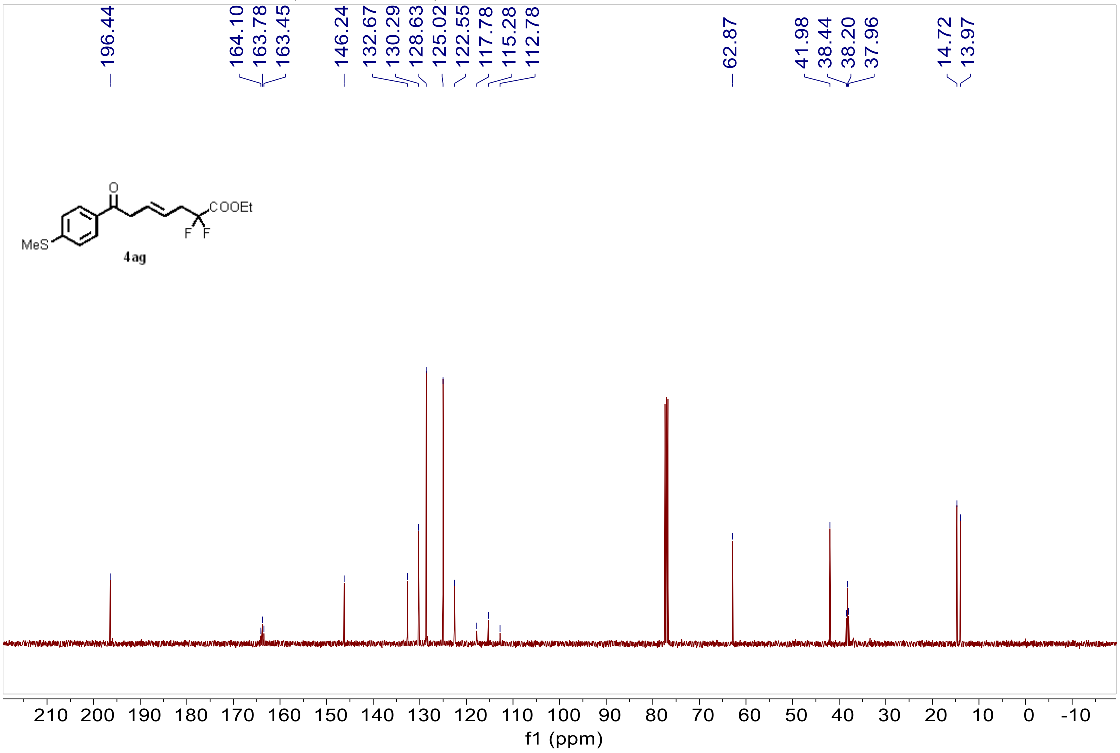

9997-TBH-2-81-8.12.fid 19F NMR (376 MHz, CDCl3)

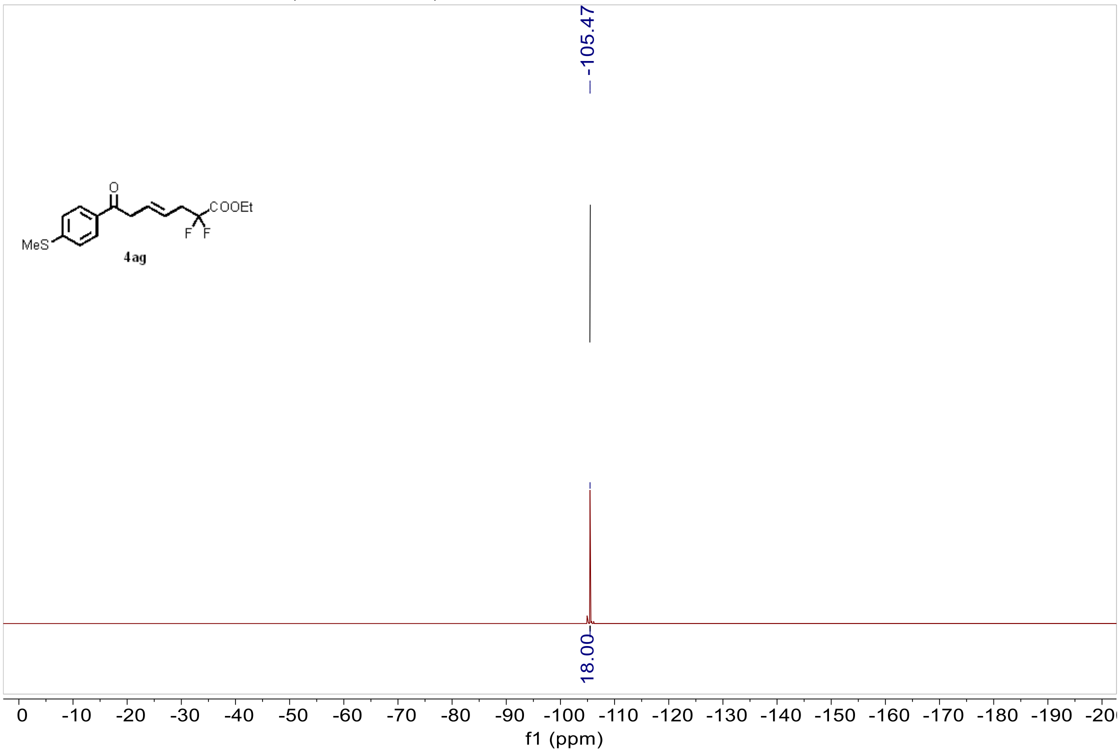

NMR spectrums of **4ah**

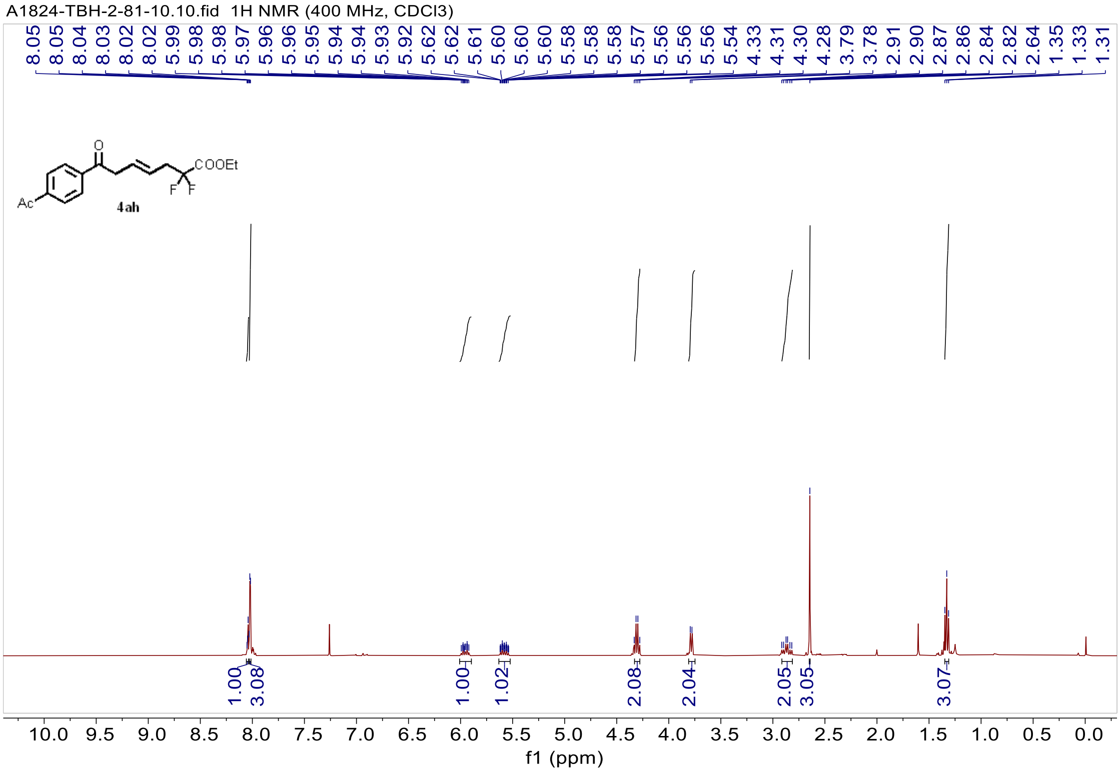

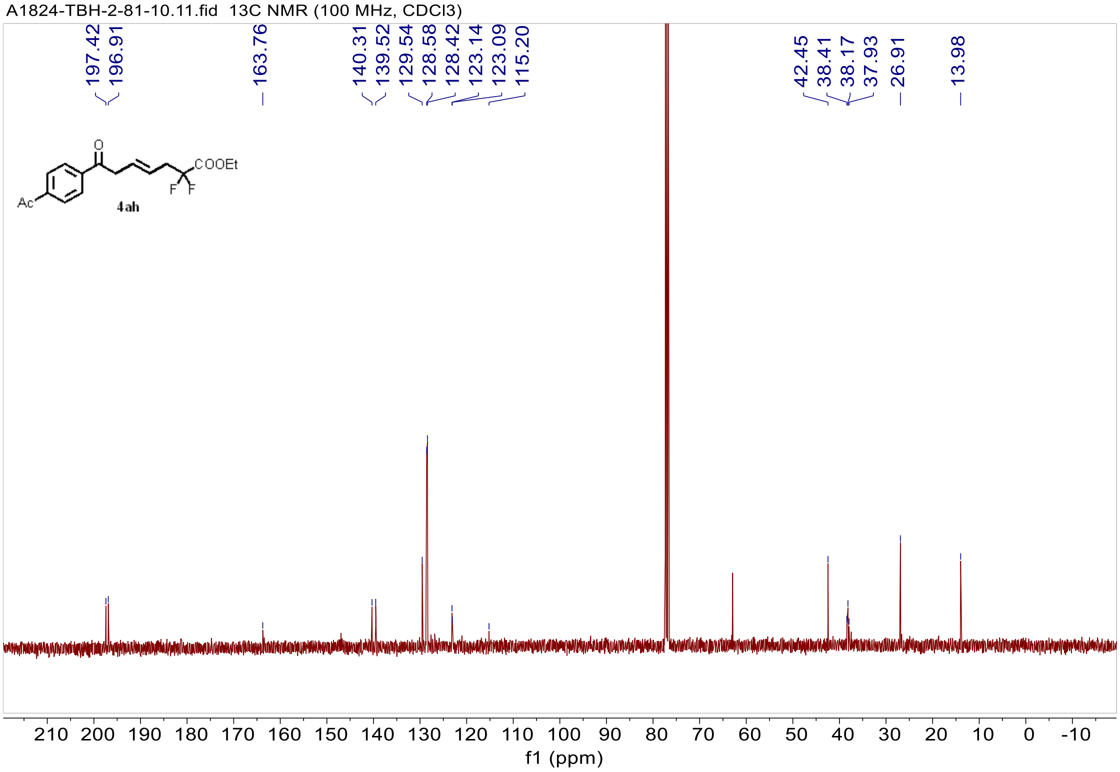

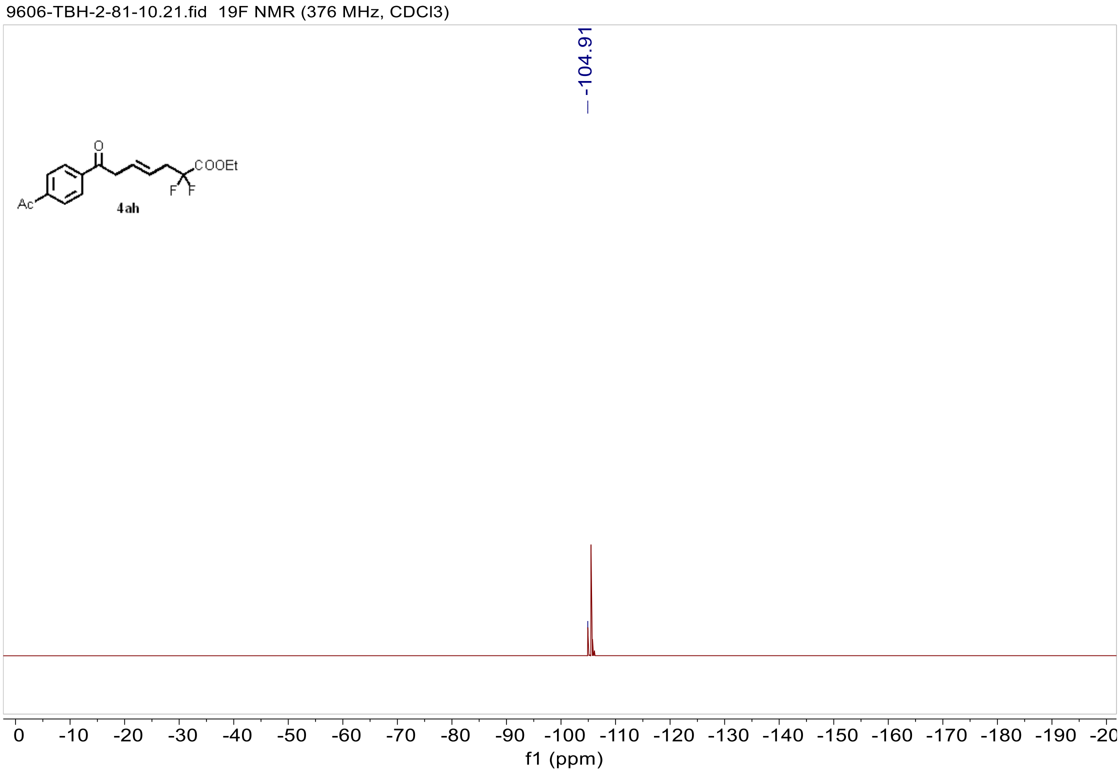

9606-TBH-2-81-10.21.fid 19F NMR (376 MHz, CDCl3)
-104.91
O
COOEt
F F
Ac
4ah
0 -10 -20 -30 -40 -50 -60 -70 -80 -90 -100 -110 -120 -130 -140 -150 -160 -170 -180 -190 -20
f1 (ppm)

NMR spectrums of **4ai**

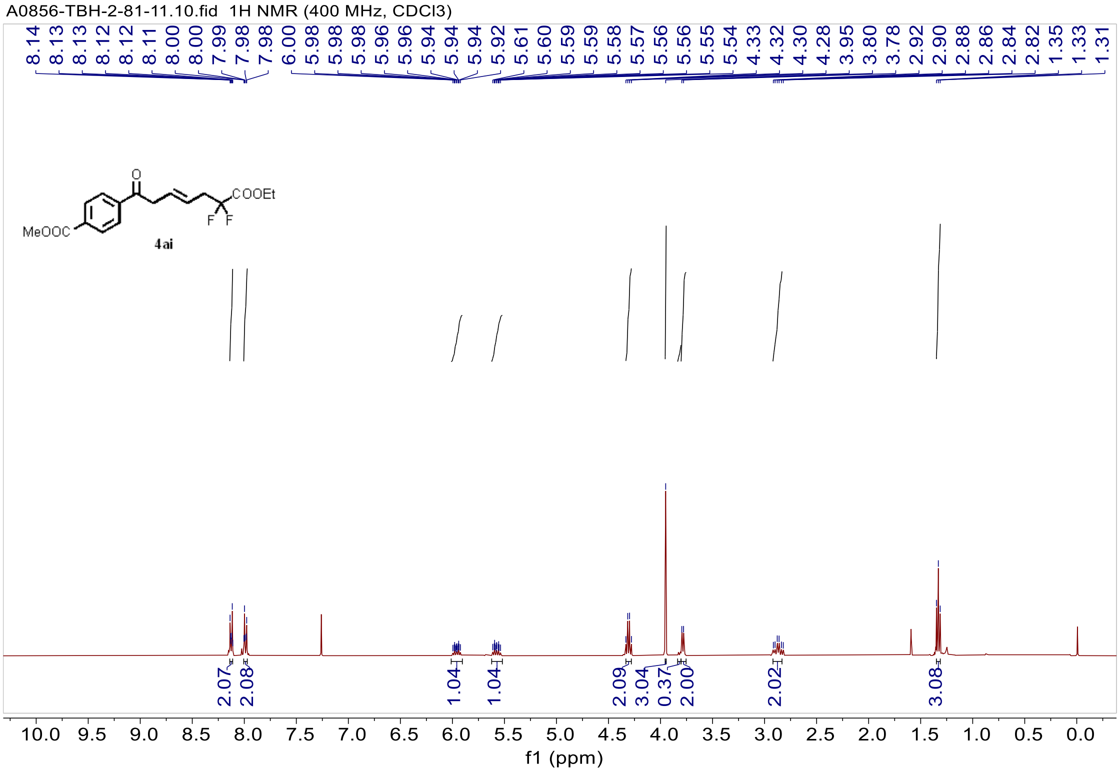

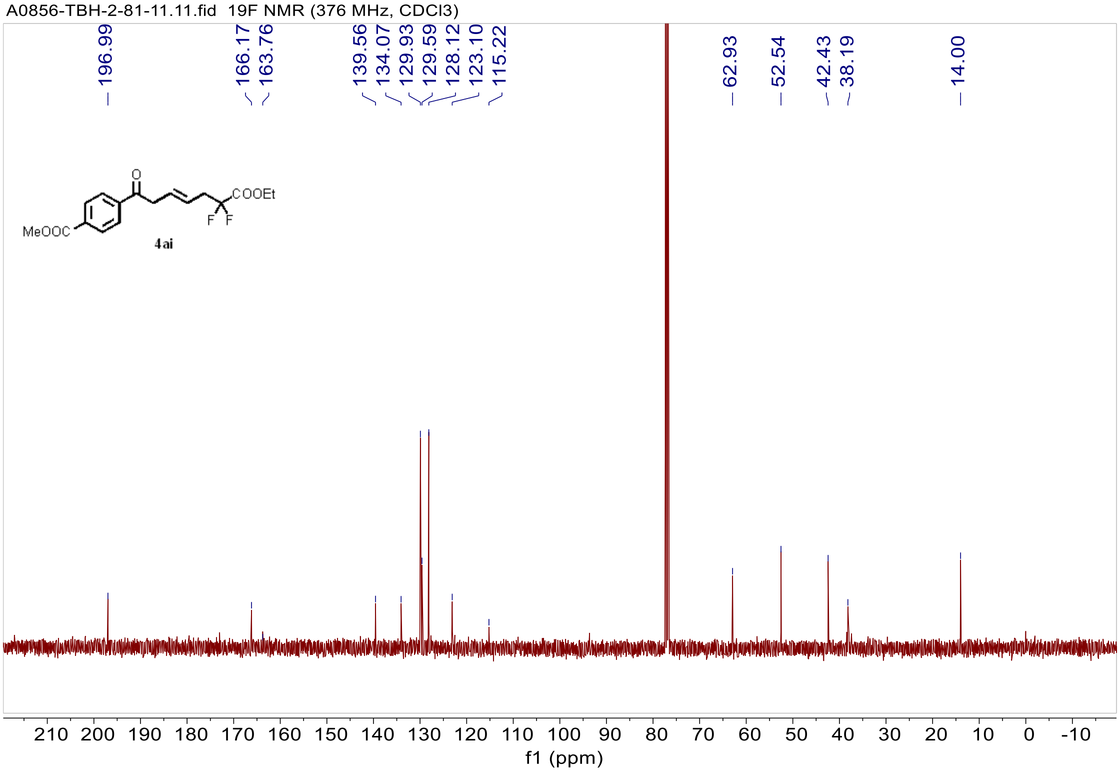

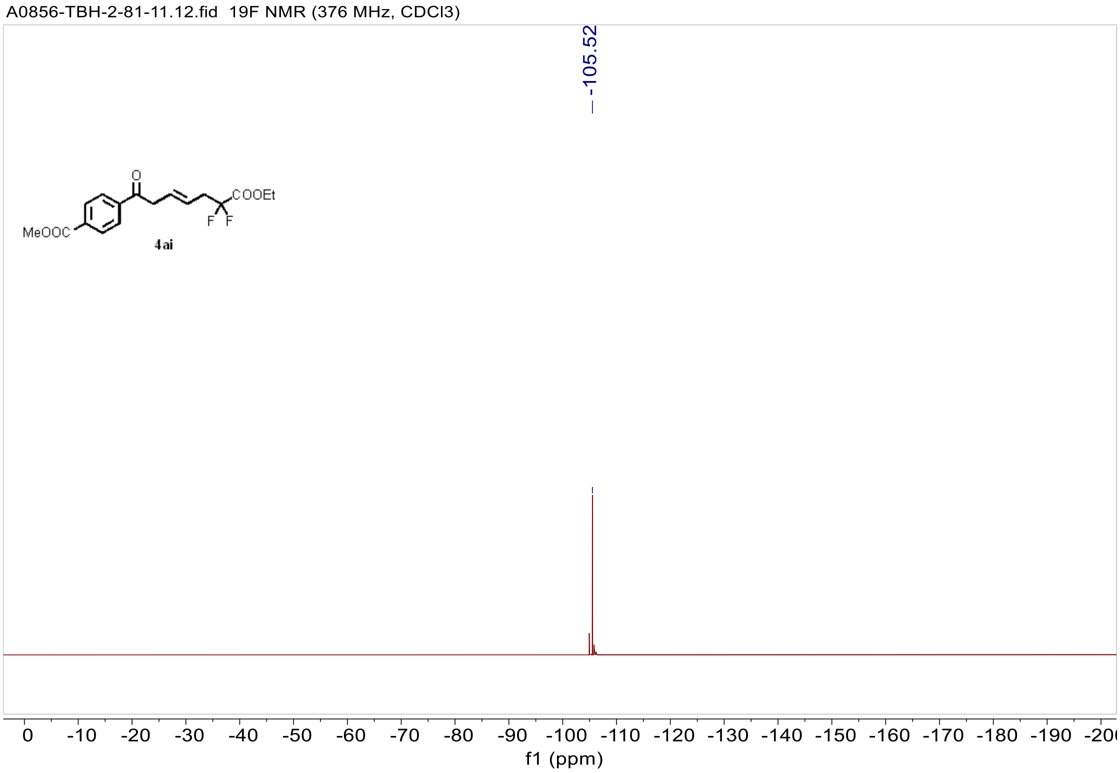
A0856-TBH-2-81-11.12.fid 19F NMR (376 MHz, CDCl3)
-105.52
O
COOEt
MeOOC
F F
4ai
0 -10 -20 -30 -40 -50 -60 -70 -80 -90 -100 -110 -120 -130 -140 -150 -160 -170 -180 -190 -200
f1 (ppm)

NMR spectrums of **4aj**

5441-TBH-2-81-18.10.fid 1H NMR (400 MHz, CDCl3)

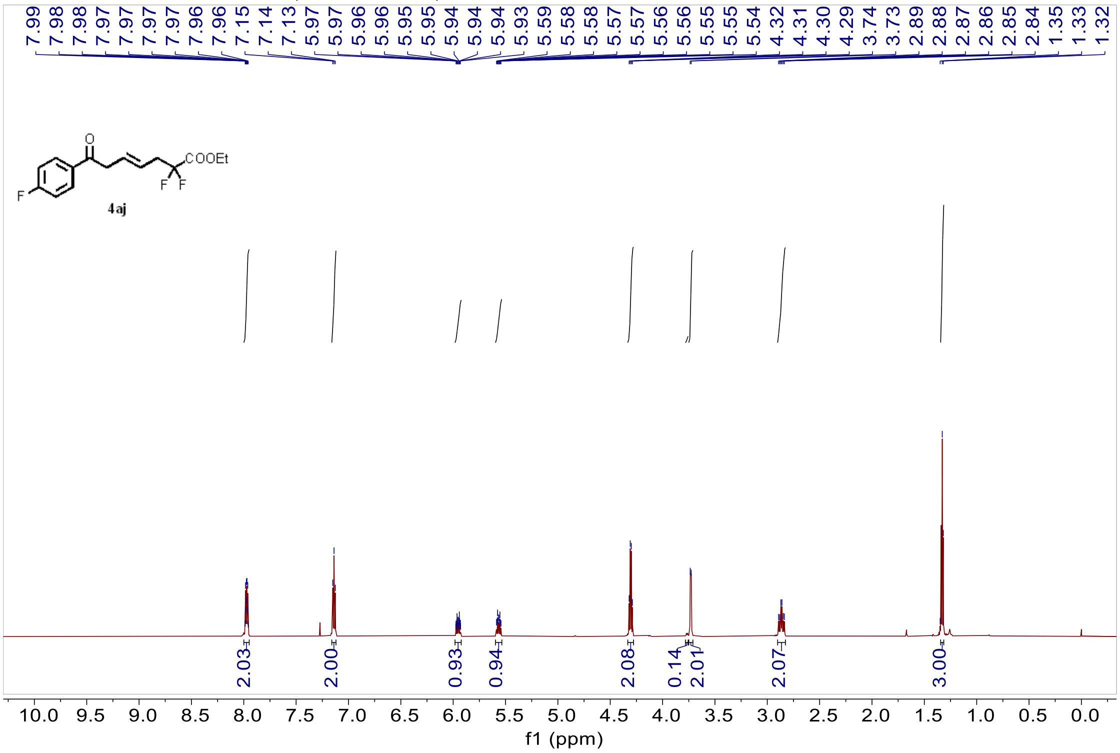

5441-TBH-2-81-18.11.fid 13C NMR (100 MHz, CDCl3)

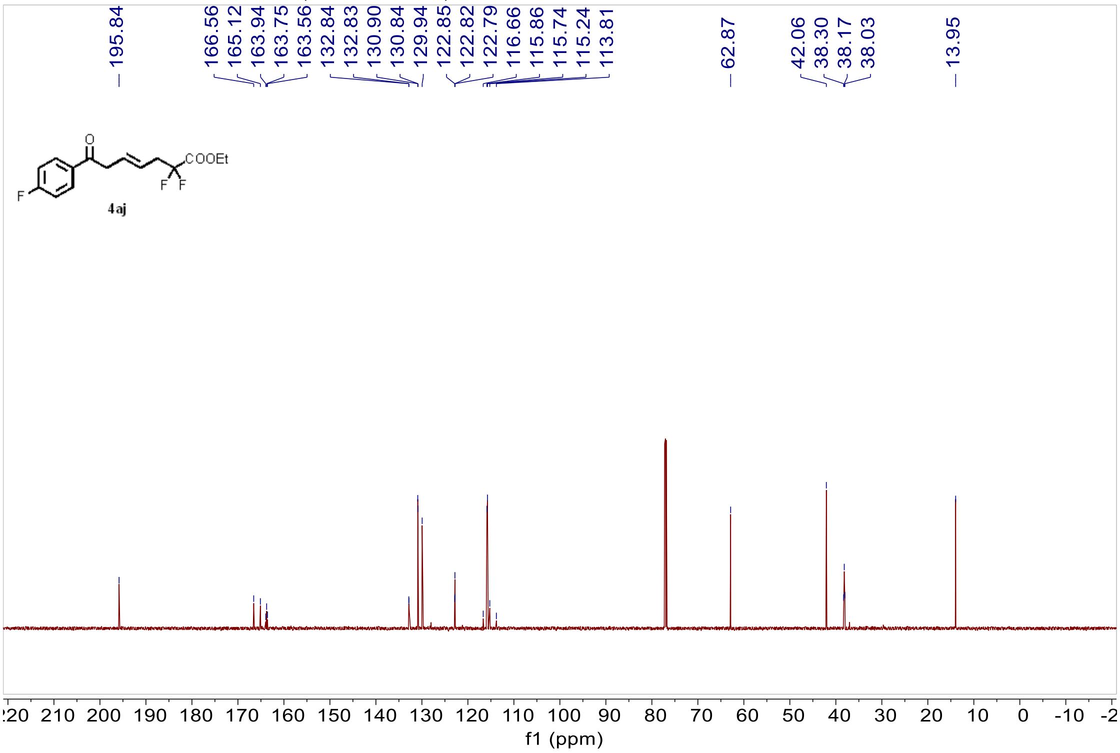

A0956-TBH-2-81-18.20.fid 19F NMR (376 MHz, CDCl3)

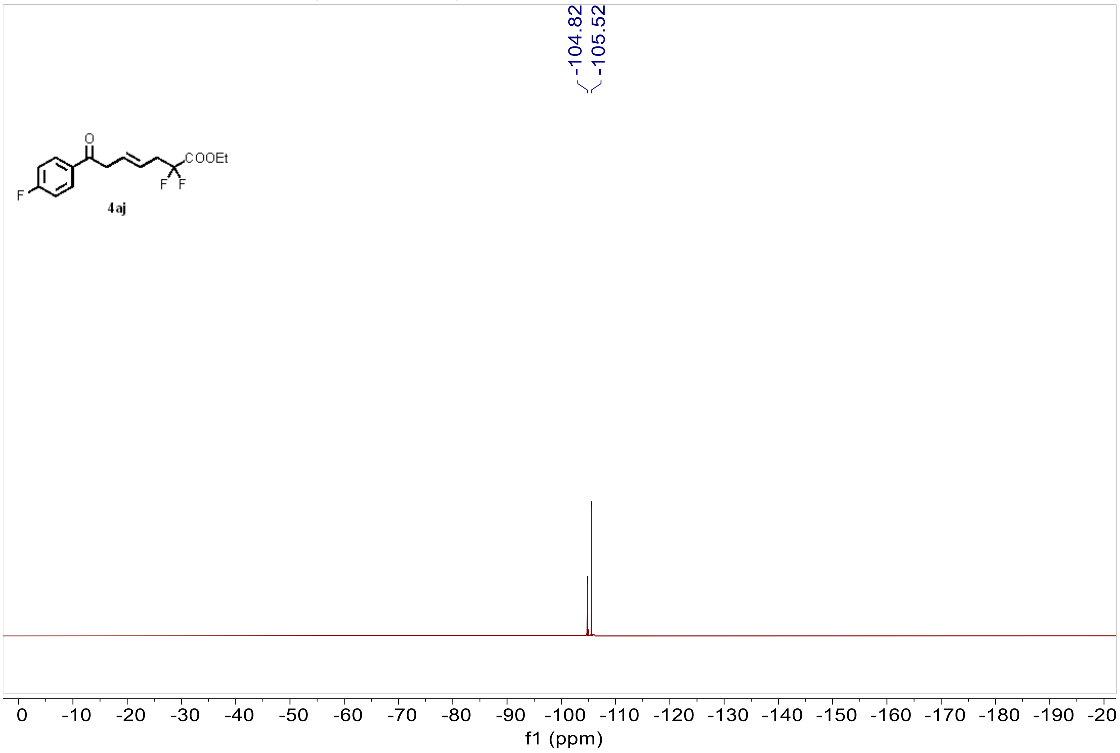

NMR spectrums of **4ak**

5441-TBH-2-81-19.10.fid 1H NMR (400 MHz, CDCl3)

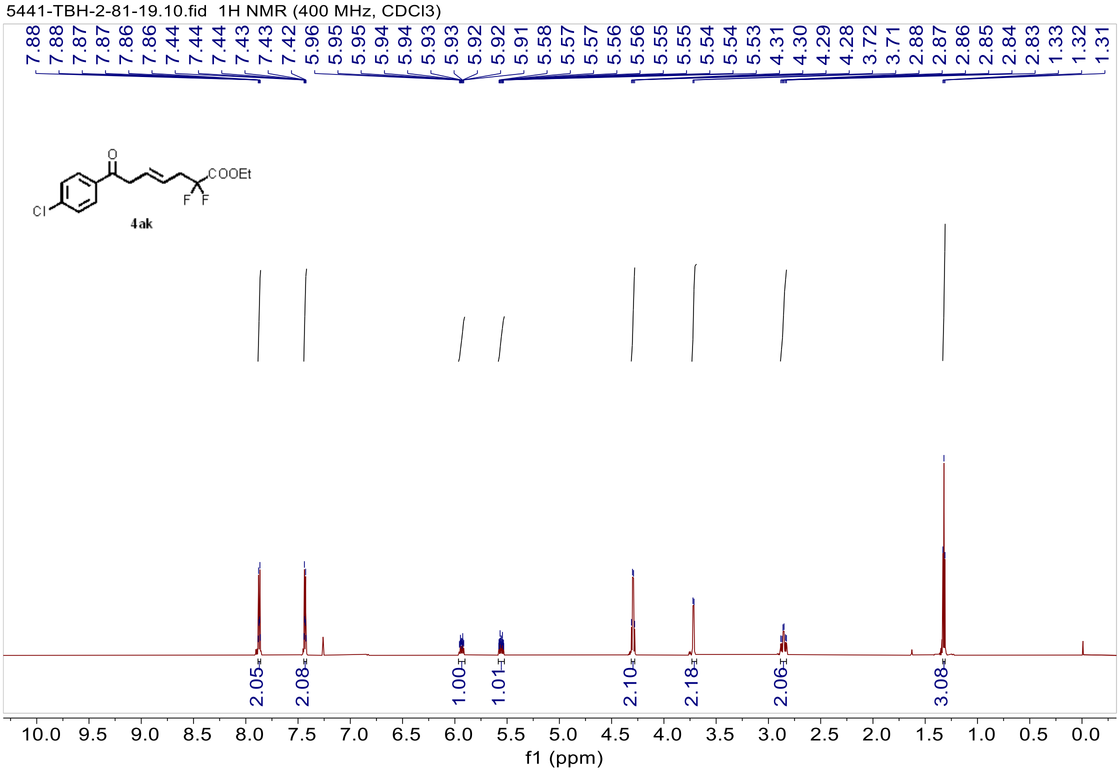

5441-TBH-2-81-19.11.fid 13C NMR (100 MHz, CDCl3)

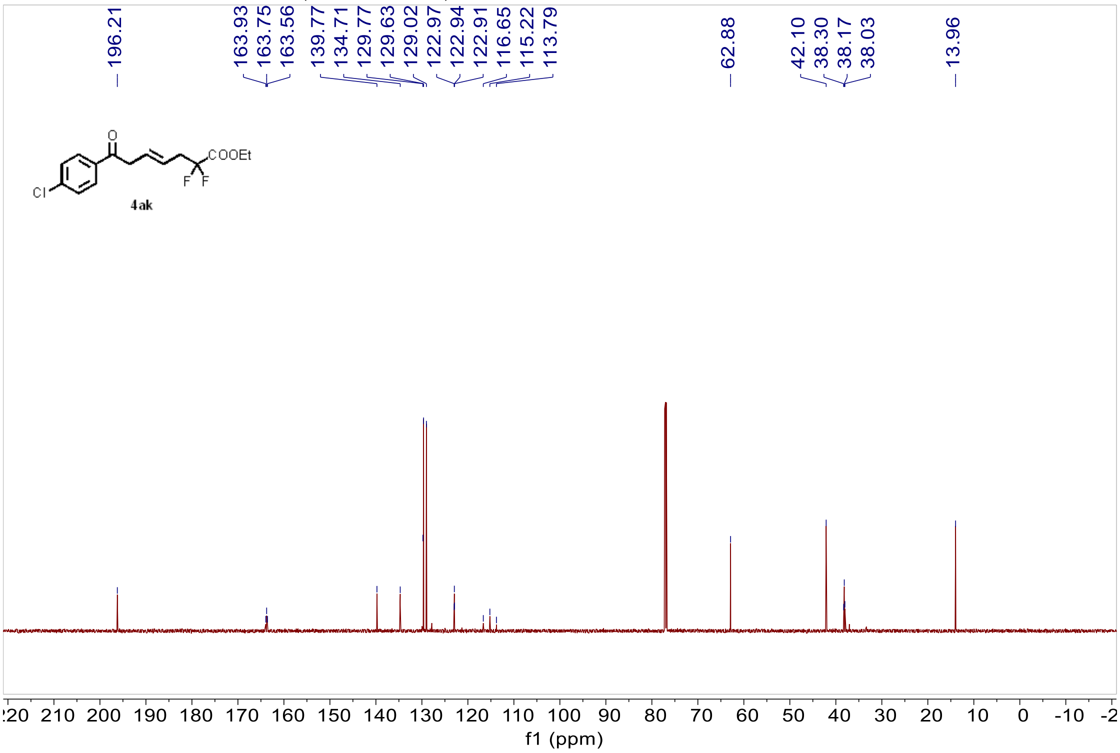

A0956-TBH-2-81-19.20.fid 19F NMR (376 MHz, CDCl3)

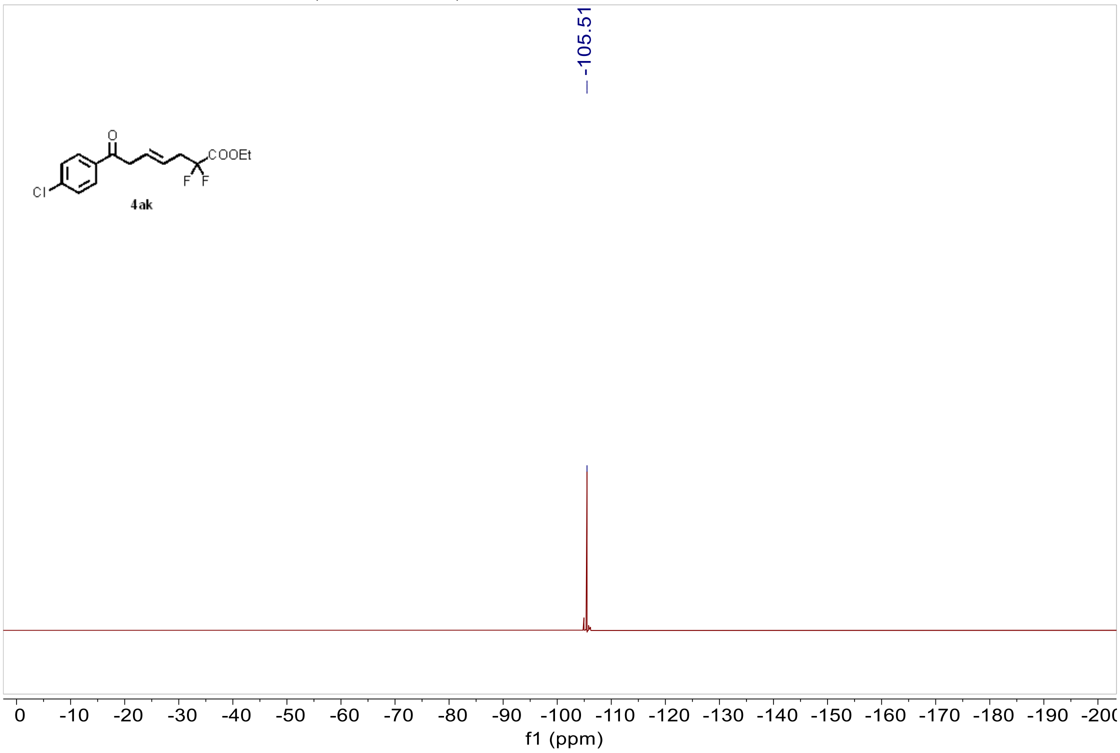

NMR spectrums of **4al**

A0614-TBH-2-81-20.10.fid 1H NMR (400 MHz, CDCl3)

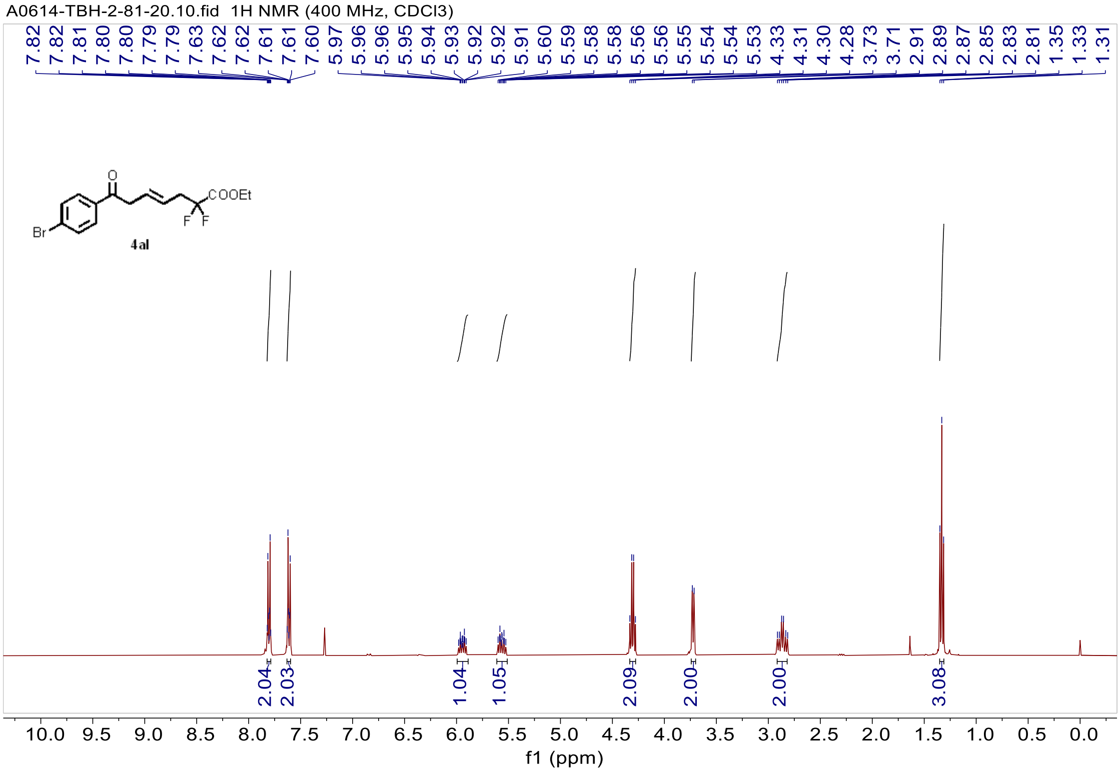

A0614-TBH-2-81-20.11.fid 13C NMR (100 MHz, CDCl3)

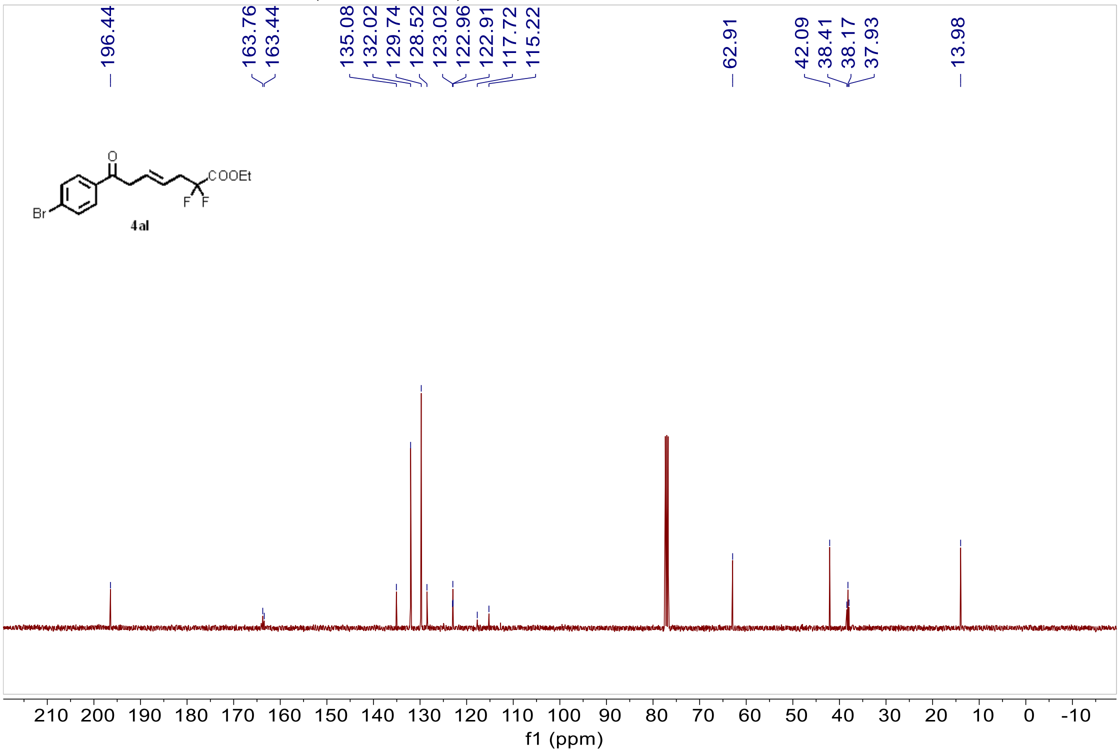

A0614-TBH-2-81-20.12.fid 19F NMR (376 MHz, CDCl3)

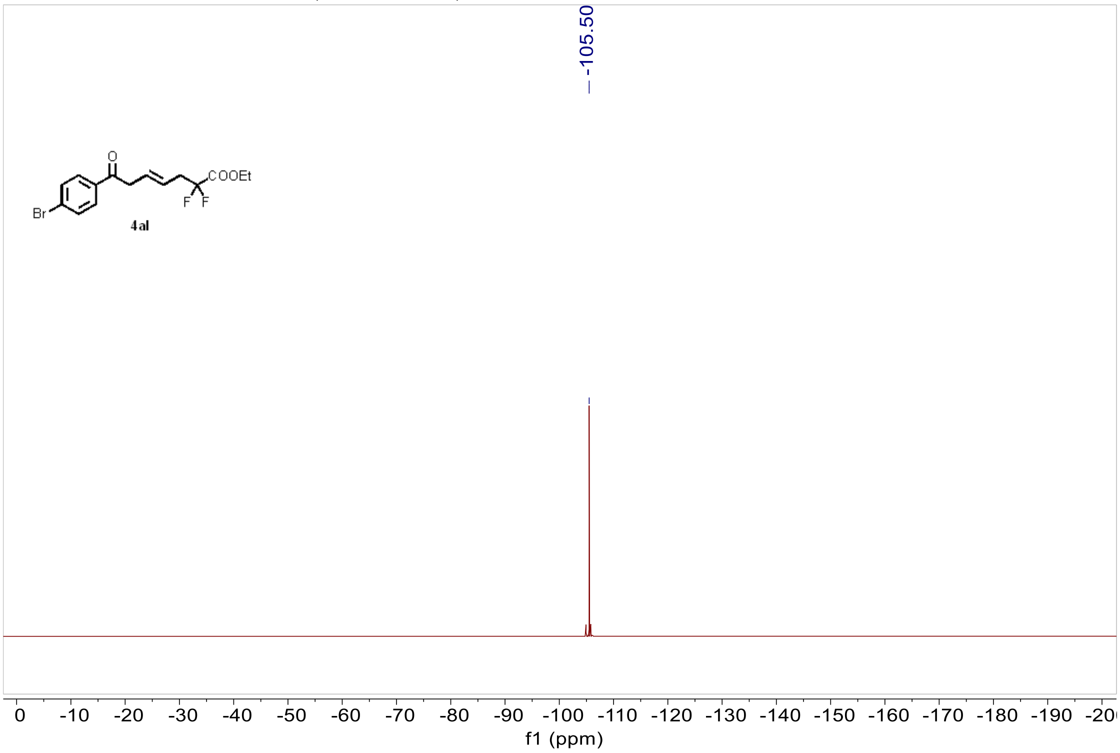

NMR spectrums of **4am**

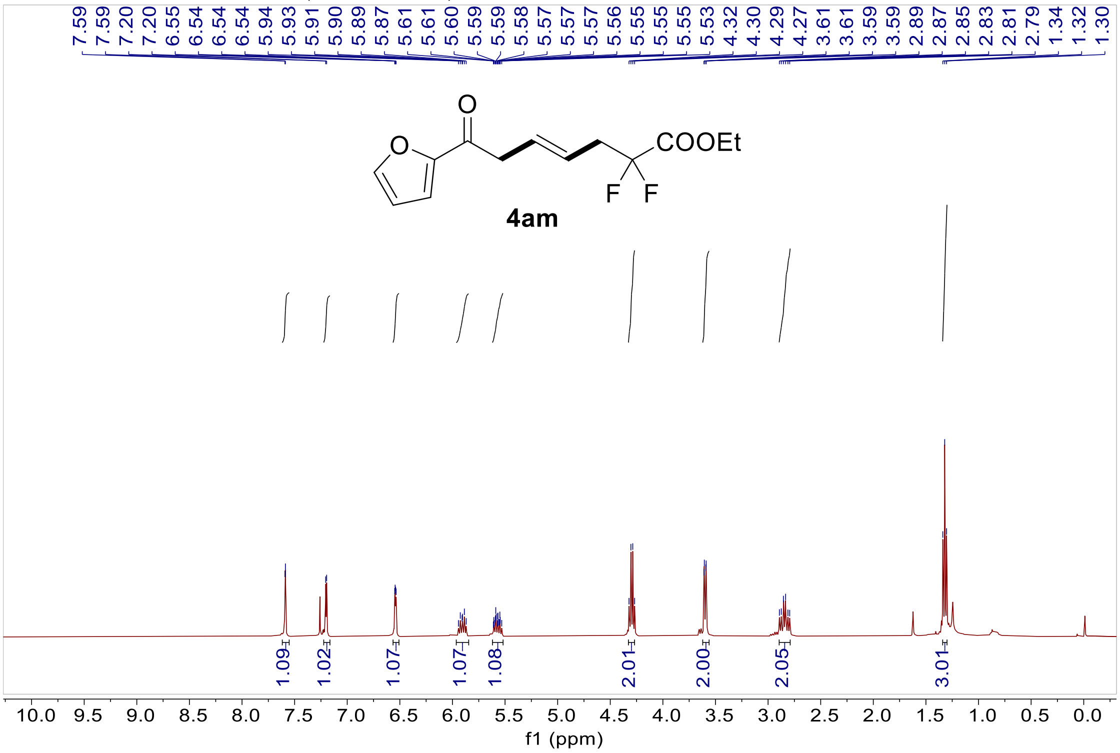

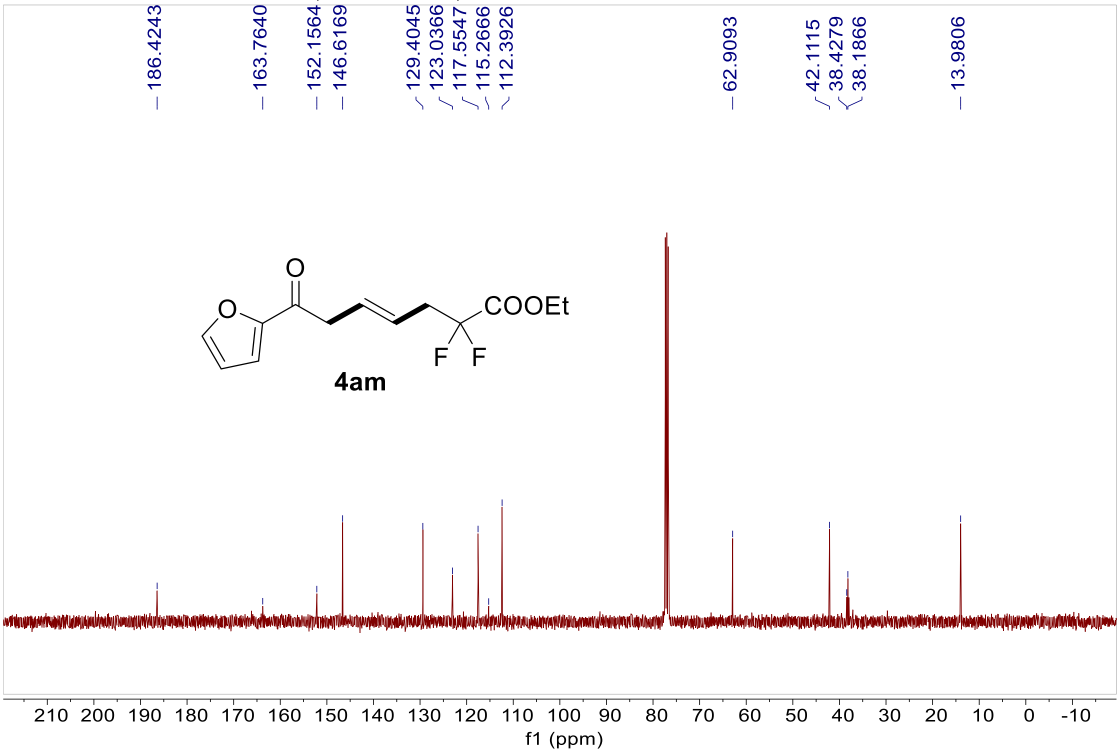

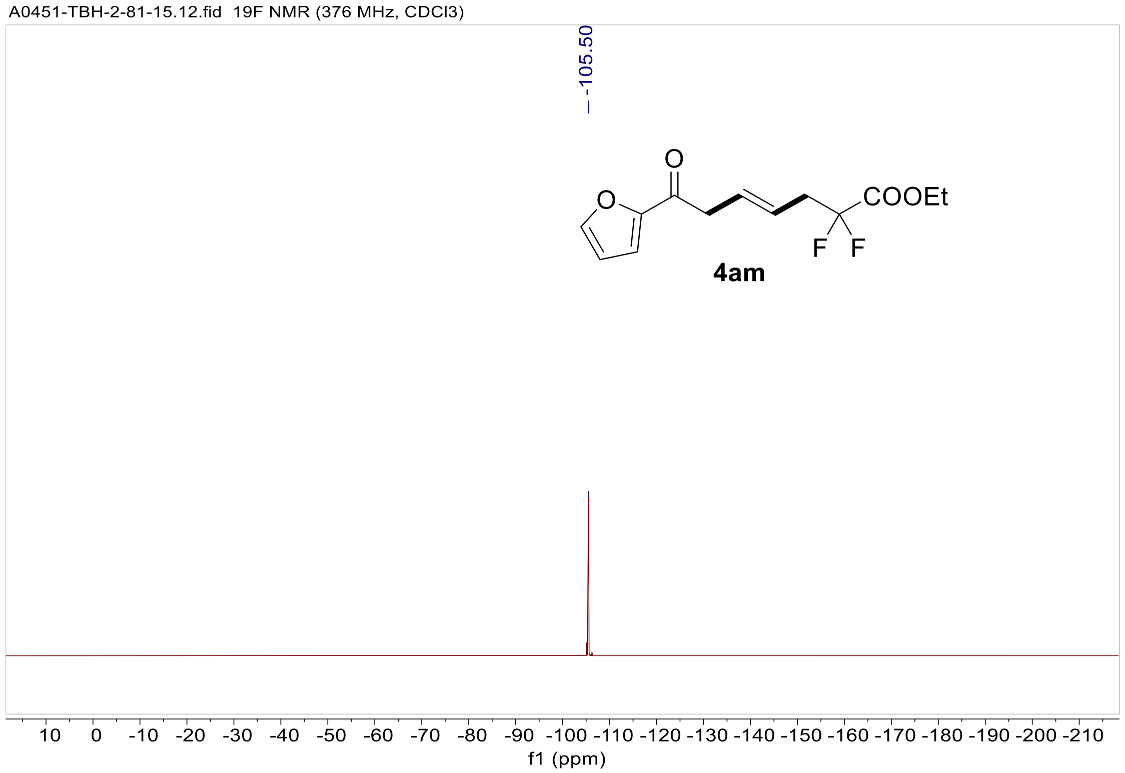

A0451-TBH-2-81-15.12.fid 19F NMR (376 MHz, CDCl3)
-105.50
O
O
COOEt
F F
4am
10 0 -10 -20 -30 -40 -50 -60 -70 -80 -90 -100 -110 -120 -130 -140 -150 -160 -170 -180 -190 -200 -210
f1 (ppm)

NMR spectrums of **4ba**

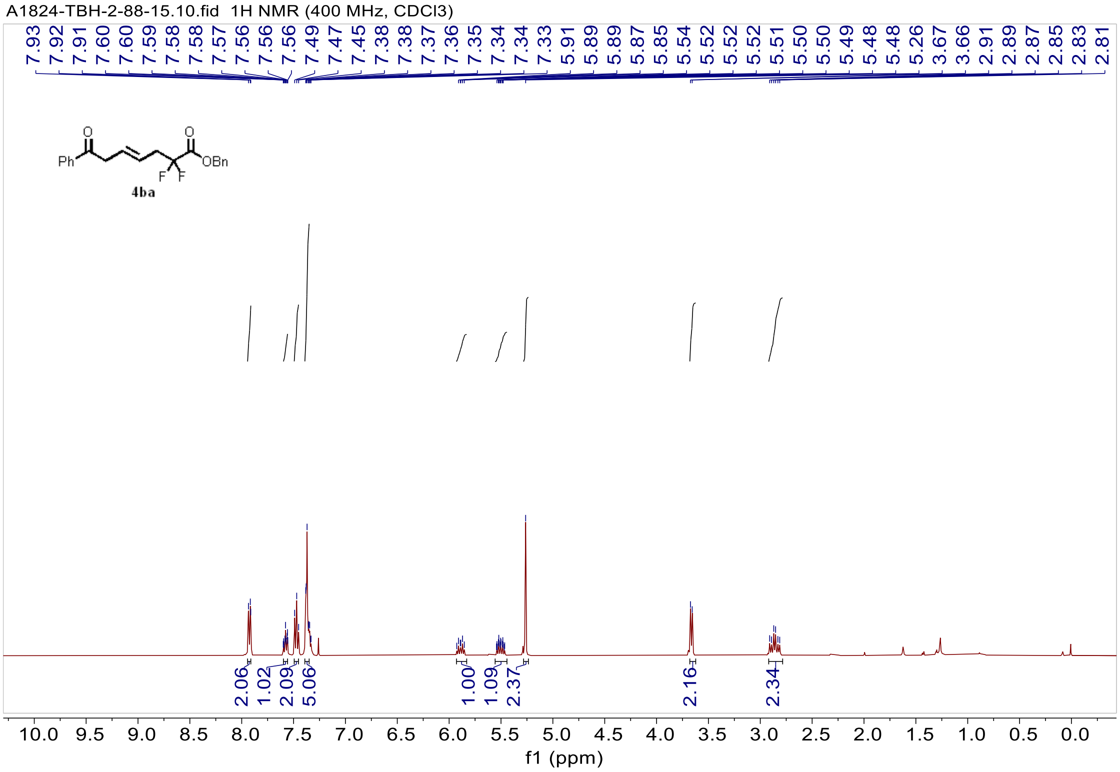

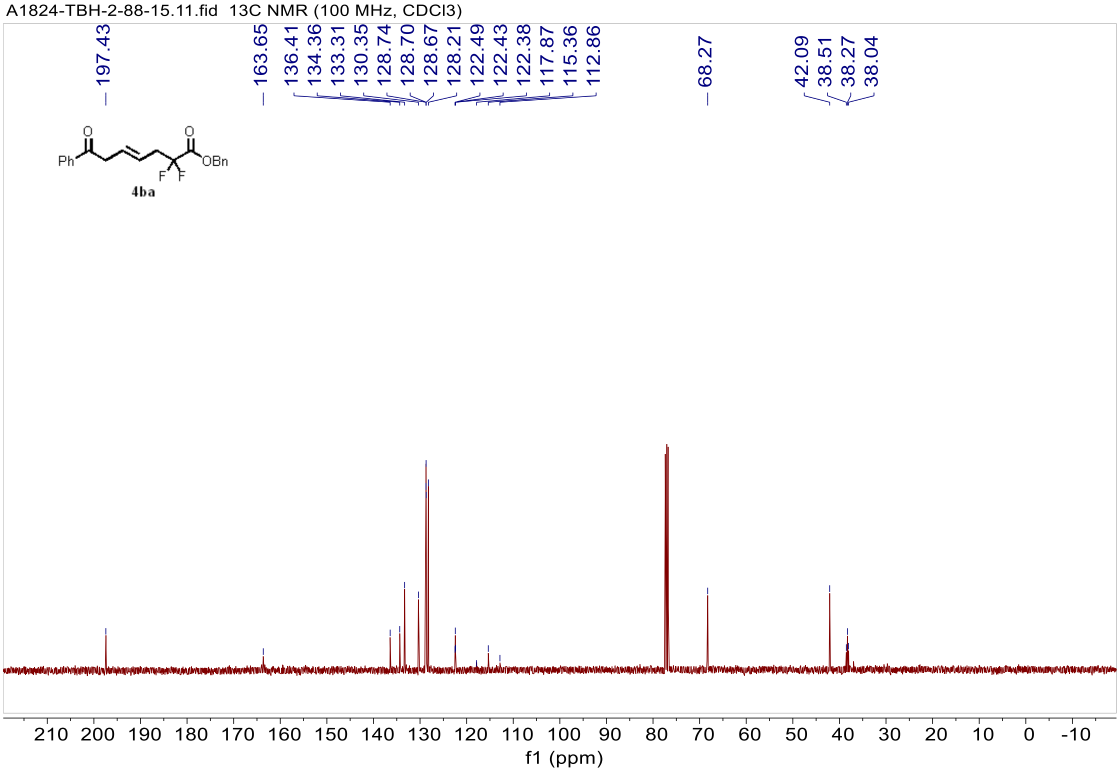

A1824-TBH-2-88-15.12.fid 19F NMR (376 MHz, CDCl3)

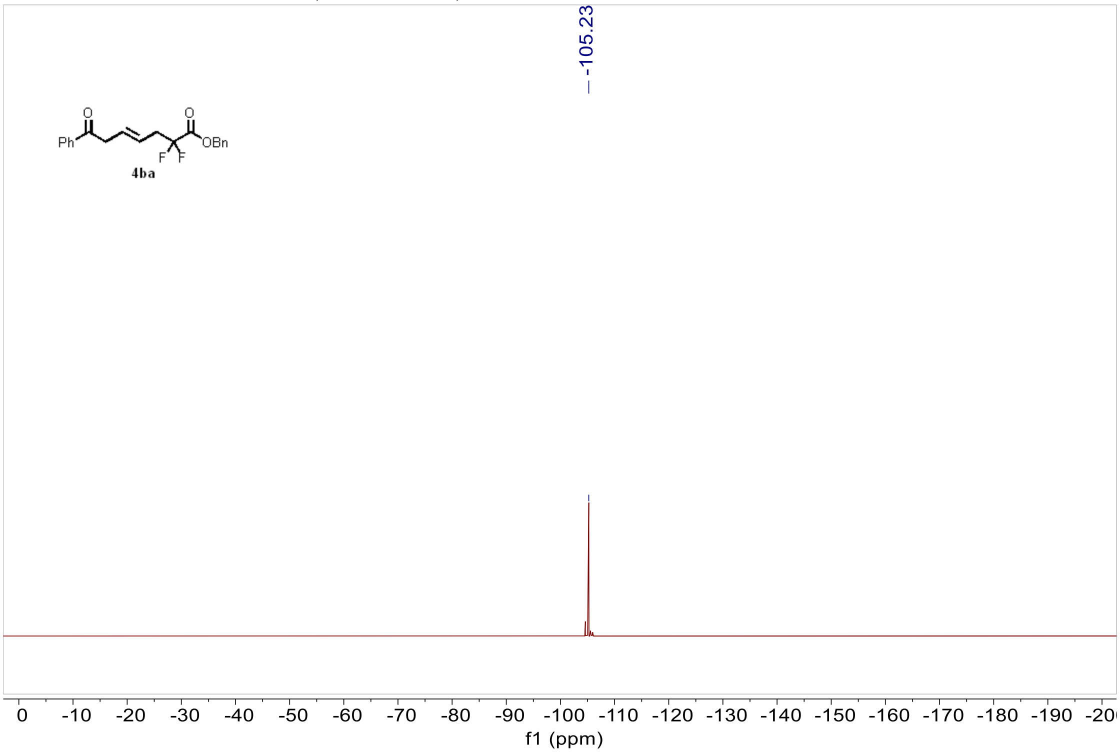

NMR spectrums of **4bb**

A0745-TBH-2-88-2.10.fid 1H NMR (400 MHz, CDCl3)

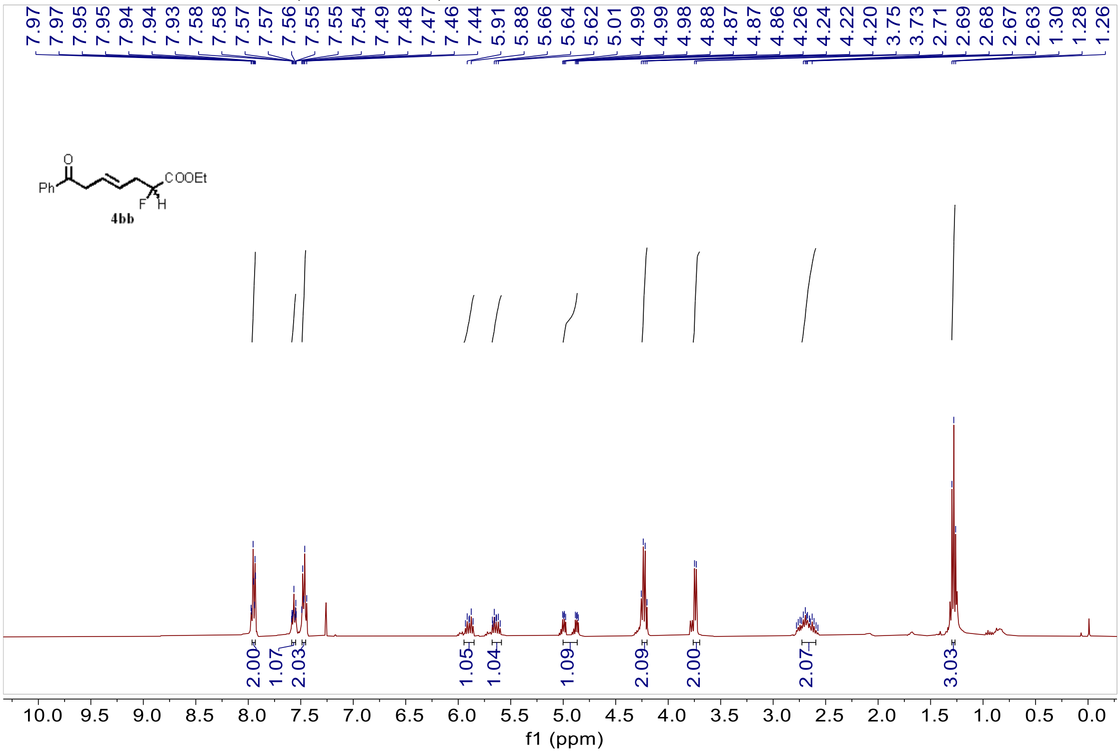

A0745-TBH-2-88-2.11.fid 13C NMR (100 MHz, CDCl3)

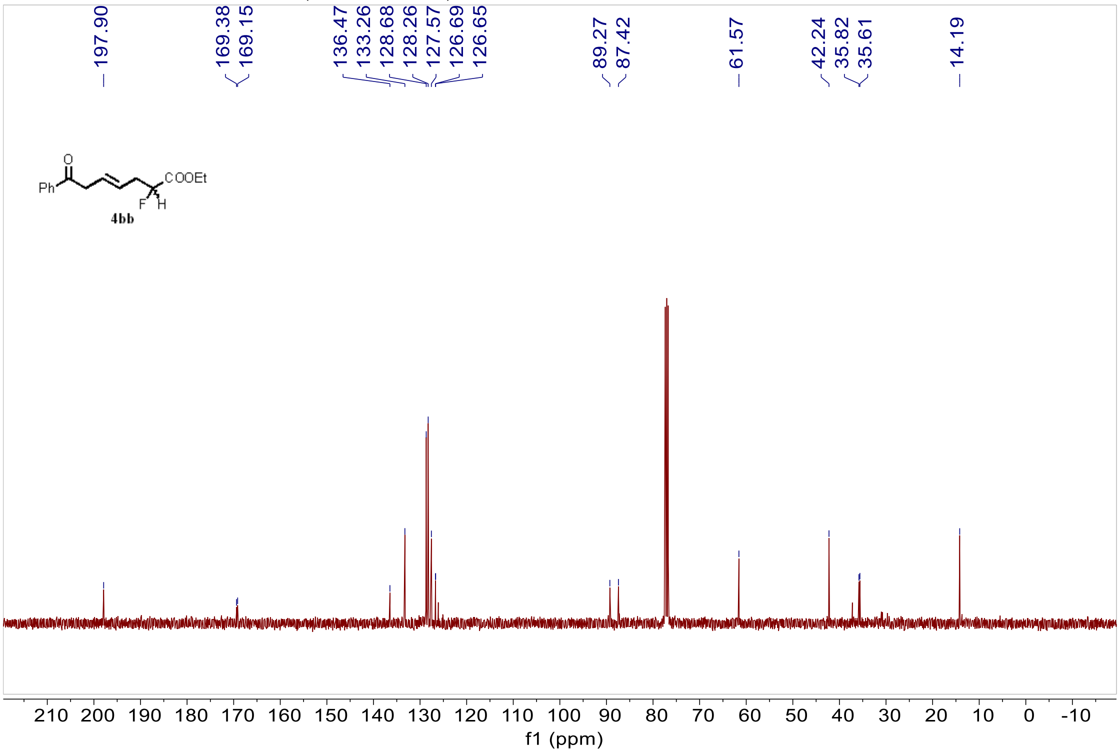

A0745-TBH-2-88-2.12.fid 19F NMR (376 MHz, CDCl3)

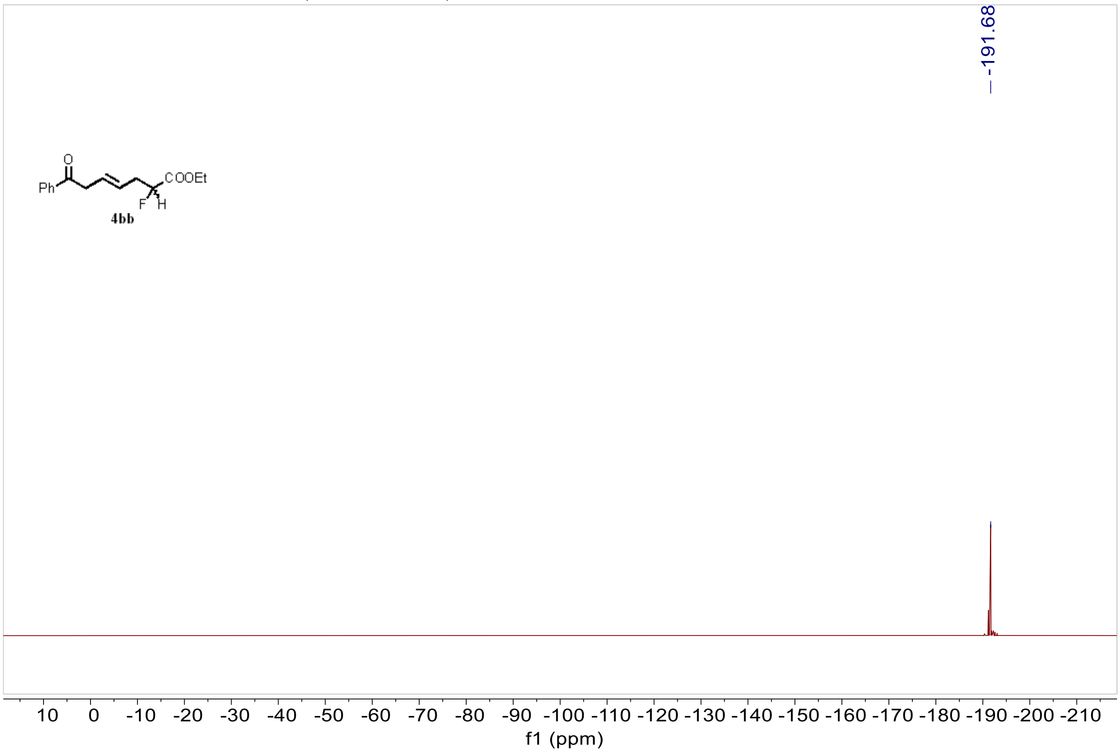

NMR spectrums of **4bc**

A0762-TBH-2-88-3.10.fid 1H NMR (400 MHz, CDCl3)

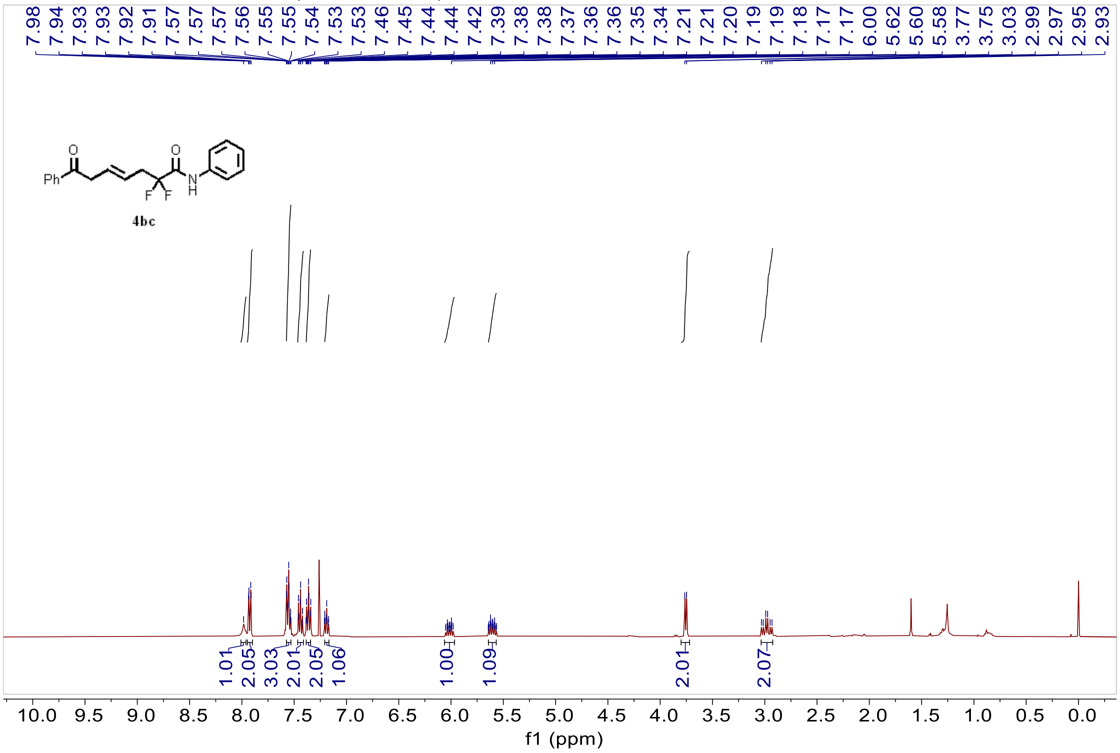

5810-TBH-2-88-3.10.fid 13C NMR (100 MHz, CDCl3)

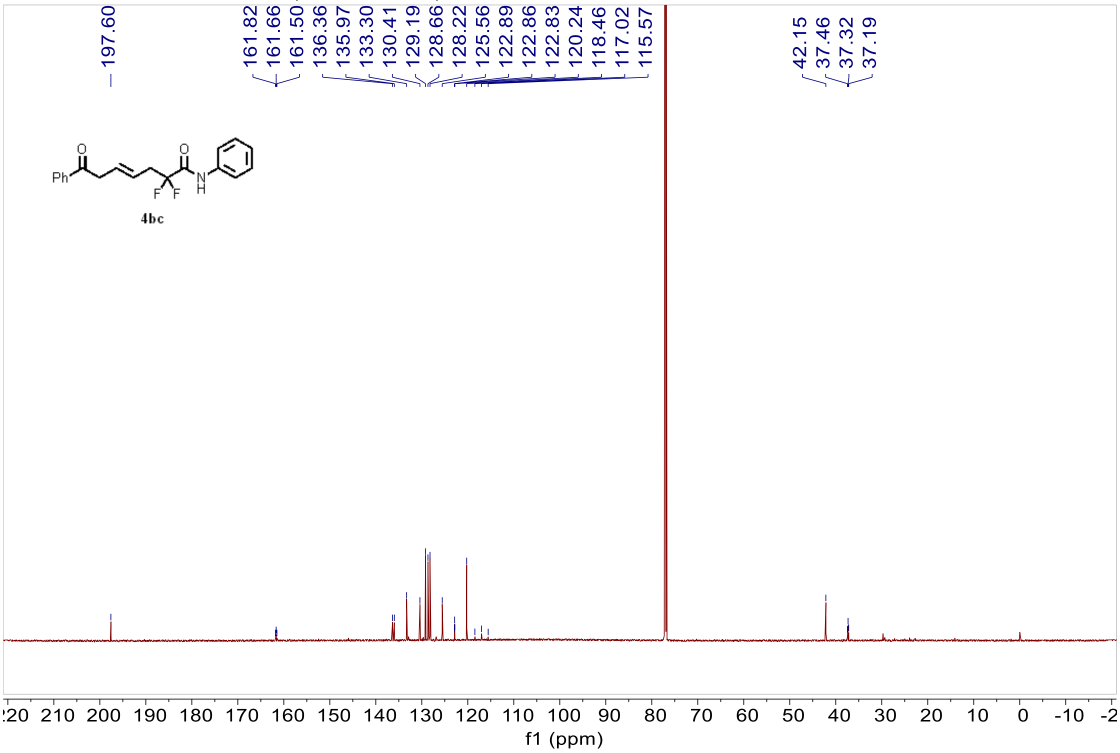

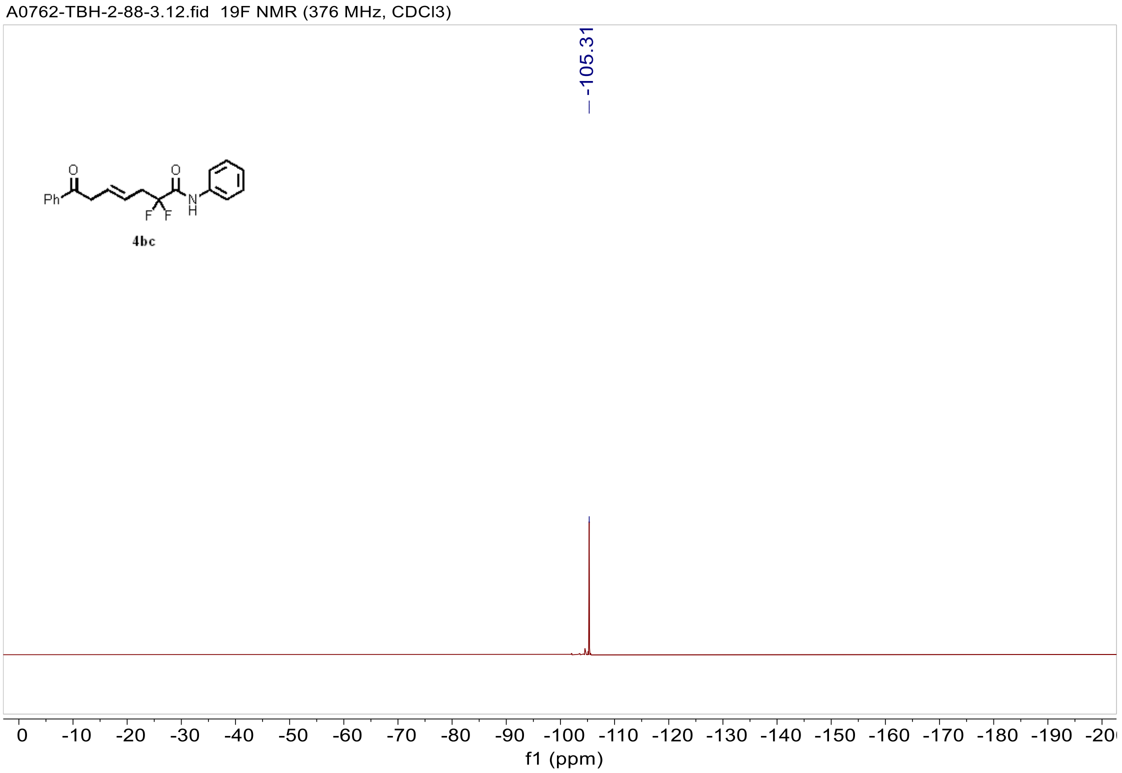
A0762-TBH-2-88-3.12.fid 19F NMR (376 MHz, CDCl3)
-105.31
Ph
O
O
N
H
F F
4bc
0 -10 -20 -30 -40 -50 -60 -70 -80 -90 -100 -110 -120 -130 -140 -150 -160 -170 -180 -190
f1 (ppm)

NMR spectrums of **4bd**

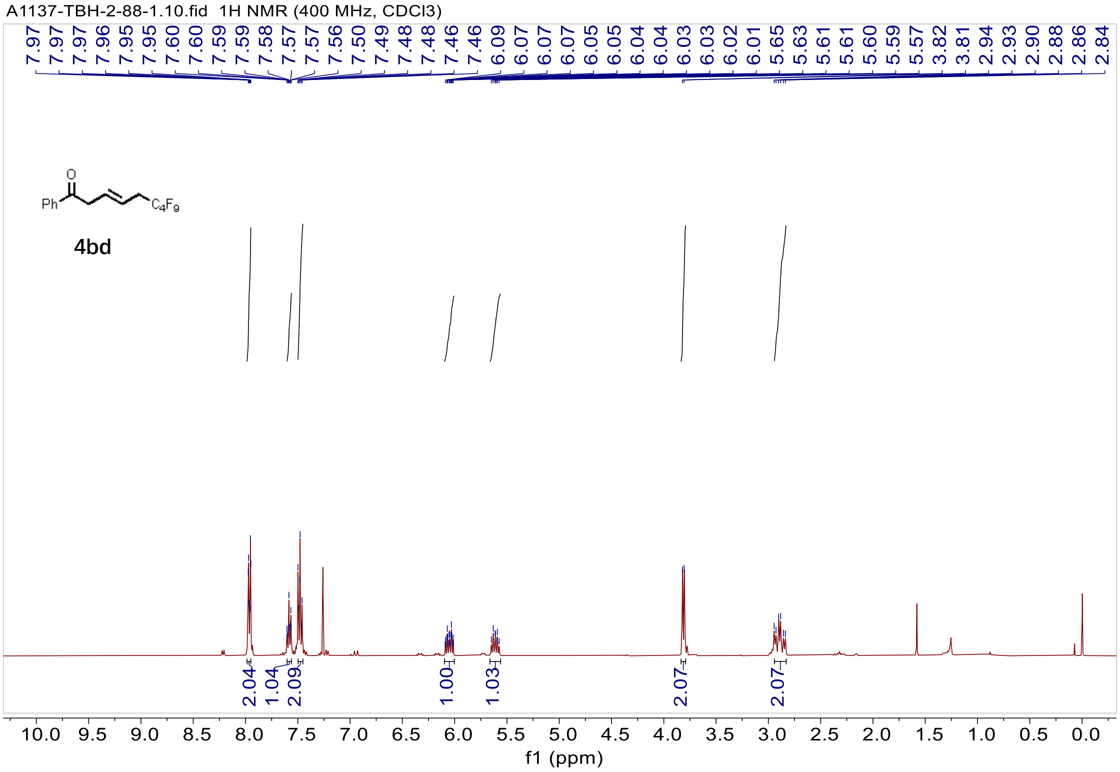

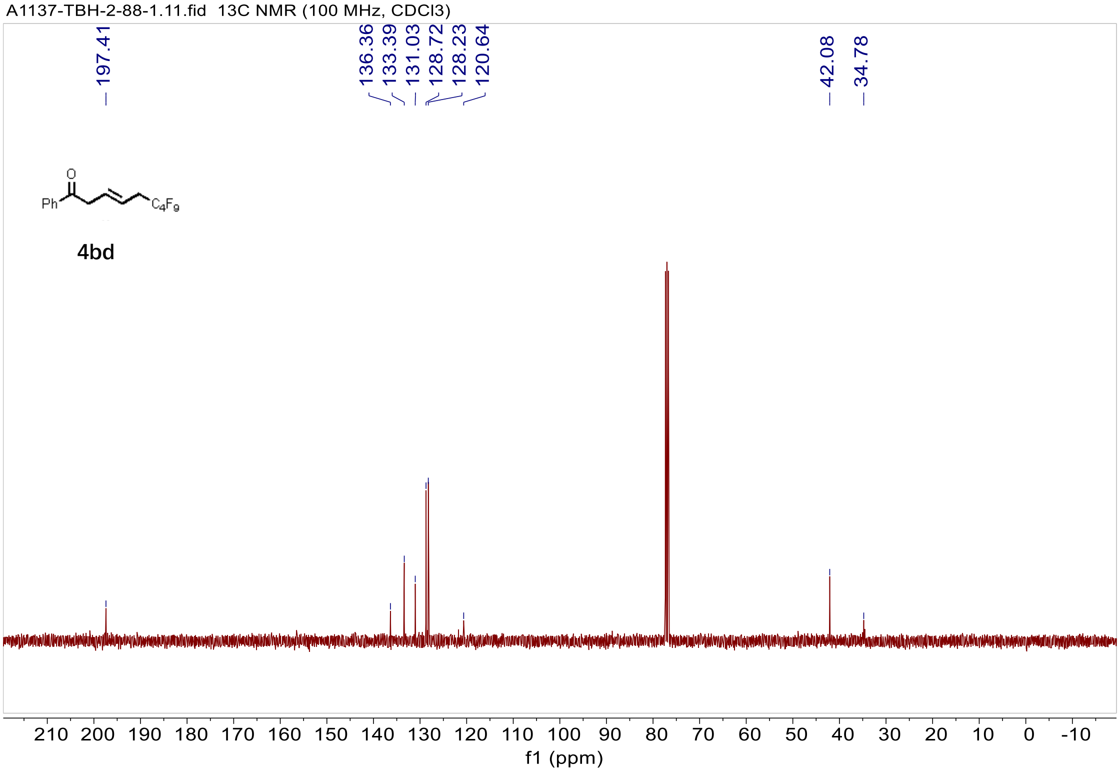

A1137-TBH-2-88-1.12.fid 19F NMR (376 MHz, CDCl3)

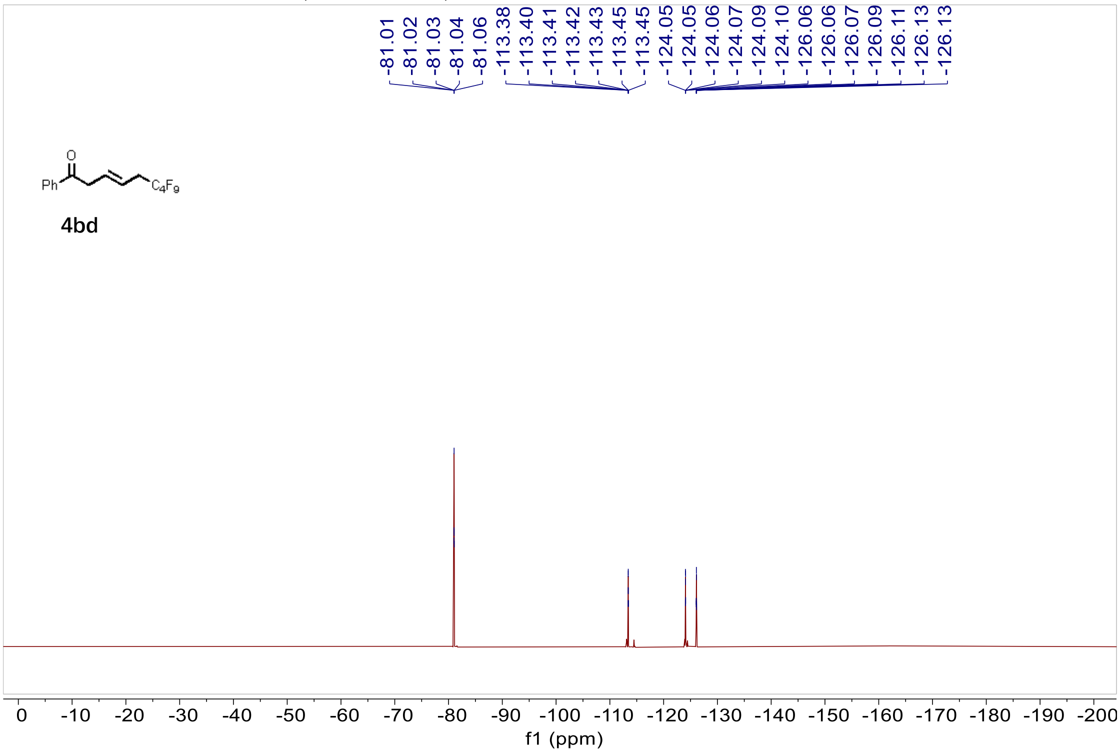

NMR spectrums of **4be**

A0613-TBH-2-88-4.10.fid 1H NMR (400 MHz, CDCl3)

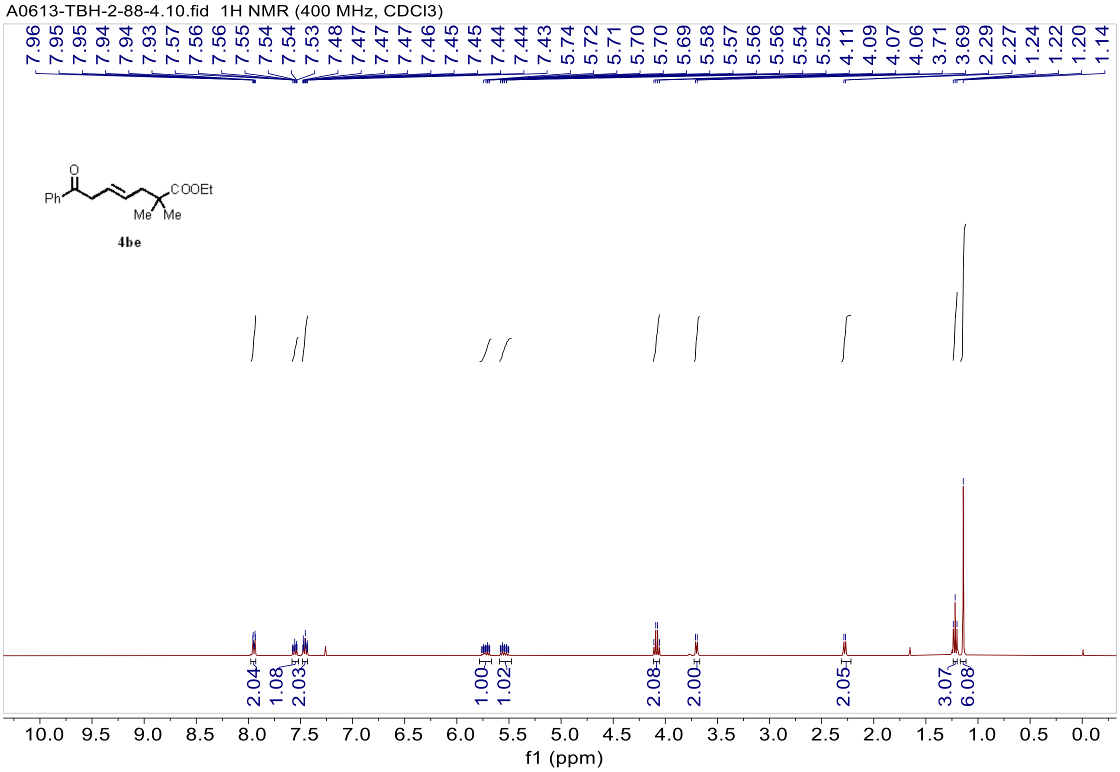

A0613-TBH-2-88-4.11.fid 13C NMR (100 MHz, CDCl3)

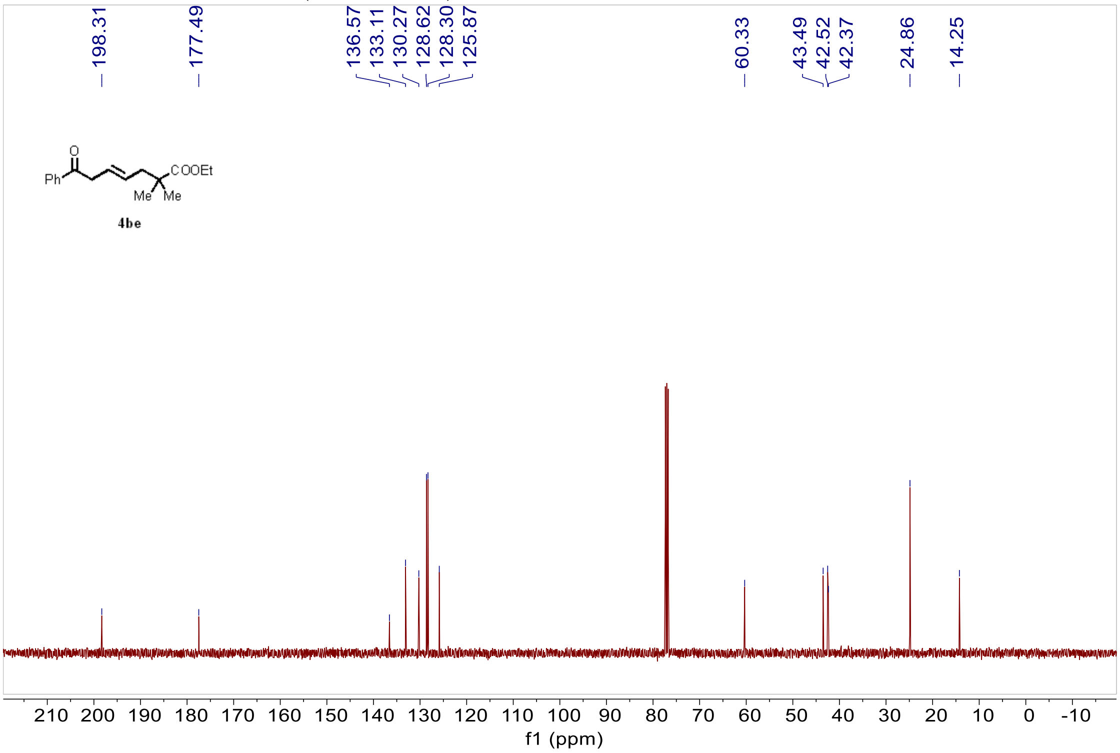

NMR spectrums of **4bf**

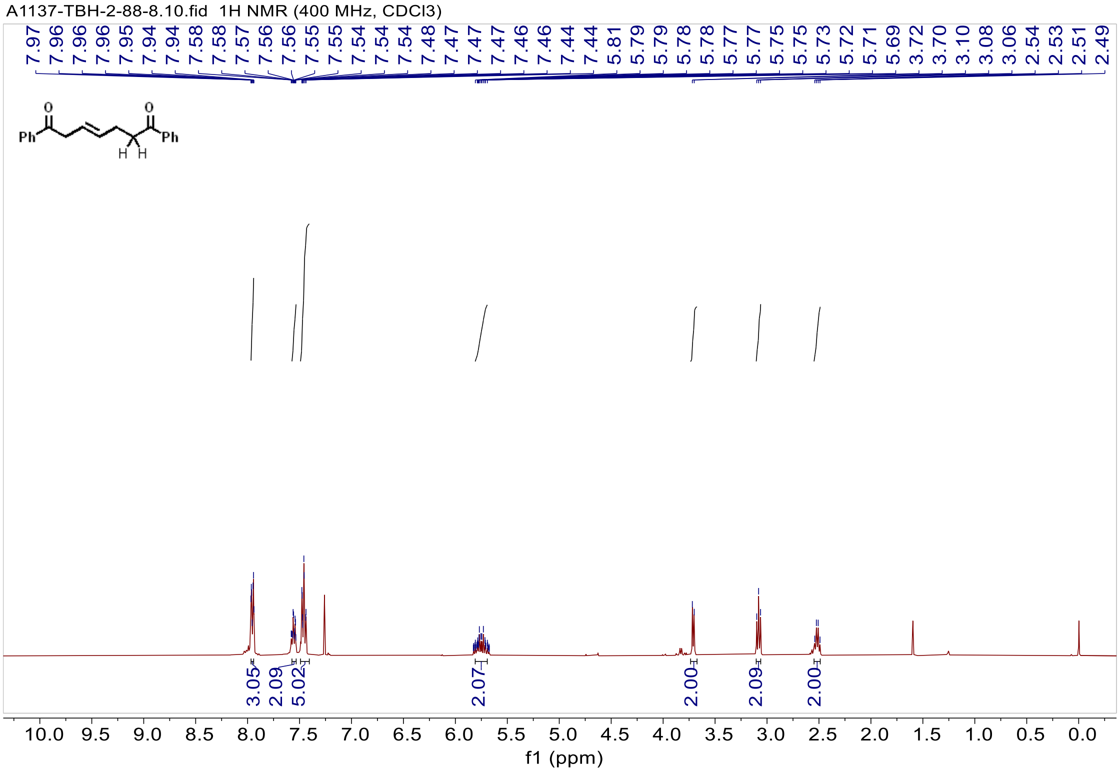

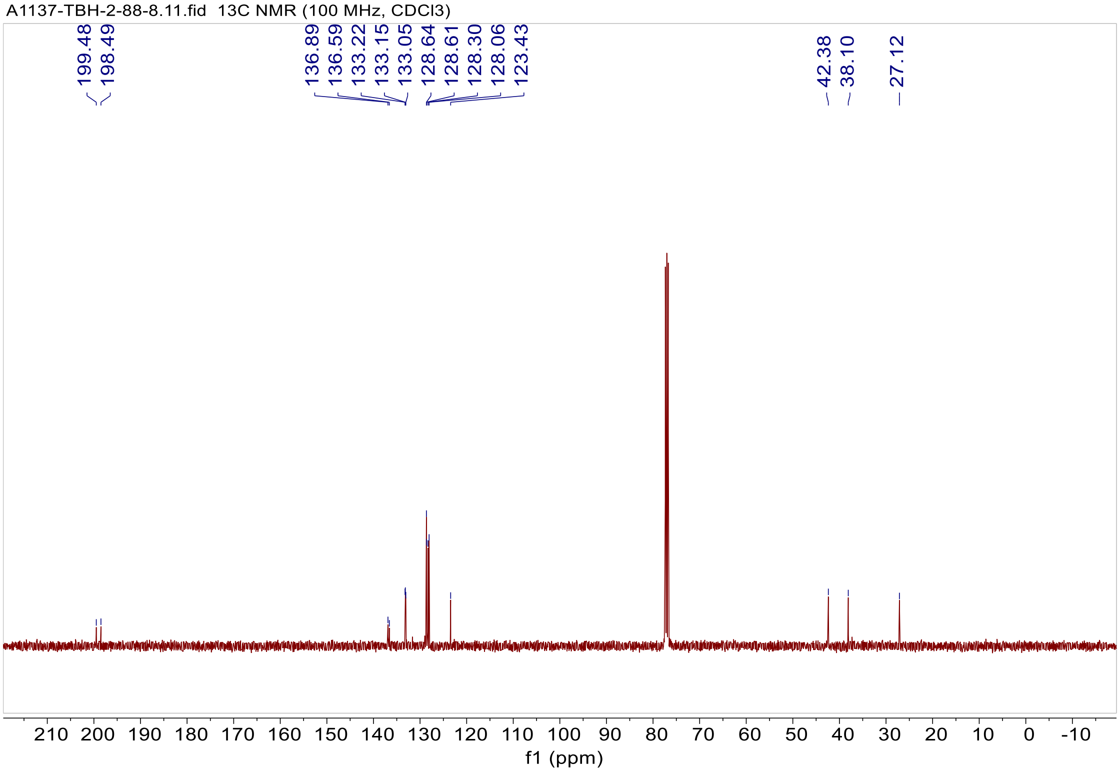

NMR spectrums of **4bg**

A1095-TBH-2-88-6-3.10.fid 1H NMR (400 MHz, CDCl3)

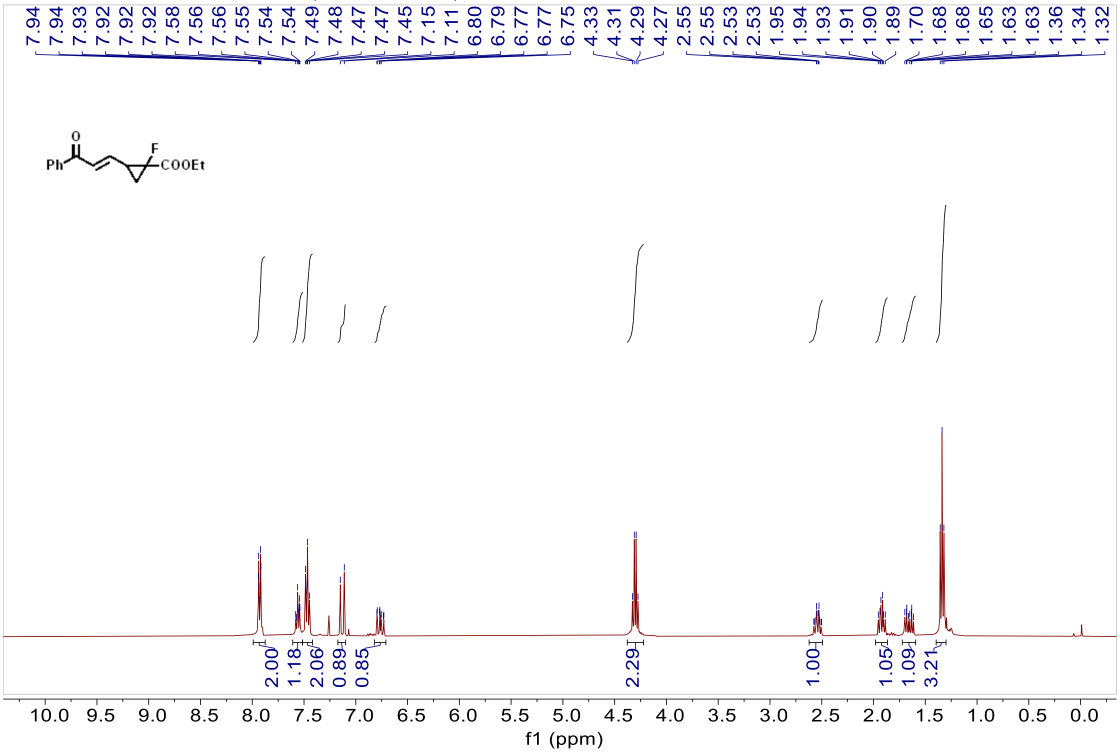

A1095-TBH-2-88-6-3.11.fid 13C NMR (100 MHz, CDCl3)

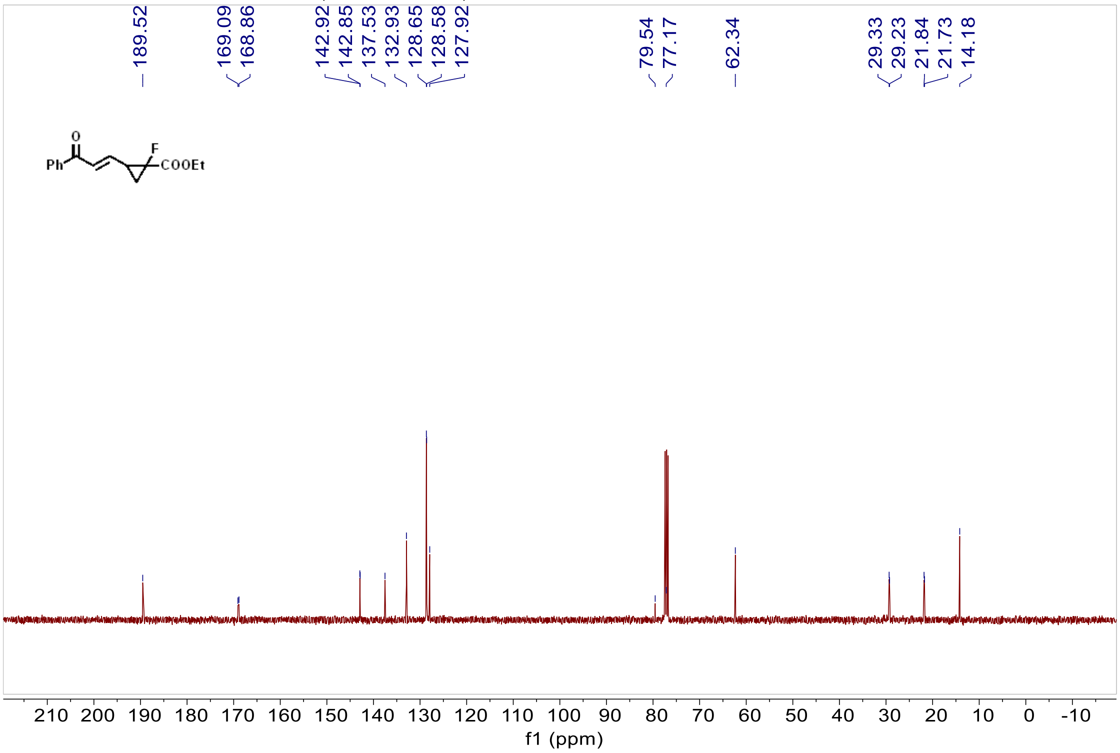

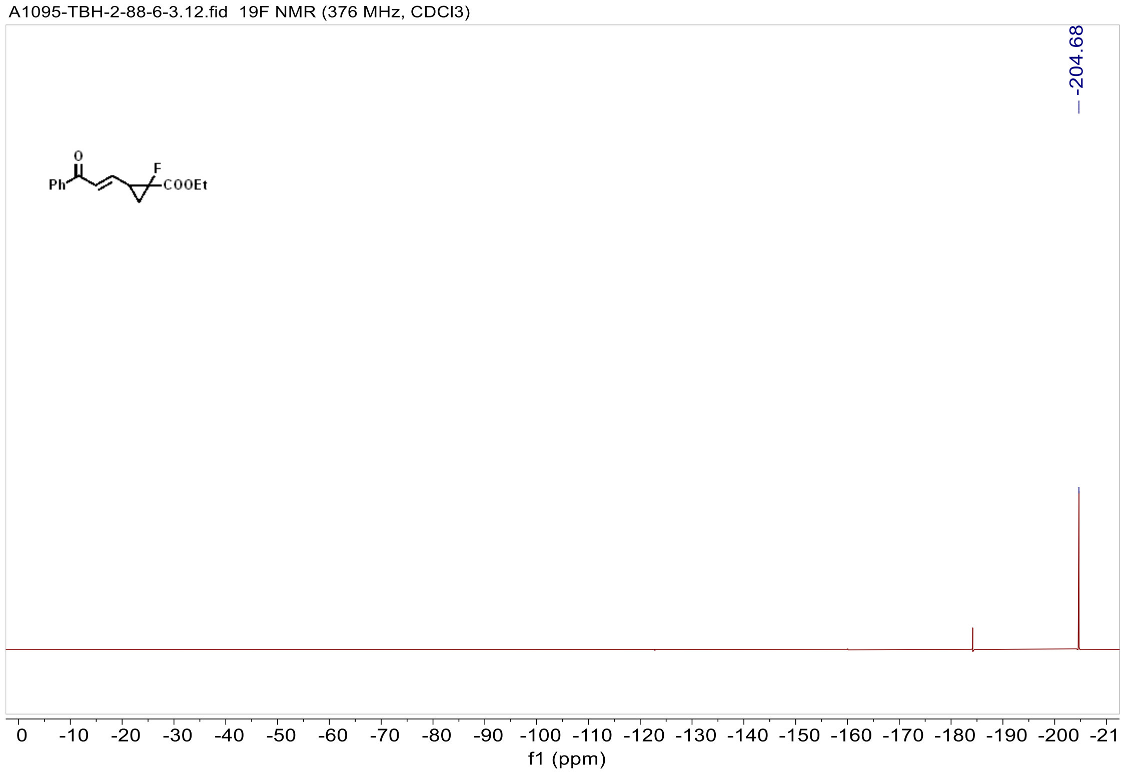
A1095-TBH-2-88-6-3.12.fid 19F NMR (376 MHz, CDCl3)
-204.68
O
Ph
F
COOEt
0 -10 -20 -30 -40 -50 -60 -70 -80 -90 -100 -110 -120 -130 -140 -150 -160 -170 -180 -190 -200 -21
f1 (ppm)

NMR spectrums of **4ca**

A1137-TBH-2-85-5.13.fid 1H NMR (400 MHz, CDCl3)

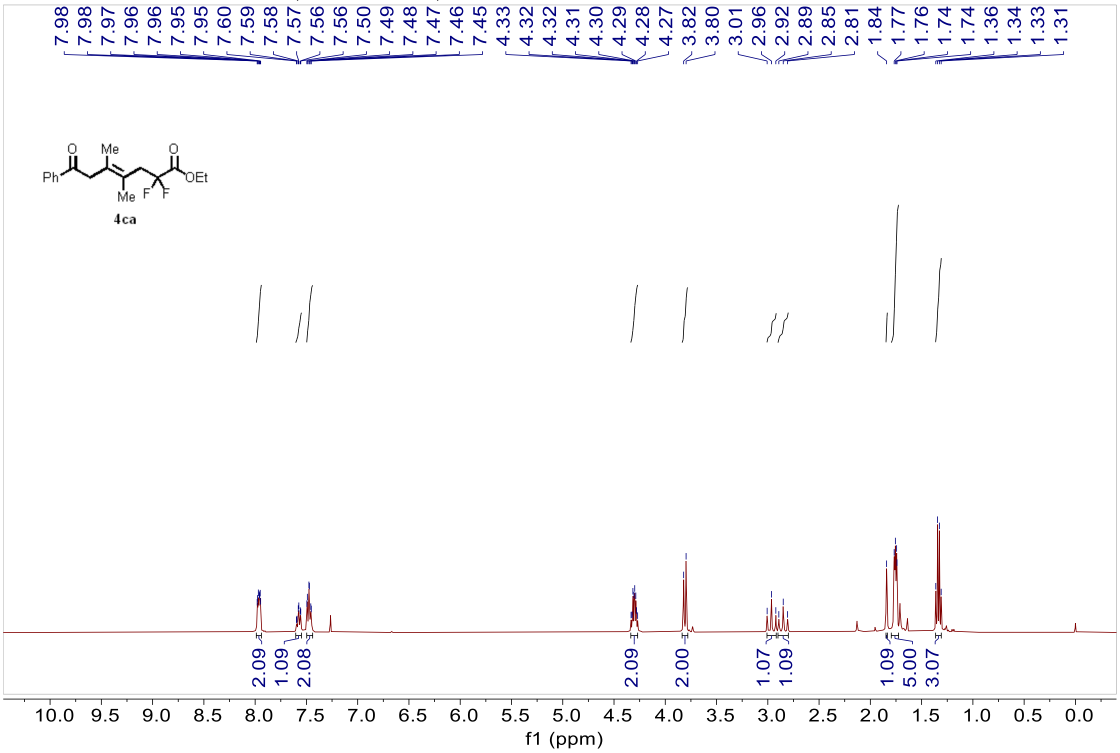

A1137-TBH-2-85-5.14.fid 13C NMR (100 MHz, CDCl3)

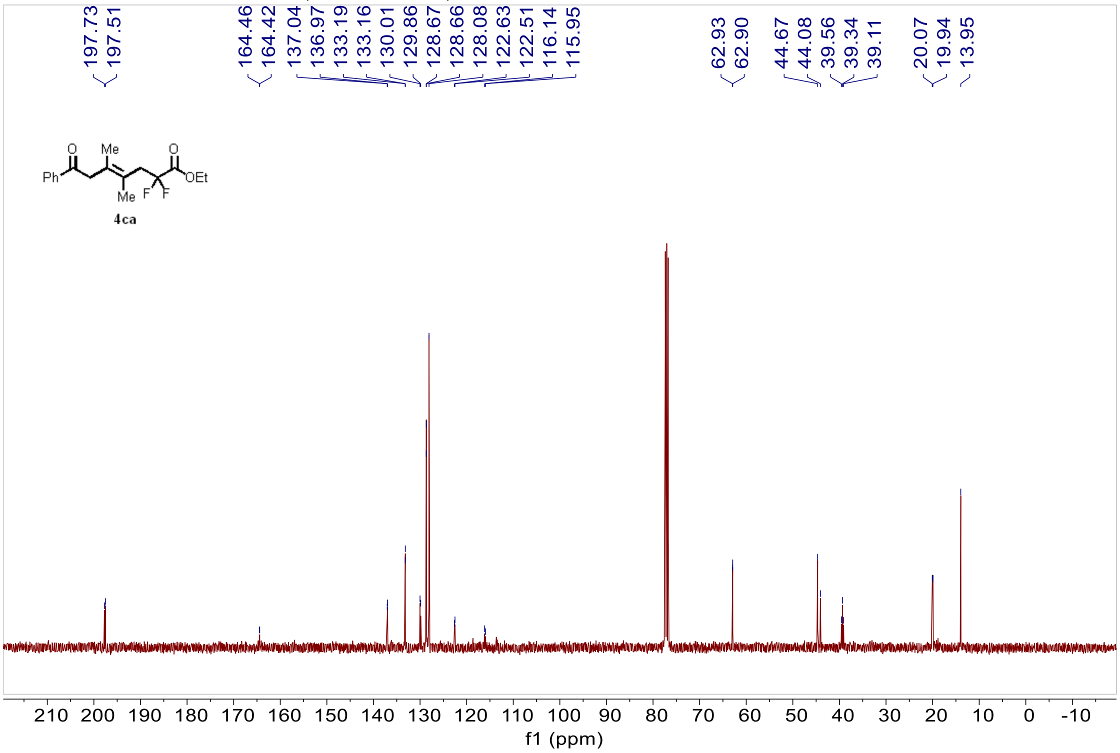

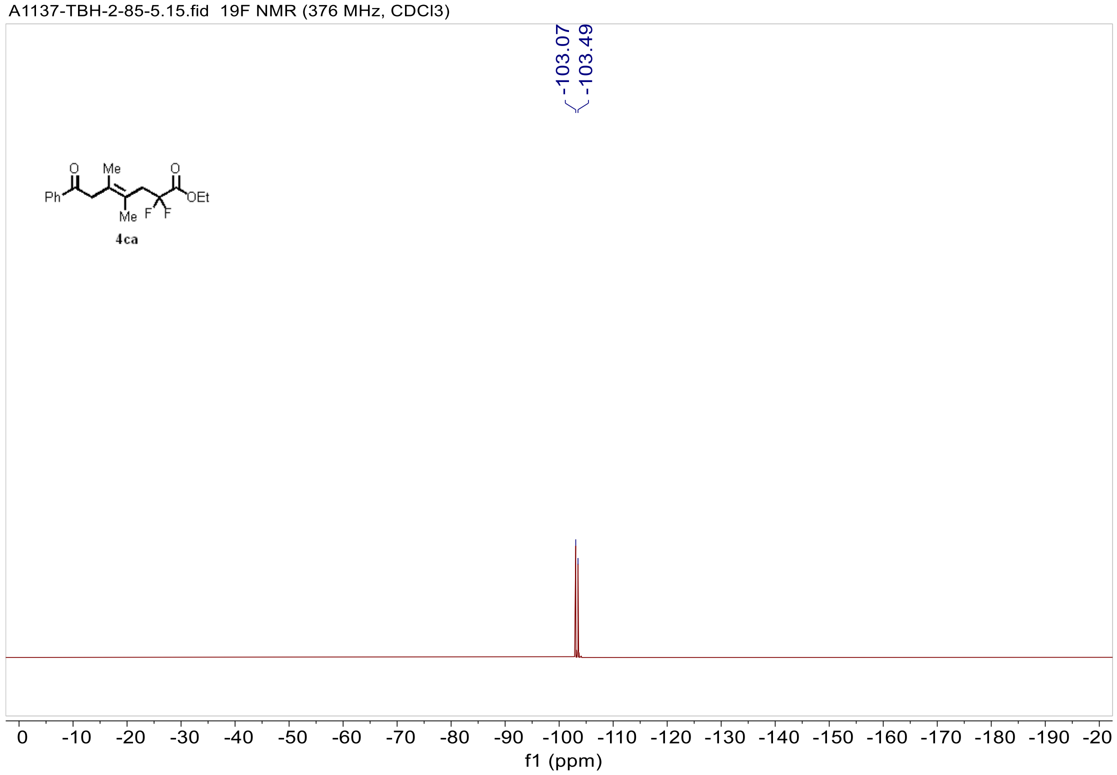
A1137-TBH-2-85-5.15.fid 19F NMR (376 MHz, CDCl3)
-103.07
-103.49
O
Me
O
Ph
OEt
Me
F
F
4ca
0 -10 -20 -30 -40 -50 -60 -70 -80 -90 -100 -110 -120 -130 -140 -150 -160 -170 -180 -190 -20
f1 (ppm)

NMR spectrums of **5aa**

A1914-TBH-2-88-3.10.fid 1H NMR (400 MHz, CDCl3)

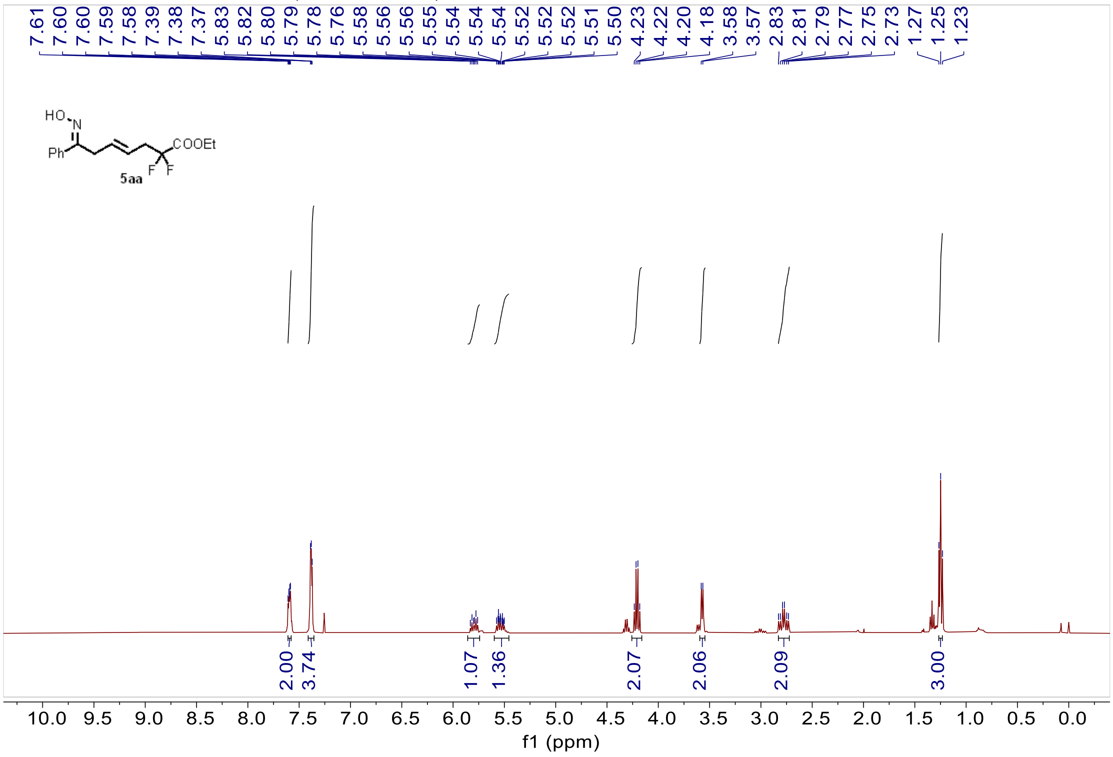

A1914-TBH-2-88-3.11.fid 13C NMR (100 MHz, CDCl3)

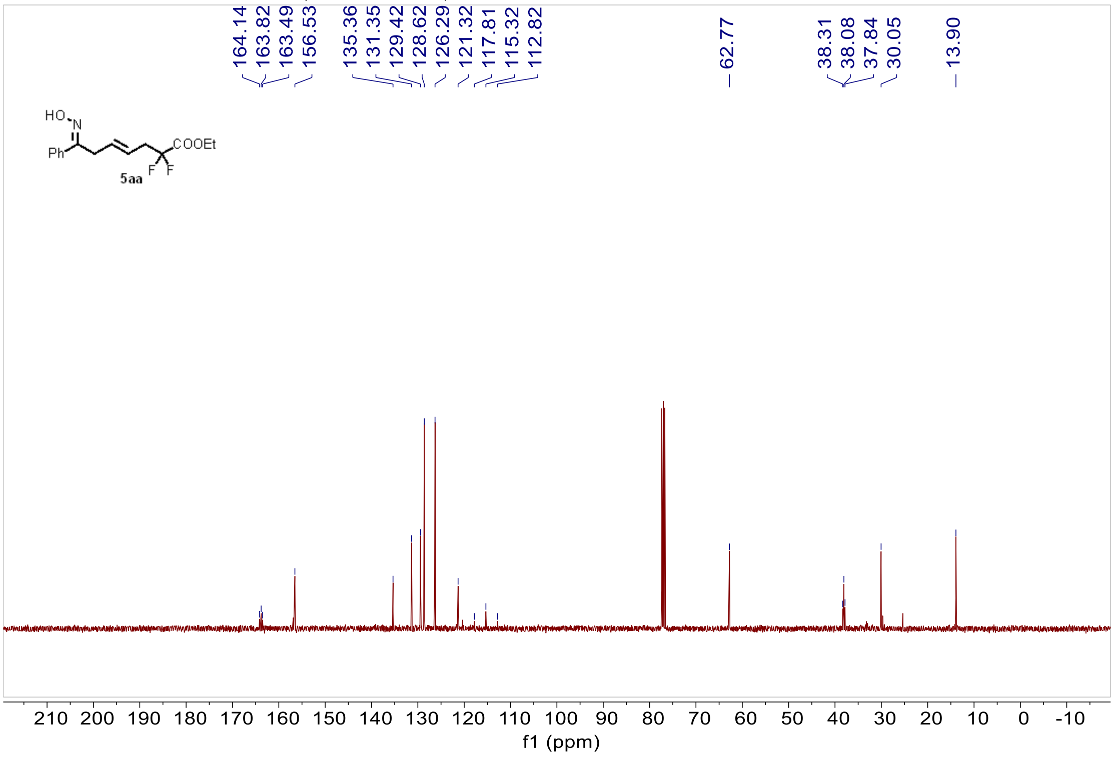

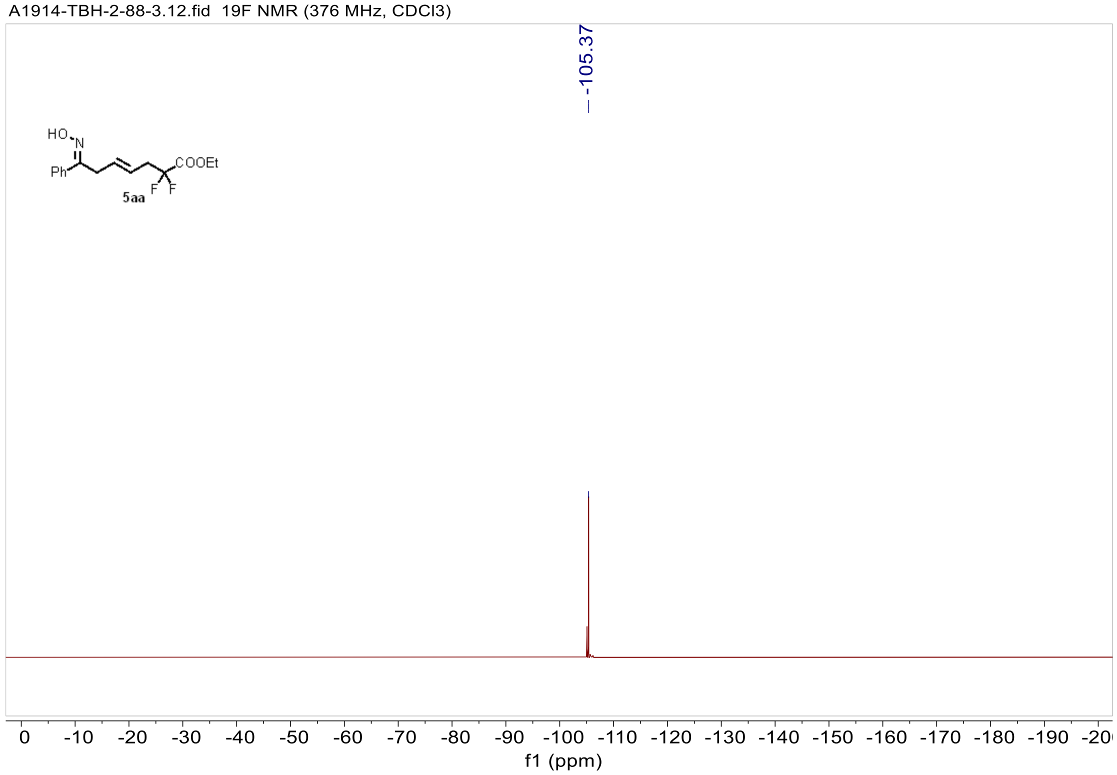
A1914-TBH-2-88-3.12.fid 19F NMR (376 MHz, CDCl3)
-105.37
HO
N
Ph
COOEt
F
F
5aa
0
-10
-20
-30
-40
-50
-60
-70
-80
-90
-100
-110
-120
-130
-140
-150
-160
-170
-180
-190
f1 (ppm)

NMR spectrums of **5ab**

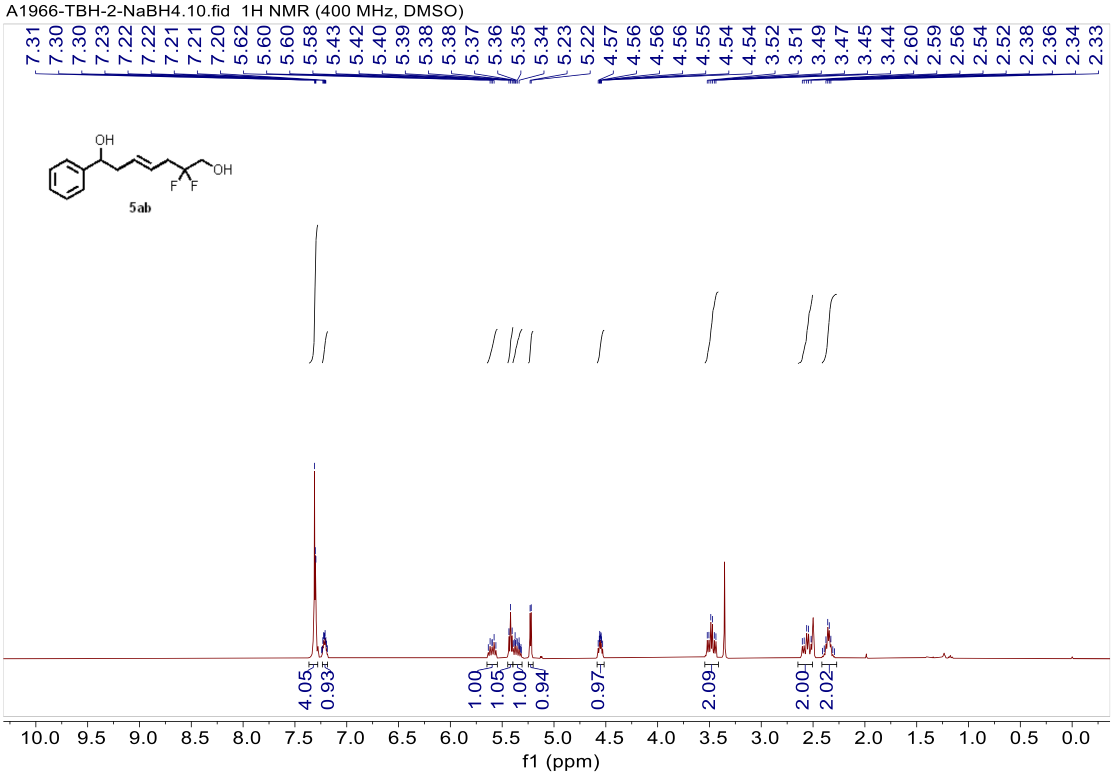

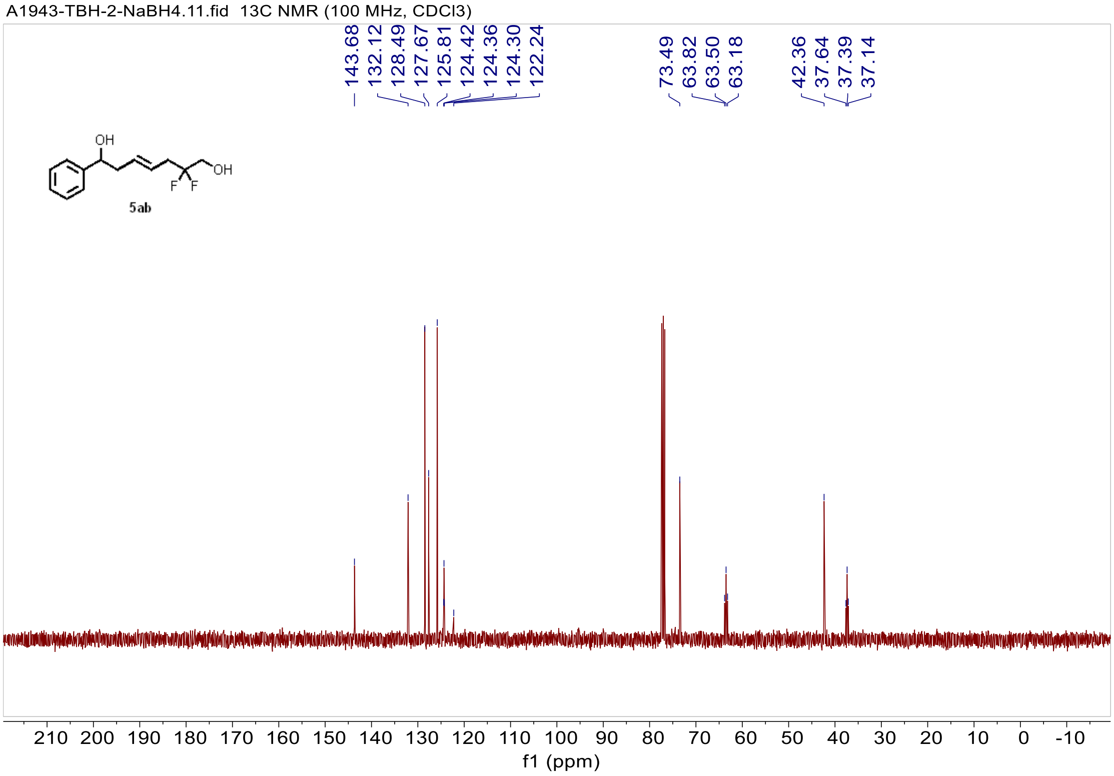

A1966-TBH-2-NaBH4.12.fid 19F NMR (376 MHz, DMSO)

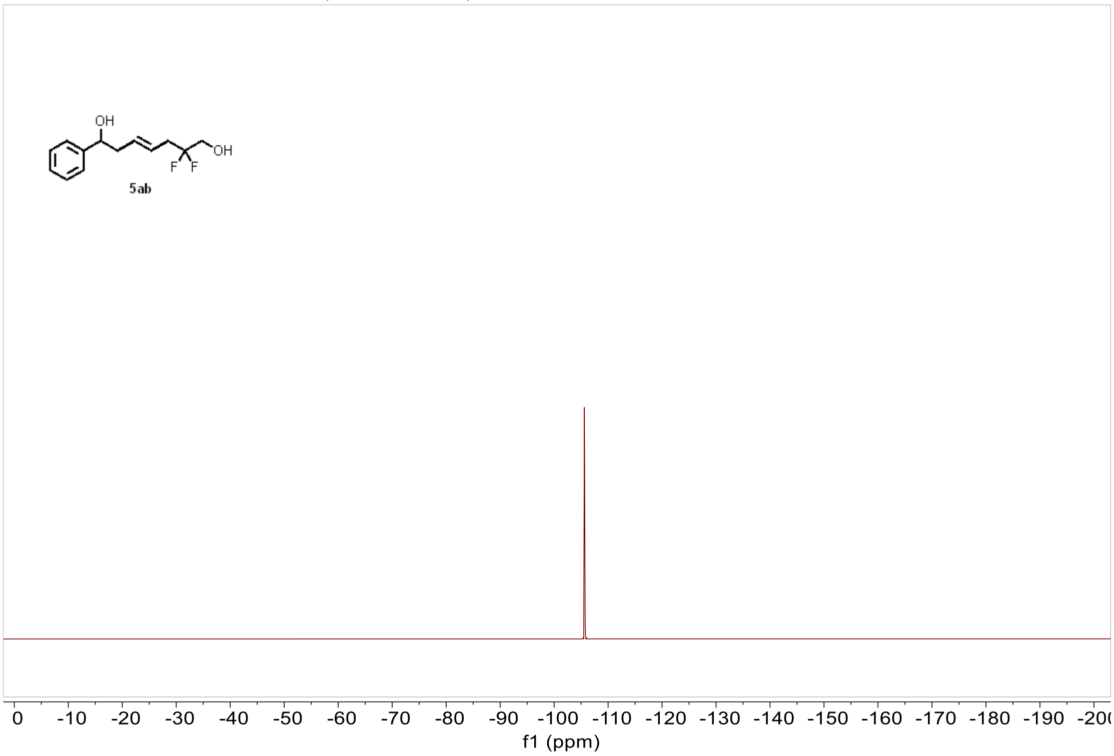

NMR spectrums of **5ac**

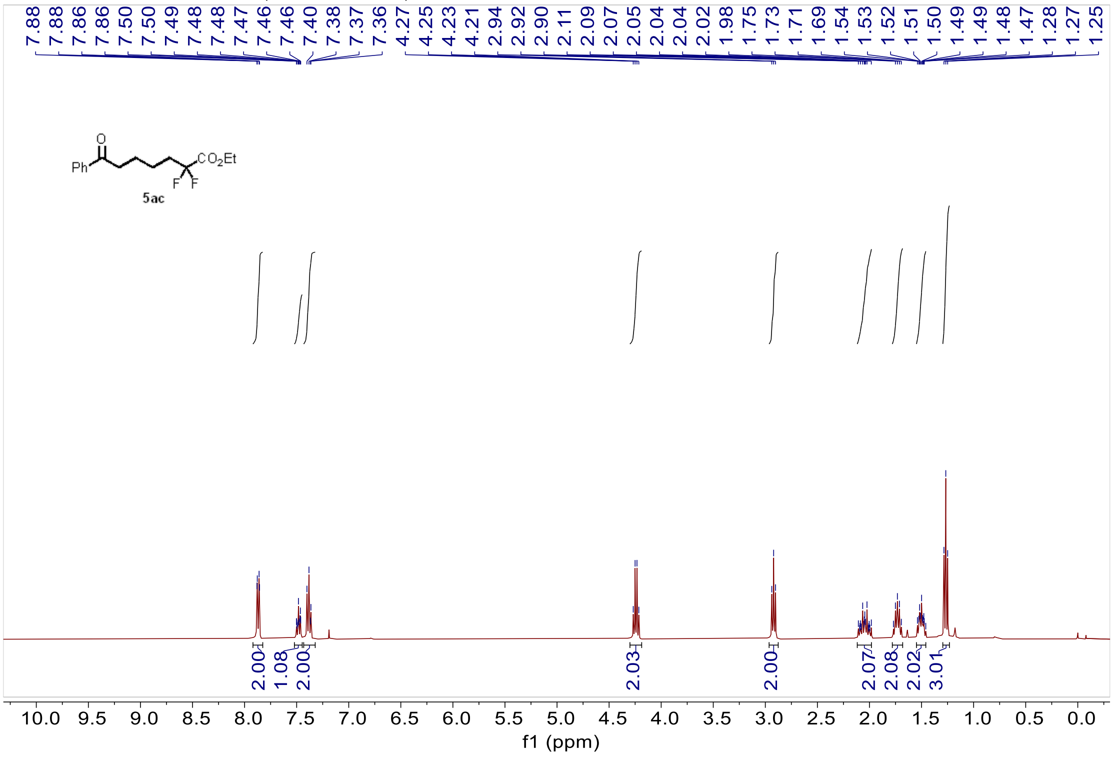

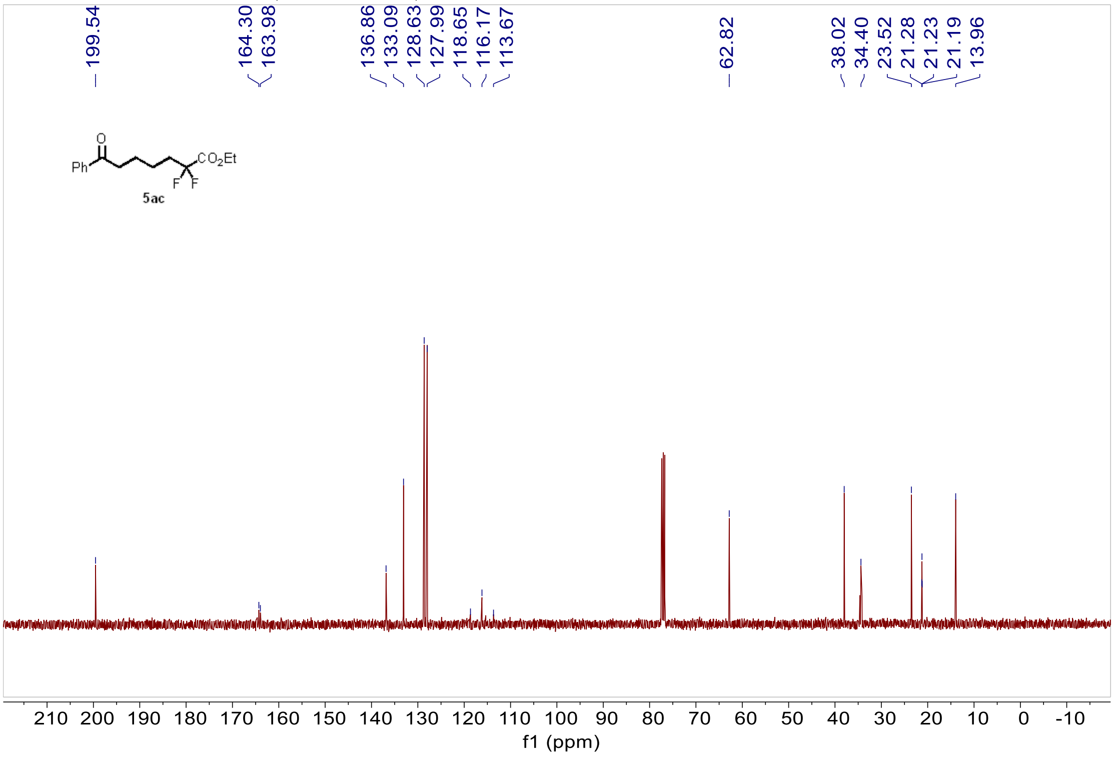

A2119-TBH-H2.11.fid 19F NMR (376 MHz, CDCl3)

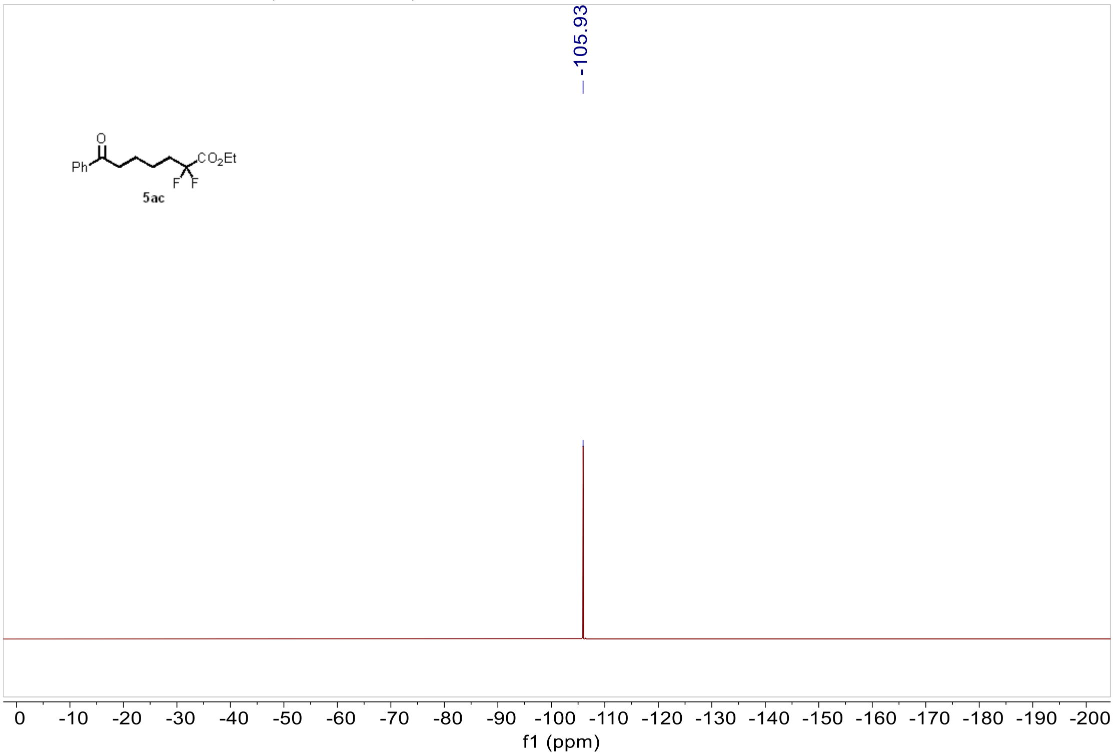

9. Reference

[1] Z.-P. Bao, Y. Zhang, X.-F. Wu, *Chem. Sci.* **2022**, *13*, 9387-9391.

[2] I. Triandafillidi, C. G. Kokotos, *Org. Lett.* **2016**, *19*, 106-109.

[3] G.-Q. Xu, T.-F. Xiao, G.-X. Feng, C. Liu, B. Zhang, P.-F. Xu, *Org. Lett.* **2021**, *23*, 2846-2852.

[4] Y. Yuan, X. Zhang, H. Qian, S. Ma, *Chem. Sci.* **2020**, *11*, 9115-9121.
